# Supplementary material for: Fc-Modified Antibody in Hospitalized Severe COVID-19 Patients
Source: Vaccines (Basel). 2025 Mar 31;13(4):372. doi: 10.3390/vaccines13040372 (PMC12031629; doi:10.3390/vaccines13040372)
Supplement: Supplementary file 1 [file vaccines-13-00372-s001.zip › Supplement 1.pdf]

Protocol Number SCTA01-B301

A Multicenter, Adaptive, Randomized, Double-blinded, Placebo-controlled Phase II/III Trial to Evaluate the Efficacy and Safety of Monoclonal Antibody SCTA01 against SARS-CoV-2 in Hospitalized Patients with Severe COVID-19

**Investigational Product Number:**	SCTA01
**Investigational Product Name:**	Recombinant anti-SARS-CoV-2 spike protein monoclonal antibody
**United States (US) IND Number:**	IND 152456
**Sponsor Contact (Medical):**	shuping_xu@sinocelltech.com<br>Tel: +86-010-58628288-9137
**Sponsor Contact (Study Operations):**	zhanghua_lan@sinocelltech.com<br>Tel: +86-010-58628288-8370
**SAE Reporting:**	Submit SAE Reports to:<br>SAEintake@covance.com,<br>CC: SCT-safety@sinocelltech.com

**Protocol Version**	**Date**
3.0	07 January 2021
4.0	02 July 2021
5.0	24 Aug 2021

PROTOCOL SYNOPSIS

**Name of Sponsor/Company:**<br>Sinocelltech, Ltd.
**Name of Investigational Product:**<br>Recombinant anti-SARS-CoV-2 spike protein monoclonal antibody (Product Code: SCTA01)
**Name of Active Ingredient:**<br>Recombinant humanized anti-SARS-CoV-2 monoclonal IgG1 antibody (SCTA01)
**Title of Study:**<br>A multicenter, adaptive, randomized, double-blinded, placebo-controlled Phase II/III trial to evaluate the efficacy and safety of monoclonal antibody SCTA01 against SARS-CoV-2 in hospitalized patients with severe COVID-19
**Study Number:**<br>SCTA01-B301
**Study Phase:**<br>Phase II/III
**Study Population:**<br>Hospitalized patients with severe COVID-19 (5 point on the 8-point ordinal scale).<br>According to the NIH definition, severe patients are those individuals who have one of the following: 1) respiratory rate > 30 breaths per minute; 2) pulse oxygen saturation (SpO_2) ≤ 93% on room air at sea level; 3) PaO_2/FiO_2 < 300 mmHg or SpO_2/FiO_2≤315 mmHg; 4) lung infiltrates > 50%. 5 point: Hospitalized, requiring supplemental oxygen.
**Primary Objectives:**<br>● To evaluate the clinical efficacy of SCTA01 as assessed by time to clinical improvement (Phase II, III).<br>**Secondary Objectives:**<br>● To evaluate the clinical efficacy of SCTA01 as assessed by clinical severity, hospitalization and mortality (Phase II, III);<br>● To evaluate the safety of SCTA01 (Phase II, III);<br>● To evaluate the virologic efficacy of SCTA01 (Phase II, III);<br>● To evaluate the pharmacokinetics of SCTA01 (Phase II);<br>● To evaluate the immunogenicity of SCTA01 (Phase II, III).

Exploratory Objectives:

- To explore the correlation between the baseline serological antibody level and therapeutic effect (Phase II, III);
- To evaluate the virologic efficacy of SCTA01 as assessed by quantitative SARS-CoV-2 virus in patient's blood (Phase II, III).

Primary Endpoints:

- Time to clinical improvement (TTCI) up to Day 29. TTCI is defined as the time (in days) from randomization to the first day on which a patient satisfies point 1, 2, or 3 on the 8-point ordinal scale and maintains a score ≤ 3 at least 48 hours (initial improvement) and maintains this up to Day 29 (sustained improvement) (Phase II, III).

Secondary Endpoints:

- Clinical efficacy of SCTA01:
  - Proportion of patients with initial clinical improvement at Day 29 (Phase II, III);
  - Proportion of patients with sustained clinical improvement at Day 29 (Phase II, III);
  - Time to initial clinical improvement up to Day 29 (Phase II, III);
  - Time to discharge from hospital, followed by being alive and home for 14 consecutive days prior to Day 90 (Phase II, III);
  - Proportion of patients discharged from hospital, followed by being alive and home for 14 consecutive days prior to Day 90 (Phase II, III);
  - All-cause mortality up to Day 29, record date and cause of death (if applicable) (Phase II, III);
  - Percentage of subjects in each category on the 8-point ordinal scale (time frame: Days 3, 5, 8, 11, 15, 22, and 29) (Phase II, III);
  - Proportion of patients alive and free of respiratory failure up to Day 29 (Phase II, III);
  - Proportion of patients who are discharged from acute care up to Day 29 (Phase II, III);
  - Days of supplemental oxygen (if applicable) up to Day 29 (Phase II, III);
  - Days of non-invasive ventilation/high flow oxygen (if applicable) up to Day 29 (Phase II, III);
  - Days of invasive mechanical ventilation/extracorporeal membrane oxygenation (ECMO) (if applicable) up to Day 29 (Phase II, III);
  - Time to hospital discharge up to Day 29 (Phase II, III);
  - Number and proportion of non-responders having mutations in the gene encoding the SARS-CoV-2 S protein through Day 29. A non-responder is defined as a patient who does not achieve a reduction in viral shedding or

persistence viral presence or have a rebound in viral shedding following suppression (Phase II, III).

- Safety of SCTA01:
  - Cumulative incidence of serious adverse events (SAEs) (Phase II, III);
  - Cumulative incidence of Grade 1, 2, 3 and 4 clinical and/or laboratory adverse events (AEs) (Phase II, III);
  - Discontinuation or temporary suspension of infusions (for any reason) (Phase II, III);
  - Chemistries, hematology, urinalysis, coagulation test, IL-6, ferritin, procalcitonin, thrombin time, D-dimer (Phase II, III);
  - Number and proportion of patients who are suspected to experience ADE (Phase II, III).
- Virologic efficacy of SCTA01:
  - Change from baseline in viral shedding as measured by quantitative reverse transcription polymerase chain reaction (RT-qPCR) in NP or OP swab samples (Phase II, III);
  - Time-weighted average change from baseline between SCTA01 group and placebo group in viral shedding as measured by RT-qPCR in NP or OP swab samples (Phase II, III);
  - Time to SARS-CoV-2 RNA negative (NP or OP swab samples) as determined by RT-qPCR (Phase II, III).
- Pharmacokinetics (PK) of SCTA01:
  - Pharmacokinetic characteristics in severe COVID-19 patients (Phase II).
- Immunogenicity of SCTA01:
  - Immunogenicity assessment includes the number and percentage of subjects who develop detectable anti-drug antibodies (ADA) (Phase II, III).

Exploratory Endpoints:

- The correlation between the baseline serological antibody level and therapeutic effect (Phase II, III);
- Quantitative SARS-CoV-2 virus in patient's blood (Phase II, III).

Study Design:

The study is a multicenter, adaptive, randomized, double-blinded and placebo-controlled Phase II/III trial, and will be conducted at selected investigational sites globally. The study is comprised of 2 parts: dose selection (Phase II) and pivotal treatment effect (Phase III).

Phase II part will evaluate the efficacy, safety and PK of SCTA01 15 mg/kg and 50 mg/kg in severe patients with COVID-19. The primary endpoint will evaluate the difference of TTCI among study group 1 [SCTA01 15 mg/kg + best supportive care (BSC)], study group 2

(SCTA01 50 mg/kg + BSC) and control group (placebo + BSC) up to Day 29. The secondary endpoint will evaluate the difference of virologic efficacy among study group 1 (SCTA01 15 mg/kg + BSC), study group 2 (SCTA01 50 mg/kg + BSC) and control group (placebo + BSC) from baseline to Day 8, and then determine the recommended dose of Phase III part.

In the Phase II part of the study, 285 subjects will be randomized by 1:1:1 ratio to 15 mg/kg group, 50 mg/kg group and placebo group, with 95 subjects in each group.

At the end of Phase II part, enrollment will be paused and the interim analysis for dose selection will be performed after the 285th subject has been observed for TTCI. An Independent Data Monitoring Committee (IDMC) will assess the benefit and risk to decide a dose for the Phase III part of the study.

Enrollment will be resumed after the IDMC has completed their evaluation and recommended the dose for the Phase III part.

The Phase III part will evaluate the efficacy, safety, and immunogenicity of SCTA01 at the dose recommended by the IDMC (X mg/kg or X mg) in hospitalized patients with severe COVID-19. The primary endpoint will evaluate the difference of TTCI between study group (SCTA01 X mg/kg or X mg + BSC) and control group (placebo + BSC) up to Day 29.

Approximately 510 subjects in Phase III part will be randomized by 1:1 ratio to SCTA01 (X mg/kg or X mg) group and placebo group, with 255 subjects in each group. IDMC will be used to monitor safety and efficacy of the study.

Total number of Phase II/III study is about 795.

Interim analysis (IA): There will be three un-blinded interim analyses when the 143rd patient in Phase II has completed Day 8 visit, at the end of Phase II and when the 160th patient in Phase III has completed Day 29 or Early Withdraw visit.

- The 1st interim analysis is for safety evaluation. The safety data will be reviewed at the 1st interim analysis, but the preliminary review of efficacy data is supported if early data and external emerging data warrants.
- The 2nd interim analysis is for dose selection: the analysis will be performed at the end of Phase II when the 285th patient completed Day 29 or Early Withdraw visit. The dose selected to continue to the Phase III part will be the dose with the greater effect in reduction of viral load compared to placebo at Day 8. Time to clinical improvement of each dose will be evaluated and the RR threshold of 1.15 will be used as a futility boundary at the end of Phase II. The futility boundary is non-binding, and the IDMC may recommend study continuation even if the futility bound is met on TTCI. If any safety signal is observed for any dose, the IDMC will weigh this information against the efficacy results in selecting the dose for Phase III.
- The 3rd interim analysis is the penultimate analysis that allows for stopping for superior efficacy. It is planned when 160 patients in Phase III (approximately 31% information time of Phase III part, or equivalently 50% of the TTCI events combined from phase II and phase III part of the study) have been documented in the ITT population. If the interim analysis is performed at 31% of information time of Phase III, based on Pocock type boundary of α-spending function, a 1-sided p-value less than 0.01068 using inverse normal

method to combine p-value from Phase II part and Phase III part will need to be observed to declare statistical significance. A futility boundary of an observed RR of 1.22 will be used to declare failure of the study when TTCI events from Phase II and Phase III part combined are exactly 280, which is equivalent to an observed one-sided p-value of 0.05.

Note: The critical values of nominal p-value and hazard ratio that will be used to declare statistical significance and/or futility at the interim analyses will be calculated based on the actual number of the TTCI events documented at the time the interim analysis.

The interim analyses will be performed by an independent statistician external to the study team. The results of the interim analyses will be presented to the IDMC by the independent statistician. The IDMC will provide the Sponsor with a recommendation to continue the trial as planned, to have an interruption, to make a protocol amendment, or to terminate the trial. If the interim analysis demonstrates significantly shortened TTCI in the SCTA01 group compared to the control group, the IDMC could recommend to terminate and un-blind the study, and the study could be announced as completed.

Main Inclusion Criteria:

- Hospitalized patients with severe COVID-19 (5 point on the 8-point ordinal scale). According to the NIH definition, severe patients are those individuals who have one of the following: 1) respiratory rate > 30 breaths per minute 2) $SpO_2 \leqslant 93\%$ on room air at sea level; 3) $PaO_2/FiO_2 < 300$ mmHg or $SpO_2/FiO_2 \leqslant 315$ mmHg; 4) lung infiltrates > 50%. In high altitude area (over 1000 meters), PaO_2/FiO_2 should be corrected according to the following formula: $PaO_2/FiO_2 \times$ [760/barometric pressure (mmHg)]; 5 point: Hospitalized, requiring supplemental oxygen;
- Male or female adult $\geqslant$18 years of age at time of enrollment;
- Has documented laboratory-confirmed SARS-CoV-2 infection as determined by PCR. Note: Patient can be enrolled before SARS-CoV-2 infection has been confirmed and can be tested by any approved clinical test other than PCR as long as SARS-CoV-2 infection is then confirmed by PCR;
- $\leqslant$ 14 days since symptoms of COVID-19 onset;
- Eligible COVID-19 vaccinated patients at the time of enrollment are allowed.

Main Exclusion Criteria:

- Patients who need non-invasive ventilation or high flow oxygen (i.e., 6 point on the 8-point ordinal scale);
- Patients with critical COVID-19. According to the NIH definition, critical patients are those individuals who have one of the following conditions: 1) respiratory failure and need invasive mechanical ventilation; 2) septic shock; 3) multiple organ dysfunction；
- Patients with severe COVID-19 who received convalescent plasma or anti-SARS-CoV-2 spike (S) protein targeted therapy;
- Alanine-amino transferase (ALT) or aspartate transaminase (AST) is 5 times higher than the upper limit of the normal value;

- Estimated glomerular filtration rate (eGFR) ＜30 mL/min or on dialysis {eGFR calculated by Cockcroft-Gault formula (*Cockcroft DW, 1976*), Male: CrCL (mL/min) = [(140 - age) × weight (kg)] × 1/ [SCr (mg/dL) × 72]; Female: CrCL (mL/min) = [(140 - age) × weight (kg)] × 0.85/ [SCr (mg/dL) × 72]}.

Investigational Product, Dosage, and Mode of Administration:

SCTA01 will be provided in glass vial. Each vial contains 10 mL of study medication with a concentration of 25 mg/mL.

Placebo is identical in physical appearance to that of SCTA01 injection and contains the same inactive ingredients which include histidine, sodium chloride, polysorbate 80, hydrochloric acid and injection grade water.

SCTA01/Placebo should be stored under refrigeration at 2~8°C (36-46°F) in the original carton to protect from light and avoid repeated freezing/thawing.

Each participant will receive a single intravenous infusion of either SCTA01 or placebo. Infusion volume will be 500 mL and infusion time is 3 hours (±10 minutes).

Detailed infusion information will be in the Pharmacy Manual.

Duration of Treatment:

Single dose of drug.

Reference Therapy, Dosage, and Mode of Administration:

Best supportive care is allowed as a base therapy except the use of convalescent plasma, or anti-SARS-CoV-2 spike (S) protein targeted therapy. Refer to local clinical routine for the treatment of patients with severe COVID-19.

Criteria for Evaluation:

Efficacy: 8-Point ordinal scale.

Safety: Division of AIDS (DAIDS) Table for Grading the Severity of Adult and Pediatric Adverse Events (Version 2.1, July 2017).

Statistical Methods:

- All enrolled subjects set: All patients who sign the informed consent form (ICF) will be registered in the electronic data collection (EDC) system. Cases that are included in the study but not administered study drug will be analyzed using this analysis set.
- ITT set: All patients who are randomly assigned to and receive a dose of study drug. This is the primary analysis set for the efficacy analyses, and patients will be analyzed according to the treatment to which they are randomly assigned.
- Full analysis set (FAS): All patients who are randomly assigned, receive a dose of study drug and have at least one primary endpoint measurement. This analysis set will support the robustness of the primary efficacy results and patients will be analyzed according to the treatment to which they are randomly assigned.

- Safety set (SS): All patients who are randomly assigned and receive a dose of study drug. All safety analyses will be based on the safety set, with patients analyzed according to the treatment they received.
- Pharmacokinetics set (PKS): Subjects that receive single-dose investigational drug and have at least one blood-drug concentration data point which can be analyzed. PKS consists of PK Concentration set (PKCS) and PK Parameter set (PKPS).
- Per-protocol set (PPS): All patients in the ITT set without any major protocol deviations which may affect efficacy evaluation. The Per-protocol combined set will be used for sensitivity analysis of the primary endpoint.

Safety Analysis

The safety summaries will include treatment group and placebo group, comprising all patients in the safety set.

Adverse events will be tabulated by treatment group. The number and percentage of patients with at least one AE will be presented along with a breakdown by system organ class and preferred term (PT), with each patient being counted once at each summarization level. This summary will be repeated for treatment-emergent adverse events (TEAEs), AEs that lead to study drug discontinuation, by relationship to study drug, by intensity and for SAEs.

Laboratory test results, vital signs data, and electrocardiogram (ECG) data will be presented in tables summarizing absolute values and change from baseline by treatment group, with shift tables included as appropriate.

Virologic Efficacy Analysis

- Change from baseline in viral shedding as measured by RT-qPCR in NP or OP swab samples (Phase II, III);
- Time-weighted average change from baseline in viral shedding as measured by RT-qPCR in NP or OP swab samples (Phase II, III);
- Time to SARS-CoV-2 RNA negative in NP or OP swab samples as determined using RT-qPCR (Phase II, III).

Recommended Dose for Phase III Analysis

Based on the safety, virologic effect and clinical efficacy of SCTA01 15 mg/kg and 50 mg/kg groups in Phase II part, the dose that will continue to Phase III part will be determined by the effect in reduction of viral load compared to placebo at Day 8 and TTCI of each dose. The dose with greater virologic suppression effect will be selected with no harmful effect of clinical relevancy.

Phase III Clinical Efficacy Analysis

Primary endpoint is TTCI up to Day 29. TTCI is defined as the time (in days) from randomization to the first day on which a patient satisfies point 1, 2, or 3 on the 8-point ordinal scale and maintains a score ≤ 3 at least 48 hours (initial improvement) and maintains this up to Day 29 (sustained improvement).

All deaths up to Day 29 will be considered censored at Day 29. Any subjects that are lost to follow-up or terminated early prior to meeting primary endpoint will be censored at the day of their last observed assessment. Subjects who completed follow-up but do not meet primary endpoint will be censored at the day of their Day 29 visit. The subjects who take prohibited medications before Day 29 will be treated as treatment failures and will be censored at the time of medication use.

The primary analysis uses the stratified log rank test to compare treatment group to control group through Day 29 with respect to TTCI. Covariate factors may include country duration of symptoms prior to enrollment, comorbidity and best supportive treatment. Note that if some strata are too small, they might be combined together to allow statistical method work well.

For each group, the Kaplan-Meier estimate of TTCI survival function will be constructed. Hazard ratio of SCTA01 *vs.* placebo with two-sided 95% CI will be derived from the stratified Cox proportional hazard model. The number of patients with a TTCI event and number of patients censored for the TTCI analysis will be summarized by treatment group. Percentage of subjects in each category using the 8-point ordinal scale up to Day 29 will also be summarized by treatment group.

PK Analysis

Population pharmacokinetic analysis will be performed using NONMEM® (Version 7.4, Icon Inc, PA, USA)..

Immunogenicity Analysis

To collect the proportion of subjects with anti-SCTA01 antibody and to evaluate the change of anti-SCTA01 antibody in patient serum.

Non-Response to SCTA01 assessments

Number and proportion of non-responders having mutations in the gene encoding the SARS-CoV-2 Spike protein through Day 29. A non-responder is defined as a patient who does not achieve a reduction in viral shedding or persistence viral presence or have a rebound in viral shedding following suppression (Phase II, III).

Stopping Infusion Criteria: If a severe allergic reactions (including anaphylaxis) (Grade 3 or higher) is observed during infusion, the infusion will be immediately stopped and no further SCTA01/placebo treatment will be administered to the subject.

TABLE OF CONTENTS

PROTOCOL SYNOPSIS **2**

TABLE OF CONTENTS **10**

LIST OF TABLES **14**

LIST OF FIGURES **15**

SCHEMA **16**

ABBREVIATIONS AND DEFINITIONS OF TERMS **17**

1. STUDY OBJECTIVES AND ENDPOINTS **19**

1.1. Primary Objectives 19

1.2. Secondary Objectives 19

1.3. Exploratory Objectives 19

1.4. Primary Endpoints 19

1.5. Secondary Endpoints 19

1.6. Exploratory Endpoints 21

2. BACKGROUND **22**

2.1. Spike Protein and SARS-CoV-2 22

2.2. Convalescent Plasma in the Treatment of COVID-19 22

2.3. Potential Efficacy 22

2.4. Booster Neutralizing Antibodies against COVID-19 23

2.5. SCTA01 is a Neutralizing Antibody against SARS-CoV-2 23

2.6. Preclinical Pharmacokinetics 23

2.7. Preclinical Pharmacodynamics 24

2.8. Preclinical Toxicology 26

2.9. Clinical Experience and Dose Selection 29

2.10. Rationale 31

3. INVESTIGATIONAL PLAN **32**

3.1. Overall Study Design 32

3.2. Number of Subjects 33

3.3. Duration of Treatment 33

3.4. Dose Modification 33

3.5. Concomitant Therapy 35

3.5.1. Prior Treatments 35

3.5.2. Concomitant Therapy 35
3.6. Replacement of Subjects 36
3.7. End of Study Definition 36
4. SUBJECT ELIGIBILITY 37
4.1. Inclusion Criteria 37
4.2. Exclusion Criteria 37
4.3. Screen Failures 38
5. INVESTIGATIONAL PRODUCT MANAGEMENT AND ADMINISTRATION 39
5.1. Investigational Product 39
5.2. Administration 39
5.3. Management of the Investigational Product 39
5.3.1. Packaging and Labeling 39
5.3.2. Receipt 39
5.3.3. Storage 39
5.4. Accountability and Destruction 40
5.5. Return of Drugs 40
5.6. Randomization and Blinding 40
5.7. Other Considerations 41
6. STUDY INTERVENTION DISCONTINUATION 42
6.1. Individual Infusion Halting 42
6.2. Study Halting 42
6.3. Withdrawal from the Study 42
6.4. Lost to Follow-Up 42
7. STUDY PROCEDURES AND EVALUATIONS 44
7.1. Time and Events Schedule 44
7.2. Safety Assessments 50
7.2.1. Physical examinations 50
7.2.2. Vital signs 50
7.2.3. Electrocardiograms 50
7.2.4. SpO_2 51
7.2.5. Clinical Safety Laboratory Assessments 51
7.2.6. ADE Assessments 52

7.2.7. Pregnancy Testing 52
7.3. Clinical Efficacy Assessments 52
7.3.1. Time to Clinical Improvement 52
7.4. Viral Load Assessments 53
7.4.1. Viral Load Assessment in NP or OP Swab Samples 53
7.4.2. Viral Load Assessment in Blood Samples 53
7.5. Non-Response to SCTA01 Assessments 53
7.6. Pharmacokinetics Assessments 53
7.6.1. Intensive PK Sampling 54
7.6.2. Sparse PK Sampling 54
7.7. Immunogenicity Assessments 54
8. ADVERSE EVENTS 55
8.1. Monitoring, Recording, and Reporting of Adverse Events 55
8.2. Evaluation of Adverse Events 55
8.2.1. Seriousness 55
8.2.2. Severity/Intensity 56
8.2.3. Event Relationship to Study Drug 57
8.2.4. Duration 57
8.2.5. Action Taken 58
8.2.6. Outcome 58
8.2.7. Adverse Events of Special Interest 58
8.3. Abnormal Laboratory Values 58
8.4. Pregnancy 59
8.5. Reporting Serious Adverse Events 59
8.5.1. Safety Queries 60
8.6. Expedited Reporting of Adverse Events 60
9. STATISTICAL METHODS 61
9.1. General Principle 61
9.2. Statistical Hypotheses 61
9.3. Statistical Decision Rules 61
9.4. Sample Size 61
9.5. Baseline Descriptive Statistics 62

9.6. Safety Analysis 62
9.7. Efficacy Analysis 62
9.7.1. Primary Efficacy Analysis.... 62
9.7.2. Multiple Testing in Adaptive Seamless Design 63
9.7.3. Analysis of Secondary Endpoints.... 64
9.7.4. IDMC.... 64
9.7.5. Interim Analyses.... 64
9.7.6. Final Analysis.... 65
9.7.7. Sub-group Analyses 66
9.8. Group Sequential Multiplicity Adjustment 66
9.9. Handling of Missing Data 66
9.10. Pharmacokinetics and Immunogenicity Analysis 66
10. REGULATORY CONSIDERATIONS 67
10.1. Good Clinical Practice....67
10.2. Investigator Responsibilities 67
10.3. Subject Information and Informed Consent 67
10.4. Confidentiality....68
10.5. Protocol Amendment....68
10.6. Institutional Review Board/Independent Ethics Committee Review and Approval..68
10.7. Closure of Study....68
11. DATA HANDLING AND RECORD KEEPING 70
11.1. Data/Documents 70
11.2. Data Management....70
11.3. Inspection of Records....70
11.4. Retention of Records....70
12. QUALITY CONTROL AND QUALITY ASSURANCE 72
12.1. Study Monitoring 72
12.2. Audits and Inspections 72
13. PUBLICATION POLICY 74
14. REFERENCES 75
APPENDIX 1. INVESTIGATOR'S PROTOCOL AGREEMENT....78
APPENDIX 2. SPONSOR SIGNATURE....80

LIST OF TABLES

Table 1. Pharmacokinetic Parameters of SCTA01 from Non-compartmental Analysis 30

Table 2. Schedule for Screening Visit 45

Table 3. Schedule for Treatment Visit 46

Table 4. Schedule for Follow-up Visit 47

Table 5. Estimating Severity Grade for Parameters Not Identified in the Grading Table 56

Table 6. Grading for Acute Allergic Reaction and Cytokine Release Reaction 58

Table 7. Stopping Boundaries for Efficacy and Futility 65

LIST OF FIGURES

Figure 1. Design of the Phase II/III Trial 16
Figure 2. SCTA01 Inhibits Formation of Syncytium in Virus 24
Figure 3. Protective Effect against SARS-CoV-2 Infection in Two Mouse Models 25
Figure 4. Virus RNA Copies of Different Tissues after SARS-CoV2 Infection in Rhesus Monkey 26
Figure 5. Mean Concentration-time Curves of SCTA01 at 5, 15, 30 and 50 mg/kg Level from Interim Data of Phase Ia Trial in China 29

SCHEMA

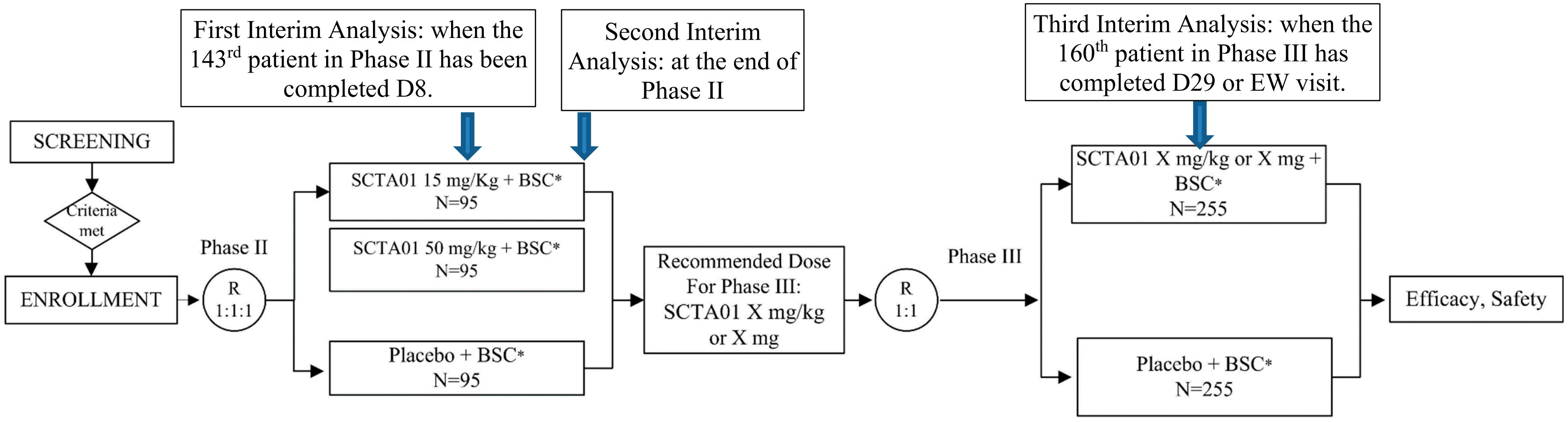

*BSC: Best Supportive Care

Figure 1. Design of the Phase II/III Trial

ABBREVIATIONS AND DEFINITIONS OF TERMS

Abbreviation	Definition
ACE2	Angiotensin Converting Enzyme 2
ADA	Anti-drug Antibodies
ADE	Antibody-dependent Enhancement
AE	Adverse Event
AESI	Adverse Event of Special Interest
ALT	Alanine-amino Transferase
AST	Aspartate Transaminase
AUC	Area Under the Curve
BSC	Best Supportive Care
C_{max}	Maximum Concentration
CCP	COVID-19 Convalescent Plasma
CL	Clearance
CrCL	Creatinine Clearance
COVID-19	Coronavirus Disease 2019
CRF	Case Report Form
CRO	Contract Research Organization
CSR	Clinical Study Report
CT	Computed Tomography
DAIDS	Division of AIDS
DLT	Dose Limited Toxicity
EC	Ethics Committee
ECG	Electrocardiogram
ECMO	Extracorporeal Membrane Oxygenation
EDC	Electronic Data Collection
eGFR	Estimated Glomerular Filtration Rate
EOS	End of Study
EUA	Emergency Use Authorization
FDA	Food and Drug Administration
FiO_2	Fraction of Inspired Oxygen
GCP	Good Clinical Practice
GLP	Good Laboratory Practice
IA	Interim Analysis
ICF	Informed Consent Form
ICH	International Conference on Harmonization

**Abbreviation**	**Definition**
ICSR	Individual Case Safety Report
IRB	Institutional Review Board
ICU	Intensive Care Unit
IWRS	Interactive Web Response System
ITT	Intention to Treat
IDMC	Independent Data Monitoring Committee
LDH	Lactate Dehydrogenase
NOAEL	No Observed Adverse Effect Level
NP	Nasopharyngeal
OP	Oropharyngeal
PaO_2	Arterial Partial Pressure of Oxygen
PT	Preferred Term
PKS	Pharmacokinetics Set
PKCS	PK Concentration Set
PKPS	PK Parameter Set
RBD	Receptor Binding Domain
RR	Rate Ratio
RT-qPCR	Quantitative Reverse Transcription Polymerase Chain Reaction
S	Spike
SAEs	Serious Adverse Events
SARS-CoV-2	Severe Acute Respiratory Syndrome Coronavirus 2
SCr	Serum Creatinine
SCT	Sinocelltech, Ltd.
SID	Subject Identification
SOFA	Sequential Organ Failure Assessment
SOP	Standard Operating Procedures
SUSAR	Suspected Unexpected Serious Adverse Reaction
SpO_2	Pulse Oxygen Saturation
SS	Safety Set
T_{max}	Time of Maximum Concentration
TTCI	Time to Clinical Improvement
$T_{1/2}$	Half-life Time
TEAEs	Treatment-emergent Adverse Events
Vd	Apparent Volume of Distribution

1. STUDY OBJECTIVES AND ENDPOINTS

1.1. Primary Objectives

- To evaluate the clinical efficacy of SCTA01 as assessed by time to clinical improvement (Phase II, III).

1.2. Secondary Objectives

- To evaluate the clinical efficacy of SCTA01 as assessed by clinical severity, hospitalization, mortality (Phase II, III);
- To evaluate the safety of SCTA01 (Phase II, III);
- To evaluate the virologic efficacy of SCTA01 (Phase II, III);
- To evaluate the pharmacokinetics of SCTA01 (Phase II);
- To evaluate the immunogenicity of SCTA01 (Phase II, III).

1.3. Exploratory Objectives

- To explore the correlation between the baseline serological antibody level and therapeutic effect (Phase II, III);
- To evaluate the virologic efficacy of SCTA01 as assessed by quantitative SARS-CoV-2 virus in patient's blood (Phase II, III).

1.4. Primary Endpoints

- Time to clinical improvement (TTCI) up to Day 29. TTCI is defined as the time (in days) from randomization to the first day on which a patient satisfies Point 1, 2, or 3 on the 8-point ordinal scale[1] and maintains a score ≤ 3 at least 48 hours (initial improvement) and maintains this up to Day 29 (sustained improvement) (Phase II, III).

1.5. Secondary Endpoints

- Clinical efficacy of SCTA01:
  - Proportion of patients with initial clinical improvement at Day 29 (Phase II, III);
  - Proportion of patients with sustained clinical improvement at Day 29 (Phase II, III);
  - Time to initial clinical improvement up to Day 29 (Phase II, III);
  - Time to discharge from hospital, followed by being alive and home for 14 consecutive days prior to Day 90 (Phase II, III);
  - Proportion of patients discharged from hospital, followed by being alive and home for 14 consecutive days prior to Day 90 (Phase II, III);
  - All-cause mortality up to Day 29, record date and cause of death (if applicable) (Phase II, III);

- Percentage of subjects in each category on the 8-point ordinal scale (time frame: Days 3, 5, 8, 11, 15, 22, and 29) (Phase II, III);
- Proportion of patients alive and free of respiratory failure up to Day 29 (Phase II, III);
- Proportion of patients who are discharged from acute care up to Day 29 (Phase II, III);
- Days of supplemental oxygen (if applicable) up to Day 29 (Phase II, III);
- Days of non-invasive ventilation/high flow oxygen (if applicable) up to Day 29 (Phase II, III);
- Days of invasive mechanical ventilation/extracorporeal membrane oxygenation (ECMO) (if applicable) up to Day 29 (Phase II, III);
- Time to hospital discharge up to Day 29 (Phase II, III);
- Number and proportion of non-responders having mutations in the gene encoding the SARS-CoV-2 S protein through Day 29. A non-responder is defined as a patient who does not achieve a reduction in viral shedding or persistence viral presence or have a rebound in viral shedding following suppression (Phase II, III).

- Safety of SCTA01:
  - Cumulative incidence of serious adverse events (SAEs) (Phase II, III);
  - Cumulative incidence of Grade 1, 2, 3 and 4 clinical and/or laboratory adverse events (AEs) (Phase II, III);
  - Discontinuation or temporary suspension of infusions (for any reason) (Phase II, III);
  - Chemistries, hematology, urinalysis, coagulation function, IL-6, ferritin, procalcitonin, thrombin time, D-dimer (Phase II, III);
  - Number and proportion of patients who are suspected to experience antibody-dependent enhancement (ADE) (Phase II, III).
- Virologic efficacy of SCTA01:
  - Change from baseline in viral shedding as measured by quantitative reverse transcription polymerase chain reaction (RT-qPCR) in NP or OP swab samples (Phase II, III);
  - Time-weighted average change from baseline in viral shedding as measured by RT-qPCR in NP or OP swab samples (Phase II, III);
  - Time to SARS-CoV-2 RNA negative in NP or OP swab samples as determined by RT-qPCR (Phase II, III).
- Pharmacokinetics (PK) of SCTA01:
  - Pharmacokinetic characteristics in severe COVID-19 patients (Phase II).
- Immunogenicity of SCTA01:

- Immunogenicity assessment include the number and percentage of subjects who develop detectable ADA (Phase II, III).

1.6. Exploratory Endpoints

- The correlation between the baseline serological antibody level and therapeutic effect (Phase II, III);
- Quantitative SARS-CoV-2 virus in patient's blood (Phase II, III).

2. BACKGROUND

2.1. Spike Protein and SARS-CoV-2

The pandemic of SARS-CoV-2 has spread globally, and causing massive death and economic hardship. By 17 Dec 2020, there were 74,226,214 cases confirmed infection and 1,649,032 deaths worldwide, and in the United States, there were 16,979,762 cases confirmed infection, and 307,501 deaths[2]. Although Remdesivir® is approved for COVID-19 in the United States[3], Itolizumab is approved for COVID-19 in India and Cuba[4], and FDA issued EUAs for SARS-CoV-2 neutralizing mAbs of Lily's LY-CoV555[5] and Regeneron's Casirivimab and Imdevimab[6] for the treatment of mild to moderate COVID-19, but there are still huge unmet clinical needs for treating COVID-19, especially for treating severe and critical COVID-19 patients.

The spike (S) glycoprotein of SARS-CoV-2 is a critical target for diagnosis and treatment. The S protein binds to angiotensin-converting enzyme 2 (ACE2), and helps virus entry into host cells. The SARS-CoV-2 has a 10 ~ 20 fold stronger affinity than SARS-CoVs' binding to ACE2, which account for its high infectivity[7].

2.2. Convalescent Plasma in the Treatment of COVID-19

The infusion of convalescent plasma is not a novel therapeutic method. It has been evaluated against a variety of viral diseases, which includes severe acute respiratory syndrome (SARS), influenza H1N1 and Ebola, with potential benefits[8-10]. In this COVID-19 epidemic, it has also been tested and made some progress.

2.3. Potential Efficacy

The convalescent plasma therapy showed potential therapeutic effect against COVID-19 in China. There were five critically ill patients with SARS-CoV-2 infection who were transfused with convalescent plasma. Their clinical conditions improved, including normalization of body temperature, decreased Sequential Organ Failure Assessment (SOFA) score, gradually decreasing and ultimately negative viral loads, and increased titers of specific neutralizing antibodies against SARS-CoV-2[7]. Convalescent plasma therapy also showed a beneficial effect in two studies, which consisted of 10 and 6 critical patients, respectively[12, 13]. All the patients showed relief of clinical symptoms, improved laboratory and pulmonary radiology results to some extent. There were also another 4 cases with severe SARS-CoV-2 infection who were reported to have completely recovered following convalescent plasma infusion[14]. The transfusion of convalescent plasma also shows clinical benefit in six patients with critical SARS-CoV-2 infection in Mexico[15]. Moreover, in the US, Eric Salazar et al. conducted a clinical trial involving 25 critical patients who received the treatment of convalescent plasma[16]. 9 patients (36%) showed clinical improvement when evaluated by a modified World Health Organization 6-point ordinal scale on the 7th day after transfusion. 19 cases (76%) were improved or discharged on the 14th day. No specific adverse events with convalescent plasma treatment were observed. Salazar and colleagues evaluated 136 individuals with COVID-19 who received convalescent plasma and who had data available for 28-day outcomes. These individuals were well-matched to 251 non-transfused control COVID-19 patients. The analysis that these investigators performed indicated that there was a statistically significant reduction in mortality at 28 days that was most notable in the 112 patients transfused within 72 hours of admission with plasma having a high anti-spike protein receptor binding domain

titer of ≥1:1350[17]. These studies described above indicate that convalescent plasma therapy is well tolerated, beneficial for symptom relief and disease control in COVID-19 patients. So on August 23, 2020, the U.S. Food and Drug Administration (FDA) issued an Emergency Use Authorization (EUA) for COVID-19 Convalescent Plasma for the treatment of hospitalized patients with COVID-19[18]. But limitations of these studies include non-randomized, open label, retrospective, etc. So adequate and well-controlled randomized trials remain nonetheless necessary for a definitive demonstration of COVID-19 convalescent plasma (CCP) efficacy and to determine the optimal product attributes and the appropriate patient populations for its use.

2.4. Booster Neutralizing Antibodies against COVID-19

Convalescent plasma is collected from people who have completely recovered from COVID-19. Because the limited number of patients who can provide convalescent plasma, there is a limit on supply. In addition to neutralizing antibodies, there are anti-N protein antibodies and other antibodies in convalescent plasma. So the concentration of neutralizing antibodies may be low due to the presence of non-neutralizing antibodies and the therapeutic effect could be limited and inconsistent. Therefore, it is urgent to develop a safe and effective neutralizing antibody for the treatment of COVID-19.

2.5. SCTA01 is a Neutralizing Antibody against SARS-CoV-2

SCTA01 is a potential therapeutic biologic independently developed by Sinocelltech, Ltd. (SCT), a biotech company based in Beijing, China. It is a recombinant humanized anti-receptor binding domain (RBD) of the SARS-CoV-2 spike (S) protein monoclonal antibody. The antibody is an IgG1 with the Fab region having high specificity and affinity for the RBD and S protein, blocking their binding to a human receptor (specifically ACE2), and the Fc region having specific sequence modifications to significantly reduce its ability to interact with human Fc receptors and complement, thereby significantly reducing potential side effects, such as antibody-dependent enhancement (ADE) of virus infection. Consequently, it is expected that SCTA01 would likely be better in the safety profile in comparison with other anti-RBD or S protein monoclonal antibodies without such binding reduction to Fc receptors and complement.

2.6. Preclinical Pharmacokinetics

SCTA01 is a recombinant humanized anti-SARS-CoV-2 monoclonal antibody that targets the RBD of the S protein of SARS-CoV-2. Therefore, there is no pharmacologically relevant animal species that can be used to conduct pharmacokinetic studies. As a result, the preliminary pharmacokinetic study was conducted in C57BL/6 mice, and the dynamic changes of the drug in the body were studied through tail vein injection to obtain the relevant PK parameters. Moreover, SCTA01 was administered via intravenous infusion to rhesus monkeys with a single dose at 150 and 500 mg/kg, which can obtain the PK characteristics of SCTA01 in non-human primates.

Six C57BL/6 mice were selected, half male and half female. The dose for a single administration was 5 mg/kg, and the dose volume was 10 mL/kg. The pharmacokinetic results showed that SCTA01 administered to mice at a single dose of 5 mg/kg through the tail vein had normal PK characteristics. The serum drug concentration gradually decreased, the average C_{max} and AUC_{last} were 122.81 μg/mL and 16530.33 h·μg/mL, respectively. The average $t_{1/2}$ was 260.21 h, and the CL was 0.25 mL/h/kg.

Toxicokinetics (TK) was combined with the extended single-dose toxicity study in rhesus monkey. The serum concentration of SCTA01 in rhesus monkeys was analyzed using a validated ELISA method. After the administration of 150 and 500 mg/kg of SCTA01 to rhesus monkeys, the serum drug concentration correlated positively with the dose, and no significant gender difference was observed.

The ratio of the two SCTA01 dose groups is 1:3.33, the C_{max} was 3.98 mg/mL and 12.3 mg/mL (ratio 1:3.09), respectively, and the AUC_{last} was 117 h·mg/mL and 375 h·mg/mL (ratio was 1:3.21), respectively. The systemic drug exposure was basically dose-dependent. Moreover, the pharmacokinetic parameters (0~336 h) of rhesus monkeys in the 500 mg/mL high-dose group show that the average half-life ($t_{1/2}$) was 239 ± 54.3 h, the average C_{max} and AUC_{last} were 12.8 ± 0.826 mg/mL and 1460 ± 153 h·mg/mL, respectively, indicating slow drug metabolism and high systemic drug exposure.

2.7. Preclinical Pharmacodynamics

In vitro studies conducted by SCT have shown that SCTA01 can bind to the SARS-CoV-2 RBD protein and effectively block its binding to ACE2 receptors, and has a strong neutralization effect. Neutralization tests with both the SARS-CoV-2 pseudo viruses and the natural viruses confirmed the ability of SCTA01 to effectively neutralize the viruses. The binding affinity of SCTA01 under lower pH conditions (i.e., pH 5.0, pH 6.0) was similar to that under neutral pH, indicating that the antibody-virus complex can maintain a stable state, thereby capable of reducing the binding of the virus to the ACE2 receptor. Therefore, under the acidic conditions of cells, SCTA01 can inhibit the formation of virus-induced syncytia (Figure 2). SCTA01 can also effectively bind and neutralize the world pandemic SARS-CoV-2 strain (D614G mutant strain) and all known global RBD mutant strains. Overall, these results showed that SCTA01 can bind and neutralize the SARS-CoV2 virus and be a good candidate for the effective therapeutic and passive prevention of COVID-19.

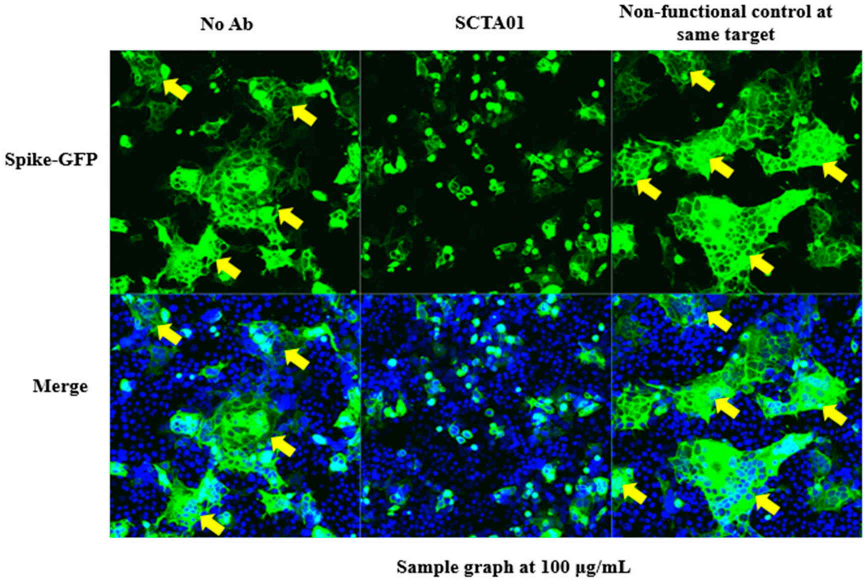

Figure 2. SCTA01 Inhibits Formation of Syncytium in Virus

Antibody-virus complexes captured by immune cells (such as pulmonary macrophages), may cause antibody-dependent enhancement (ADE) of viral infections. The constant region of the SCTA01 heavy chain has a LALA mutation, which is shown experimentally to reduce the ability to bind to both Fc receptors and C1q complement. Furthermore, SCTA01 showed clearly both the reduction in inducing ADE in the *in vitro* model stransfected cell lines and superiority over other anti-RBD or S protein monoclonal antibodies without the LALA mutation. As a result, the potential of ADE caused clinically by this antibody therapy can be significantly reduced or avoided.

SCTA01 showed protective effect against SARS-CoV-2 infection in two mouse models (Figure 3), the SARS-CoV-2 challenged human ACE2 transgenic mice and the mouse-adapted SARS-CoV-2 strain challenged BALB/c mice. Experiments with SCTA01 in both prophylaxis and treatment showed that it can reduce viral loads in lung and reduce lung inflammation. The pathological results showed that SCTA01 reduced lung inflammation, and no enhanced inflammation (i.e. no ADE) was detected compared to the untreated model group.

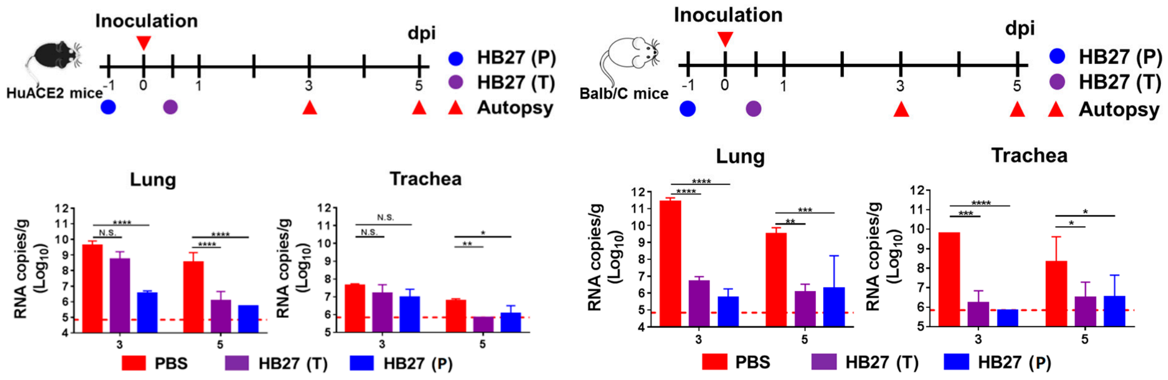

Figure 3. Protective Effect against SARS-CoV-2 Infection in Two Mouse Models

Furthermore, in a SARS-CoV-2-challenged rhesus macaques model, SCTA01 50 mg/kg intravenously 4 h after challenge showed significant virus-clearance effect (the virus loads of lung and upper respiratory tract tissues were undetectable upon treatment with SCTA01 compared with dilution buffer control animals) in the infected monkey model (Figure 4). The pathological results showed that SCTA01 also reduced viral loads of throat swabs, nasal swabs and anal swabs of rhesus macaques after SARS-CoV-2 infection. The pathological results showed that SCTA01 reduced lung inflammation, and no clear ADE phenomenon had been observed.

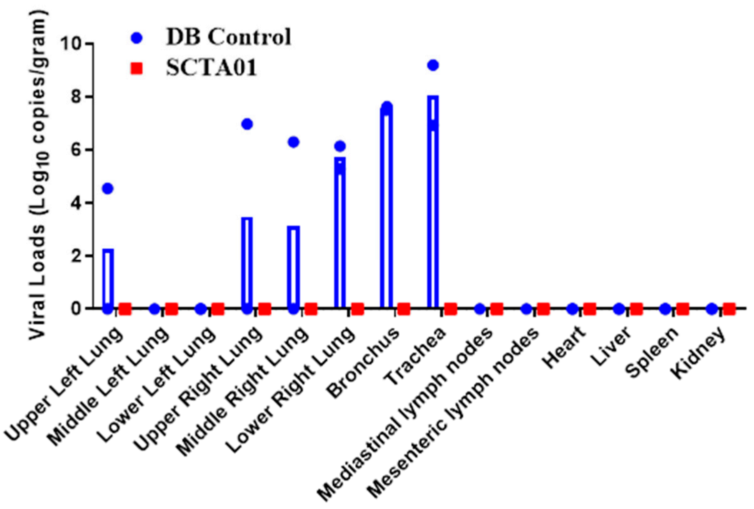

Figure 4. Virus RNA Copies of Different Tissues after SARS-CoV2 Infection in Rhesus Monkey

2.8. Preclinical Toxicology

Complete toxicology investigation was conducted for SCTA01 according to the latest ICH regulatory guidance to support a single-dose application in the clinical trial. All of the toxicity studies were completed in strict compliance with the Good Laboratory Practice (GLP). Toxicology studies included the assessments of seven different aspects, such as tissue cross-reactivity, safety pharmacology, extended single-dose toxicology, local irritation, immunogenicity, immunotoxicity and *in vitro* hemolysis test.

1) Extended single-dose toxicity study

The objective of this study was to evaluate the toxicity, target organ of toxicity and toxicokinetic profile of SCTA01 after a single dose administration in rhesus monkeys via intravenous infusion with a 2-week observation period, to determine the maximum tolerated dose, and to provide reference for dose design and toxic side effects monitoring in clinical trials.

The study design was as follows: vehicle control group (5 animals/sex/group), 150 mg/kg group (3 animals/sex/group) and 500 mg/kg group (5 animals/sex/group). On Day 1, a single dose was given to monkeys by intravenous infusion. The dose volume was 20 mL/kg, and the administration rate was about 0.5 mL/kg/min.

Analyzed items included: clinical observation, weight, food consumption, body temperature, electrocardiogram, respiration, blood pressure, ophthalmoscopic examinations, hematology, coagulation, clinical chemistry, urinalysis, T lymphocyte subsets ($CD3^+$, $CD3^+$ $CD4^+$, $CD3^+$ $CD8^+$and $CD3^+$ $CD4^+$ / $CD3^+CD8^+$), cytokines (IL-2, IL-4, IL-5, IL-6, TNF-α, IFN-γ), serum complement determination (C3 and C4), immunoglobulin (IgA, IgM and IgG), C-reactive protein, serum anti-drug antibodies and toxicokinetics. On the third day after administration, the first 3 animals in all groups are euthanized, and the remaining 2 animals/sex/group were euthanized at the end of the observation period of 2 weeks after administration. Complete autopsy was conducted on all animals. In addition, organ weight, gross and microscopic observation were also performed.

Throughout the study, no mortality was recorded in any group. No changes related to the test articles were noted in clinical observation, body weight, food consumption, electrocardiogram, blood pressure, respiratory function, ophthalmoscopic examinations, hematology, clinical chemistry, urinalysis, T lymphocyte subsets, cytokines, complement, immunoglobulin and C-reactive protein, organ weight, gross and microscopic observation in any group throughout the study.

The serum concentration of SCTA01 in rhesus monkeys was analyzed using a validated ELISA method. After the administration of 150 and 500 mg/kg of SCTA01, the serum drug concentration was positively correlated with the dose, and no significant gender difference was observed.

In summary, this study showed that when SCTA01 was administered to rhesus monkeys by intravenous injection in single doses of 150 and 500 mg/kg, no obvious toxicity was observed, and the maximum tolerated dose (MTD) was considered to be 500 mg/kg, which could also be thought as No Observed Adverse Effect Level (NOAEL) due to the single injection in clinical use.

2) Immunogenicity

Immunogenicity testing was conducted in the extended single-dose toxicity study in rhesus monkeys by testing for the presence of anti-drug antibodies and their potential impact on efficacy and toxicity. No anti-drug antibody was detected in any group at all of the time points, and there is no immunogenicity in rhesus monkey with this single-dose administration of SCTA01. The results showed that SCTA01, when given to rhesus monkeys by intravenous infusion at a single dose of 150 and 500 mg/kg, did not induce ADA.

3) Immunotoxicity

The immunotoxicity of SCTA01 was assessed in the extended single-dose toxicity study in rhesus monkeys.

The results showed that there was no significant effect of SCTA01 on the counts of white blood cells (WBC), WBC subtypes, T lymphocyte subsets, immunoglobulin (IgA and IgM), complement (C3 and C4), C-reactive protein, gross and microscopic observations of immune organs (thymus, spleen, lymph nodes), organ weight or ratios.

On Day 3, in the test group at a dose of 500 mg/kg, both male and female monkeys had a statistically significant increase in IgG. Considering that the test article was an IgG monoclonal antibody and the amount injected relative to the total monkey blood volume, the observed elevated IgG was considered to have no toxicological significance.

In short, there was no immunotoxicity observed with the test article.

4) Local tolerance

Local tolerance study was conducted with the extended single-dose toxicity study in rhesus monkeys to evaluate the effect of SCTA01 on local irritation at the infusion site. During the experiment, local reactions were assessed on dosing day (prior to dosing) and Day 2, for visible and palpable changes. After the end of the administration and recovery period, the animals were euthanized, the macroscopic and microscopic observations of the injection site were conducted.

The results showed that no abnormal changes were found in the injection site of any group, and no changes related to the test article were observed in the gross and microscopic observations during the entire study.

In summary, SCTA01 given to rhesus monkeys by intravenous infusion, with a single dose of 150 and 500 mg/kg, indicated that no irritation of the injection sites occurred.

5) Safety pharmacology

This study was combined with an extended single-dose toxicity study in rhesus monkeys to reveal any functional effects on the major physiological systems (e.g., cardiovascular, respiratory, and central nervous systems). The results showed that there were no abnormal changes in the mental state and behavior of all monkeys. There were no changes in the heart rate, ECG wave, PR interval, QRS time limit, QT interval and QTc values at any time point in any group. During the entire study, no abnormal changes in the blood pressure (mean arterial pressure, diastolic blood pressure, and systolic blood pressure) were observed. The tidal volume (TV, mL) of female monkeys administered at a dose of 500 mg/kg of SCTA01 showed a statistically significant decrease prior to dosing and approximately 2 to 4 hours after administration on the first day. These changes were not considered to be related to the test article, because the observed tidal volume value occurred prior or close to the dosing.

In summary, during the entire study, no changes in the clinical parameters in any group related to the test samples were observed. Compared with the vehicle control group, no biologically significant changes in body temperature, blood pressure, pulse rate or ECG parameters were found in animals administered with 150 and 500 mg/kg of SCTA01. After intravenous injection of SCTA01 of a single dose of 150 and 500 mg/kg in rhesus monkeys, there was no significant effect on the rhesus monkey's nervous system, cardiovascular system and respiratory system.

6) Tissue cross reactivity studies of normal human and rhesus monkey

Streptavidin-biotin immunohistochemical method was used to evaluate the tissue cross reactivity of SCTA01 monoclonal antibody in frozen normal human and rhesus monkey tissues. Each tissue came from three different donors. The test consisted of two test item groups with different concentrations (SCTA01 injection/biotin, 1.0 μg/mL and 10.0 μg/mL), an isotype control group (human IgG1, κ isotype control biotin, 10.0 μg/mL) and a negative control group (phosphate buffered saline containing Tween 20).

As a result, a brown positive staining signal was observed in the cell membrane/cytoplasm of the positive control slide in all test groups. No positive staining was observed in the slides of 34 types of frozen normal human tissues or frozen normal rhesus monkey tissues.

In conclusion, under the conditions of this study, SCTA01 did not specifically bind to frozen normal human and rhesus monkey tissues.

7) *In vitro* hemolysis test

This *in vitro* study evaluated the hemolysis or aggregation effect of SCTA01 using human red blood cells. A positive control group (sterile water for injection), a negative control group (sodium chloride injection) and 5 tubes of SCTA01 (0.5, 0.4, 0.3, 0.2, 0.1 mL/tubes, 25 mg/mL SCTA01) were used. The results showed that complete hemolysis occurred in the positive control tube, but no hemolysis or aggregation was observed in the test product and the negative control tube.

Therefore, under the conditions of this study, 25 mg/mL of SCTA01 had no hemolytic or aggregation effect on human RBCs.

2.9. Clinical Experience and Dose Selection

A Phase Ia study: the first-in-human study (SCTA01-X101, NCT number: NCT04483375) is being conducted in China in healthy subjects to assess the safety and tolerability of SCTA01, as well as to determine its PK and immunogenicity profile.

The available data as of 21 Sep 2020 of the Phase Ia study showed no observed dose limiting toxicities (DLT) after the completion of all four dose groups (each dose group consisted of placebo and either 5 or 15 or 30 or 50 mg/kg) in 33 healthy volunteers. The human experience of SCTA01 indicated that SCTA01 is safe and well-tolerated at all four tested dosing levels. There have been no serious adverse events (SAEs) or deaths reported, and all of adverse events have been transient and mild or moderate in intensity observed while treatment assignment has remained blinded.

The available preliminary PK data for the four doses (5, 15, 30 and 50 mg/kg) showed normal dose-dependent clearance, as expected for an IgG single-dose PK in human when the IgG has no *in vivo* target. The concentration-time curve of each group is shown in Figure 5. The PK parameters of each group is shown in the Table 1.

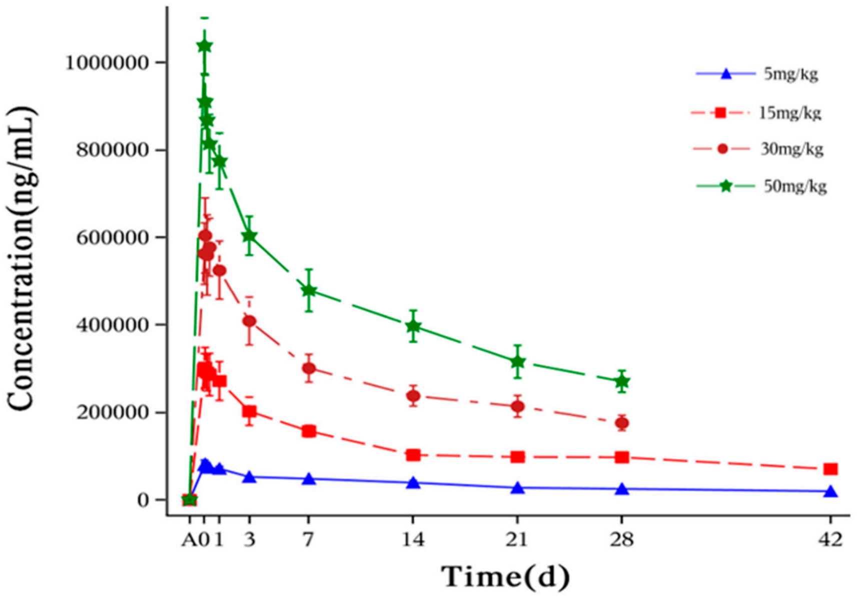

Figure 5. Mean Concentration-time Curves of SCTA01 at 5, 15, 30 and 50 mg/kg Level from Interim Data of Phase Ia Trial in China

Table 1. Pharmacokinetic Parameters of SCTA01 from Non-compartmental Analysis

	$T_{1/2}$ (h)	T_{max} (h)	C_{max} (ng/mL)	C_{last} (ng/mL)	$AUC_{0\text{-}Day28}$ (h*ng/mL)	$AUC_{0\text{-}t}$ (h*ng/mL)	$AUC_{0\text{-}\infty}$ (h*ng/mL)	Vd (L)	C (L/h)
5 mg/kg (n=3)	1040 (15.7%)	1.32 (0.32 ~ 1.32)	84800 (7.7%)	19900 (14.6%)	26900000 (8.5%)	34500000 (9.6%)	64400000 (14.6%)	7.06 (9.8%)	0.00475 (12.4%)
15 mg/kg (n=6)	821 (29.2%)	1.92 (0.92 ~ 8.92)	307000 (15.6)	70300 (11.7%)	89900000 (9.3%)	118000000 (9.0%)	201000000 (14.8%)	5.92 (19.6%)	0.00516 (14.8%)
30 mg/kg (n=8)	574 (25.1%)	2.83 (1.83 ~ 9.83)	607000 (13.8)	176000 (9.8%)	185000000 (9.5%)	185000000 (9.5%)	331000000 (14.5%)	4.57 (23.2%)	0.00558 (11.9%)
50 mg/kg (n=8)	625 (19.9%)	3.16 (3.15 ~ 3.17)	1040000 (6.1%)	270000 (9.1%)	286000000 (8.2%)	287000000 (8.2%)	532000000 (12.8%)	5.12 (13.2%)	0.00581 (18.5%)

Data are presented as the arithmetic mean (coefficient of variation), T_{max} data is median (range).

$T_{1/2}$ = half-life. T_{max} = time of maximum concentration. C_{max} = maximum concentration. C_{last} = concentration at last sample time point. $AUC_{0\text{-}t}$ = area under the concentration-time curve from time of dose to last sample time point. $AUC_{0\text{-}Day28}$ = area under the concentration-time curve from time of dose to Day 28 time point. $AUC_{0\text{-}\infty}$ = area under the concentration time curve from 0 hour to infinity.

The data is from the interim report of SCTA01-X101. The cutoff time is 21th Sept, 2020. The last time point of 5 mg/kg and 15 mg/kg groups were Day 42, 30 mg/kg and 50 mg/kg groups were Day 28.

Detailed safety and PK data of all groups are available in the Investigator Brochure (IB).

Since there is no specific binding target in animals and healthy subjects who have not been infected by SARS-CoV-2, the No Observed Adverse Effect Level (NOAEL) in monkey is 500 mg/kg. If calculated by the area normalization method of body surface[19], the human equivalent dose is 200 mg/kg. When the safety factor is set to equal 10, the initial dose will be 20 mg/kg. Based on the results of the *in vivo* pharmacodynamic study and the fact that there is no toxicity observed, as well as the initial doses of other similar antibody products for Phase I that have been reported, SCT started the initial human dose at 5 mg/kg, and increased in the escalating steps to 15 mg/kg, 30 mg/kg and 50 mg/kg in Phase Ia study in China. SCTA01 20 mg/kg can significantly reduce the viral load of lung and bronchus in humanized ACE2 mouse model for SARS-CoV-2 infection. The pathological results of mouse model showed that the infected tissue was mainly limited at the lungs with mild lung interstitial lesions, the same as those in mild/moderate infection in COVID-19 patients[20]. However, SARS-CoV-2 can be detected in multiple organs in severe patients, including the lungs, pharynx, heart, liver, brain, and kidneys, which indicate its broad organ tropism[21, 22]. The mean viral load of severe cases was around 10~60 times higher than that of mild cases, suggesting that higher viral loads might be associated with severe clinical outcomes and high risk of death[23-26]. Therefore, clinical dosage should be increased compared with the mouse model. SCTA01 50 mg/kg has shown significant antiviral effect in the pre-clinical study of monkey model of SARS-CoV-2 infection with excellent safety profile. So the 50 mg/kg will be chosen as the higher dose of this study.

Recently, the data published by Lilly and Regeneron showed that the efficacy may not be dose dependent[27,28], which indicated that lower dose maybe also have similar efficacy with better safety profile compared to higher dose. Therefore, SCT selects 15 mg/kg as the lower dose, which corresponds to approximately 20 mg/kg.

Based on the preclinical study data, the safety and PK data of Phase Ia and the high viral loads observed in severe COVID-19 subjects, SCT proposes SCTA01 15 mg/kg and 50 mg/kg as doses of Phase II part, and the dose of Phase III part will be determined by Phase II part results.

2.10.Rationale

The preclinical and clinical data of SCTA01 merit a Phase II part with the SCTA01 15 mg/kg, SCTA01 50 mg/kg and placebo to evaluate the safety and efficacy of SCTA01 in severe patients with COVID-19 and a Phase III part with the single dose of SCTA01 (X mg/kg or X mg) selected through Phase II results to evaluate the safety and efficacy of SCTA01 in patients with severe COVID-19.

3. INVESTIGATIONAL PLAN

3.1. Overall Study Design

The study is a multicenter, adaptive, randomized, double-blinded and placebo-controlled Phase II/III trial, and will be conducted at selected investigational sites globally.

Phase II part will evaluate the efficacy, safety, and PK of SCTA01 15 mg/kg, 50 mg/kg in patients with severe COVID-19. The primary endpoint will evaluate TTCI in study group 1 [SCTA01 15 mg/kg + best supportive care (BSC)], study group 2 (SCTA01 50 mg/kg + BSC) and control group (placebo + BSC) up to Day 29. The secondary endpoint will evaluate the virologic efficacy in study group 1 (SCTA01 15 mg/kg +BSC), study group 2 (SCTA01 50 mg/kg + BSC) and the control group (placebo + BSC) from baseline to Day 8, and then determine the recommended dose for Phase III part.

In the Phase II part of the study, 285 subjects will be randomized by 1:1:1 ratio to 15 mg/kg group, 50 mg/kg group and placebo group, with 95 subjects in each group.

At the end of Phase II part, enrollment will be paused and an interim analysis for dose selection will be performed after the 285th subject has been observed for TTCI. An IDMC will assess the benefit and risk to decide the dose for Phase III part of the part.

Enrollment will be resumed and the study continue into Phase III part after the IDMC has completed the evaluation and recommended the dose for Phase III part.

The Phase III part will evaluate the efficacy, safety, and immunogenicity of SCTA01 at the dosage recommended by the IDMC (X mg/kg or X mg) in severe patients with COVID-19. The primary endpoint will evaluate TTCI (8-point ordinal scale) in study group (SCTA01 X mg/kg or X mg + BSC) and control group (placebo + BSC) up to Day 29.

Approximately 510 subjects in Phase III part will be randomized by 1:1 ratio to SCTA01 (X mg/kg or X mg) and placebo, with 255 subjects in each group. The IDMC will be used to monitor safety and efficacy information.

Total number of subjects in the study is about 795.

Interim analysis (IA): There will be three un-blinded interim analyses when 143rd patient in Phase II has completed Day 8 visit, at the end of Phase II and when the 160th patient in Phase III has completed Day 29 or Early Withdraw visit.

- The 1st interim analysis is for safety evaluation. Only safety data will be reviewed at the 1st interim analysis. The IDMC may request to review efficacy data if early data warrants. Decision s from the IDMC will take the nature of premature data into account unless prevailing data suggest conclusive outcome of the trial. Early safety signal will guide patient enrollment to maximize the effectiveness of treating this patient population with SCTA01.
- The 2nd interim analysis is for dose selection: the analysis will be performed at the end of Phase II when the 285th patient completed Day 29 or Early Withdraw visit. The dose selected to continue to the Phase III part will be the dose with the greater effect in reduction of viral load compared to placebo at Day 8. Time to clinical improvement of each dose will be evaluated and the RR threshold of 1.15 will be used as a futility boundary at the end of Phase II. The futility boundary is non-binding, and the IDMC may recommend study continuation

even if the futility bound is met on TTCI. If any safety signal is observed for any dose, the IDMC will weigh this information against the efficacy results in selecting the dose for Phase III.

- The 3rd interim analysis is the penultimate analysis that allows for stopping for superior efficacy. It is planned when 160 patients in Phase III (approximately 31% information time of Phase III part, which will have approximately 128 TTCI events) have been documented in the ITT population. If the interim analysis is performed at 31% of information time of Phase III, based on Pocock type boundary of α-spending function, a 1-sided *p*-value less than 0.01068 using inverse normal method to combine *p*-value from Phase II part and Phase III part will need to be observed to declare statistical significance. A futility boundary of an observed RR of 1.22 will be used to declare failure of the study when TTCI events from Phase II and Phase III part combined are exactly 280, which is equivalent to an observed one-sided p-value of 0.05.

Note: The critical values of nominal *p*-value and hazard ratio that will be used to declare statistical significance and/or futility at the interim analysis will be calculated based on the actual number of the TTCI events documented at the time the interim analysis.

The interim analyses will be performed by an independent statistician external to the study team. The results of the interim analyses will be presented to the IDMC by the independent statistician. The IDMC will provide the Sponsor with a recommendation to continue the trial as planned, to have an interruption, to make a protocol amendment, or to terminate the trial. If the interim analysis demonstrates significant shortening of TTCI in the SCTA01 group compared to the control group, IDMC could recommend to terminate and un-blind the study, and the study could be announced as completed.

3.2. Number of Subjects

Up to 795 patients will be randomized for this Phase II/III study.

3.3. Duration of Treatment

Single dose of drug.

3.4. Premedication for Infusion

Premedication for infusions is not planned. However, if the participant has a medical history suggesting a potential benefit from premedication, the study investigator (s) should determine the appropriate premedication. The investigators and sponsor may decide to use premedication if the frequency of infusion reactions among participants warrants it.

If minor infusion reactions are observed, administration of acetaminophen, antihistamines and/or other appropriately indicated medications may be given prior to the start of infusions for subsequent participants.

The decision to implement premedication for infusions in subsequent participants will be made by the investigator and sponsor and recorded in the study documentation.

Any premedication given will be documented as a concomitant therapy.

3.5. Dose Modification

No dose modification is needed.

All subjects should be monitored closely for infusion-related reactions and anaphylaxis for at least 2 hours after the completion of the infusion. Emergency equipment and medication for the treatment of infusion-related reactions and anaphylaxis must be available for immediate use during the infusion and the post-infusion monitoring period if required for treatment.

Sites should have appropriately trained medical staff to recognize and treat infusion-related reaction and anaphylaxis. It is recommended that participants who experience an infusion-related reaction or anaphylaxis can be treated per the local standard of care.

The infusion can be reduced to 50% of the infusion rate / interrupted then be restarted at 50% of the original rate if any of the following adverse events are observed:

- Sustained/severe cough
- Rigors/chills
- Rash, pruritus (itching)
- Urticaria (hives, welts, wheals)
- Diaphoresis (sweating)
- Hypotension
- Dyspnea (shortness of breath)
- Vomiting
- Flushing

If investigators feel there is a medical need for treatment or discontinuation of the infusion other than described above, they should use clinical judgment to provide the appropriate response according to standard clinical practice.

The infusion should be terminated and not restarted if any of the following adverse events occur:

- Anaphylaxis*
- Laryngeal/pharyngeal edema
- Severe bronchospasm
- Seizure
- Severe hypotension
- Other neurological symptoms (confusion, loss of consciousness, paresthesia, paralysis, etc)
- Any other symptom or sign that, in the opinion of the investigator, warrants termination of the

intravenous infusion

*Consider anaphylaxis if the following is observed [27]: acute onset of an illness (minutes to several hours) with involvement of the skin, mucosal tissue, or both (eg, generalized hives, pruritus or flushing, swollen lips-tongue-uvula) and **at least one of the following:**

• Respiratory compromise (eg, dyspnea, wheeze-bronchospasm, stridor, reduced peak expiratory flow, hypoxemia)

• Reduced blood pressure or associated symptoms of end-organ dysfunction (eg, hypotonia [collapse], syncope, incontinence).

3.6. Concomitant Therapy

3.6.1. Prior Treatments

Any prior therapy including but not limited to treatment for COVID -19 within 30 days prior to signing informed consent will be recorded.

Prior therapy information should include:

- Drug Name;
- Reason for use;
- Dates of administration including start and end dates;
- Dosage information including administration route, dose and frequency.

Prior therapy with antivirals including Remdesivir® or other therapeutic agents (e.g. corticosteroids) are permitted.

Prior therapy with convalescent plasma or anti-SARS-CoV-2 S protein targeted therapy are prohibited.

3.6.2. Concomitant Therapy

All therapies during the study should be recorded in the source document and eCRF.

Any medication including but not limited to over the counter or prescription medicines, vitamins, and/or herbal supplements, or other specific categories of interest must be recorded along with:

- Drug Name;
- Reason for use;
- Dates of administration including start and end dates;
- Dosage information including administration route, dose and frequency.

Any non-medication interventions must be recorded along with:

- Name of the interventions;
- Reason for use;

- Dates of intervention including start and end dates.

Any experimental treatment, anti-SARS-CoV-2 S protein targeted therapy and use of convalescent plasma are prohibited.

Currently, there are no data on the safety and efficacy of COVID-19 vaccine in persons who have been treated with anti-SARS-CoV-2 antibody. The anti-SARS-CoV-2 antibody like SCTA01 may interfere with the effectiveness of a COVID-19 vaccine. Considering the half-life of SCTA01, COVID-19 vaccination should not be administered for at least 120 days after the administration of SCTA01.

Best Supportive Care for Severe COVID-19:

Best supportive care (BSC) (including medications and non-medication interventions) for severe COVID-19 is allowed based on the local BSC policies or guidelines.

Medications for BSC may be constituted of but not limited to:

- Antiviral Therapy: Remdesivir®; others;
- Immune-Based Therapy: Dexamethasone; Prednisone; Methylprednisolone; Hydrocortisone; others;
- Adjunctive Therapy: Thrombolytic Therapy; others;
- Others.

Non-medication interventions for BSC may be constituted of but not limited to:

- Supplemental oxygen;
- High flow oxygen;
- Non-invasive ventilation;
- Invasive mechanical ventilation;
- ECMO;
- Others.

3.7. Replacement of Subjects

As the sample size determination is based on the number of patients required for the ITT analysis, patients who are randomly assigned and subsequently withdraw prior to completion of the study will not be replaced.

3.8. End of Study Definition

A participant is considered to have completed the study if he/she has completed all phases of the study including the last visit or the last scheduled procedure shown in the Table 4.

The end of the study is defined as the date of the last visit of the last participant in the study or last scheduled procedure shown in the Table 4 for the last participant in the trial globally.

4. SUBJECT ELIGIBILITY

4.1. Inclusion Criteria

1. Hospitalized patients with severe COVID-19 (5 point on the 8-point ordinal scale). According to the NIH definition, severe patients are those individuals who have one of the following: 1) respiratory rate > 30 breaths per minute; 2) $SpO_2 \leqslant 93\%$ on room air at sea level; 3) $PaO_2/FiO_2 < 300$ mmHg or $SpO_2/FiO_2 \leqslant 315$ mmHg; 4) lung infiltrates > 50%. In high altitude area (over 1000 meters), PaO_2/FiO_2 should be corrected according to the following formula: $PaO_2/FiO_2 \times$ [760/barometric pressure (mmHg)][30]; 5 point: Hospitalized, requiring supplemental oxygen;
2. Subject (or authorized legal representative) has provided informed consent before any clinical activity related to the trial;
3. Subject (or authorized legal representative) understands and agrees to comply with the study procedures;
4. Male or female adult ⩾18 years of age at the time of enrollment;
5. Has documented laboratory-confirmed SARS-CoV-2 infection as determined by polymerase chain reaction (PCR). Note: Patient can be enrolled before SARS-CoV-2 infection has been confirmed and can be tested by any approved clinical test other than PCR as long as SARS-CoV-2 infection is then confirmed by PCR;
6. ⩽ 14 days since symptoms of COVID-19 onset;
7. Eligible COVID-19 vaccinated patients at the time of enrollment are allowed;
8. Women of childbearing potential (see Section 7.2.7) must agree to practice abstinence or use effective contraceptive methods with a failure rate of less than 1% per year during the study period; Male participants must agree to be abstinent from sexual intercourse or agree to use a male condom when engaging in any activity that allows for passage of ejaculate to another person during the study period.

4.2. Exclusion Criteria

1. Hospitalized patients who need non-invasive ventilation or high flow oxygen (i.e., 6 point on the 8-point ordinal scale);
2. Patients with critical COVID-19. According to the NIH definition, critical patients are those individuals who have one of the following: 1) respiratory failure and need invasive mechanical ventilation; 2) septic shock; 3) multiple organ dysfunction;
3. Patients with severe COVID-19 who received convalescent plasma or anti-SARS-CoV-2 S protein targeted therapy;
4. ALT or AST is 5 times higher than the upper limit normal range;
5. eGFR < 30 mL/min or on dialysis{eGFR calculated by Cockcroft-Gault formula (*Cockcroft DW, 1976*), Male: CrCL (mL/min) = [(140 - age) × weight (kg)] × 1/ [SCr

(mg/dL) × 72]; Female: CrCL (mL/min) = [(140 - age) × weight (kg)] × 0.85/ [SCr (mg/dL) × 72]};

6. Pregnant or lactating women;
7. Anticipated discharge from the hospital or transfer to another hospital which is not a study site within 72 hours;
8. A patient who is allergic to the active ingredients or components of SCTA01;
9. Patients participated in other clinical studies related to COVID-19 or SARS-CoV-2 infection within 30 days before enrollment or five half-lives of investigational product, whichever is longer.

4.3. Screen Failures

Screen failures are defined as participants who consent to participate in the clinical study but are not subsequently randomly assigned to investigational product/entered in the study. A minimal set of screen failure information is required to ensure transparent reporting of screen failure participants, which includes demography, screen failure details, eligibility criteria, and any serious adverse event (SAE).

Individuals who do not meet the criteria for participation in this study (screen failure) may not be rescreened.

5. INVESTIGATIONAL PRODUCT MANAGEMENT AND ADMINISTRATION

5.1. Investigational Product

SCT will provide the investigators with the investigational product. SCTA01 will be provided as a sterile, liquid solution. Each glass vial contains 10 mL of study medication with a concentration of 25 mg/mL. The vial is designed for single use only and is not to be used to treat more than one subject. The supplied matching placebo is identical in physical appearance to that of the active injection and contains the same inactive ingredients which include histidine, sodium chloride, polysorbate 80, hydrochloric acid and injection grade water. They should be stored under refrigeration at 2~8°C (36-46°F) in the original carton to protect from light and avoid repeated freezing/thawing. Please refer to the Investigator Brochure for detailed investigational product information.

5.2. Administration

Each participant will receive a single intravenous infusion of either SCTA01 or placebo. Infusion volume will be 500 mL and infusion time is 3 hours (±10 minutes).

Phase II part of the study: Subjects will receive a single dose of SCTA01 (15 mg/kg), SCTA01 (50 mg/kg), or placebo within 24 hours after randomization.

Phase III part of the study: Subjects will receive a single dose of SCTA01 (X mg/kg or X mg) or placebo within 24 hours after randomization.

Further instruction on the preparation and administration of the investigational product will be provided to the site in Pharmacy Manual.

5.3. Management of the Investigational Product

5.3.1. Packaging and Labeling

The vial and carton labels will contain the drug name, dosage form, lot number, method of use, storage conditions, shelf life, manufacturer and other information that may be required by the regulatory agency, and indicate "investigational use only" in accordance with regional regulations.

5.3.2. Receipt

Upon receipt of the investigational product, the investigator or designee will verify that an appropriate shipping temperature has been maintained, conduct an inventory count, sign the drug receipt form and send a scanned copy to the sponsor contact as designated on the form. If a temperature excursion occurs, the sponsor should be notified immediately, and the investigational product must be quarantined and maintained under the correct storage conditions until further instructions are provided by the sponsor. The temperature excursion form will need to be completed and returned to the sponsor. The original drug receipt form and packing slip must be retained in the investigator's pharmacy records.

5.3.3. Storage

Records of the actual storage conditions during the period of the study must be maintained (e.g., records of the date and time and the initials of the person performing the check, the temperatures of the

refrigerator used for storage of trial supplies, continuous temperature recordings or regularly maintained temperature alarm systems used in conjunction with temperature recording). If a temperature excursion occurs, the sponsor should be notified immediately, and the investigational product must be quarantined and maintained under the correct storage conditions until further instructions are provided by the sponsor.

5.4. Accountability and Destruction

The investigator or designee(s) must maintain an inventory record of the investigational product received, dispensed, administered, and returned. The investigator or designee(s) should record the dispensing of the study drug to individual subjects and disposition of any remaining study drug after dosing in the investigational product accountability record. Investigational product accountability records and temperature monitoring logs must be made available to the sponsor or designated monitoring personnel for the purpose of conducting drug accountability.

All unused medication is not to be stored or subsequently used and will need to be properly disposed of in accordance with the institutional policy and procedure, and disposition must be recorded on the investigational product accountability record.

The sponsor or designee will provide guidance on the destruction of unused investigational product. If destruction is authorized to take place at the investigator site, the investigator must ensure that the materials are destroyed in compliance with applicable environmental regulations, institutional policy, and any special instructions provided by SCT, and all destruction must be adequately documented.

5.5. Return of Drugs

A representative from the sponsor or designee will inspect the study drug inventory, perform accountability, and will arrange for the disposition of any remaining unused study drug. No study drug may be returned to the sponsor designee without the representative from the sponsor or designated personnel first inspecting the study drug inventory and accountability documentation.

5.6. Randomization and Blinding

Each subject will be assigned a unique identification number. The subject identification (SID) number will be used to identify the subject throughout the study and will be entered on all study documentation. The SID will be an 8-digit number with the first 4 digits corresponding to the site number and the last 4 digits sequentially assigned by the site at screening. If a subject discontinues from the study at any time, his/her study number cannot be reassigned to another subject.

Phase II part of the study: Patients will be randomly assigned to receive SCTA01 (15 mg/kg), SCTA01 (50 mg/kg) or placebo a 1:1:1 ratio. The randomization schedule will be stratified by country.

Phase III part of the study: Patients will be randomly assigned to receive SCTA01 (X mg/kg or X mg) or placebo in a 1:1 ratio. The randomization schedule will be stratified by country.

An interactive web response system (IWRS) will be used to administer the randomization schedule. An independent CRO statistician will generate a permuted block randomization schedule for IWRS, which will link the sequential patient randomization numbers to the treatment codes.

Each patient will be assigned a randomization number which is different from the patient identification number. Once a randomization number has been allocated to a patient, it cannot be assigned to another patient.

To ensure the treatment assignment is unbiased and concealed from all individuals involved in the study, all enrolled patients are randomized to SCTA01 or placebo group using the following procedures: 1) randomization data are kept strictly confidential until the time of final analyses, and will not be accessible to anyone involved in the conduct of the study with the exception of the IDMC team and unblinded statistician; 2) the identity of the treatments will be concealed by the use of study drugs (SCTA01 and Matching Placebo) that are all identical in packaging, labeling, schedule of administration and appearance.

5.7. Other Considerations

The investigator should administer the investigational drug or placebo to the subjects according to the protocol. Medication errors should be reported to the sponsor/CRO immediately as a protocol deviation. Medication errors can cause AE/SAE or clinical abnormal results.

6. STUDY INTERVENTION DISCONTINUATION

6.1. Individual Infusion Halting

In rare instances, it may be necessary for a subject to permanently discontinue study intervention. If study intervention is definitively discontinued, the participant will remain in the study for follow-up.

For an individual subject, the infusion must be terminated and not restarted if severe infusion-related reaction(s) mentioned in Section 3.5 occur.

The treatment for any given subject may be stopped for SUSARs related to SCTA01, clinically significant adverse reactions, severe laboratory abnormalities, or any other medical conditions that indicate to the investigator that continued dosing is not in the best interest of the patient.

In addition, a subject in this clinical study may discontinue study drug at their requests for any reason. After the discontinuation, every effort should be made to encourage the subject to remain in the study for the duration of their planned outcome assessments.

The reason for subject discontinuation of study drug should be documented in the case report form.

6.2. Study Halting

Given the potential severity of COVID-19, there are no pre-specified study stopping rules. Instead there will be close oversight by the project team and frequent IDMC reviews of the safety data.

The clinical trial should be terminated if there are any of the following situations：

- The sponsor finds that SCTA01 has potential safety hazards or the research has quality problems and requires the trial to be terminated completely;
- The IDMC evaluates the clinical trial results and believes there is a potentially significant safety risk;
- The relevant government authorities request the termination of the trial.

6.3. Withdrawal from the Study

Subjects are free to withdraw from participation in the study at any time upon request, without any consequence. Subjects should be listed as having withdrawn consent only when they no longer wish to participate in the study and no longer authorize the Investigators to make efforts to continue obtaining their outcome data. Subjects who withdraw from this study or are lost to follow-up after signing the informed consent form (ICF) and administration of the study product, will not be replaced. The reason for subject withdrawal from the study will be recorded on the appropriate CRF.

6.4. Lost to Follow-Up

A subject will be considered lost to follow-up if he or she fails to complete all follow-up assessments. In such cases, attempts to contact the subject should be made and these efforts should be documented in the subject's records.

Patients transmitted to comfort care are not considered lost to follow up, but the outcome of the patient should be collected.

7. STUDY PROCEDURES AND EVALUATIONS

All required study procedures and evaluations are to be conducted as outlined in this protocol. In the event of a deviation from the protocol due to an emergency, accident, or mistake, the investigator or designee must notify the sponsor as soon as possible.

Additional assessments (vital signs, ECG, laboratory test, etc.) can be done at the discretion of the investigators as clinically indicated.

7.1. Time and Events Schedule

The schedule of study assessments is provided in Tables 2, 3, and 4 below. Flexibility is allowed in scheduling the visits according to visit time windows as specified in table below.

For a procedure required at early withdraw (Table 4), it does not need to be performed if the procedure is not a daily required procedure and it has been done in 3 days before subject's withdraw.

Table 2. Schedule for Screening Visit

**Study Process**	**Screening Period**	**Comments**
Visit	V1	
Visit day	D-3~ D-1	
Informed consent	X	
Demographics	X	Including birth date, sex, race, and ethnicity.
Medical history	X	Medical history prior to signing the ICF including:<br>− The day of onset of COVID-19 signs and symptoms.<br>− History of medication allergies.<br>− History of medical conditions within 30 days prior to signing the ICF including but not limited to chronic oxygen requirement prior to onset of COVID-19.<br>− Ask if they are participating in another clinical trial or plan to enroll in another clinical trial in the next 30 days.
Physical examination	X	Testing items see Section 7.2;<br>Physical examination is focused on lung auscultation.
β-HCG	X	For non-postmenopausal female subjects or who have not undergone surgical sterilization
Serum chemistry	X	Testing items see Section 7.2.
SARS-CoV-2 infection confirmation	X	Has documented laboratory-confirmed SARS-CoV-2 infection as determined by PCR. Note: Patient can be enrolled before SARS-CoV-2 infection has been confirmed and can be tested by any approved clinical test other than PCR as long as SARS-CoV-2 infection is then confirmed by PCR.
Vital signs	X	Include body temperature, pulse rate, blood pressure, respiratory rate in semi-supine position
Diagnosis of severe COVID-19 infection	X	Use one of following criteria: 1) respiratory rate; 2) SpO_2; 3) PaO_2/FiO_2 or SpO_2/FiO_2; 4) chest X-ray or CT. Refer to Section 4.1 for details.
8-Point Ordinal Scale	X	
Inclusion/Exclusion criteria review	X	
AEs	X	Record the AEs from after obtaining the Informed Consent
Prior treatments within the last 30 days	X	

Table 3. Schedule for Treatment Visit

**Study Process**	**Treatment Period**	**Comments**
Visit	V2	
Visit day	D1	
Randomization	X	
Height	X	
Weight	X	
Vital signs	X	Refer to Section 7.2.2.<br>At 15±5, 30±5, 45±5, 60±5, 90±10, 120±10, 150±10, 180±10, 240±10, 300±10 minutes after the beginning of infusion (i.e. every 15±5 minutes for the first hour of infusion, every 30±10 minutes until the end of infusion and every 60±10 minutes for 2 hours after the end of infusion).
Targeted physical examination	Are performed only when needed to evaluate possible adverse events.	
12-lead ECG	X	Refer to Section 7.2.3<br>Test within 2 h (± 10 min) before infusion, and within 2 h (± 10 min) after the end of infusion.<br>Additional ECG examination is at the discretion of the Investigator per standard clinical practice or as clinically indicated.
Hematology	X	Testing items see Section 7.2.5.<br>Sample will be collected before infusion.
Serum chemistry	X	
Urinalysis	X	
Coagulation test	X	
IL-6	X	
Ferritin	X	
Procalcitonin	X	
Thrombin time	X	
D-dimer	X	
Serological samples for anti-spike protein antibodies	X	Blood samples will be collected within 0.5 h (± 5 min) before infusion.
Immunogenicity (ADA) blood samples	X	
PK samples	X	Intensive sampling of Phase II part:<br>− Within 0.5 h (± 5 min) before the beginning of infusion; 1 h (±10 min) post end of infusion.
Blood for PCR SARS-CoV-2 test	X	Sample will be collected before infusion.

NP or OP swab for SARS-CoV-2 test	X	Sample will be collected before infusion.<br>NP swab preferred. If NP swab cannot be obtained, OP swab may be used.
SpO_2	X	Record SpO_2 before infusion, and 1 h (±10 min), 2 h (±10 min), 3 h (±10 min) after the end of infusion.<br>Also record oxygen supplementation: low flow oxygen, high flow oxygen, or other.
8-Point Ordinal Scale	X	To evaluate within 30 ~ 60 min before infusion.
Administration of SCTA01/Placebo	X	The dose should be given within 24 hours after randomization.
AEs	X	
Concomitant medications	X	Items see Section 3.6.1.

Table 4. Schedule for Follow-up Visit

Study Process	Follow-up Period											Comments
Visit	V3	V4	V5	V6	V7	V8	V9	V10	V11	V12	Early Withdraw	
Visit day	D2	D3	D5	D8	D11	D15	D22	D29	D60	D120		
Window period (± day)	±0 d	±0 d	±1 d	±1 d	±1 d	±2 d	±3 d	±3 d	±3 d	±7 d		
Vital signs		Daily until discharge										Testing items see Section 7.2.2.
Targeted physical examination		Performed only when needed to evaluate possible adverse events										Only performed while patient is in hospital or attends an in-person visit.
β-HCG	Only when potential pregnancy is suspected and at the end of the study										X	
12-lead ECG		X		X		X		X		X	X	Testing items see Section 7.2.3 and 7.2.5.<br>Only performed while patient is in hospital or attends an in-person visit.<br>Additional examination is at the discretion of the Investigator.
Hematology		X		X		X		X		X	X	
Urinalysis		X		X		X		X		X	X	
Serum chemistry		X		X		X		X		X	X	
Coagulation test		X		X		X		X		X	X	
IL-6		X		X		X		X		X	X	
Ferritin		X		X		X		X		X	X	
Procalcitonin		X		X		X		X		X	X	
Thrombin time		X		X		X		X		X	X	
D-dimer		X		X		X		X		X	X	
Immunogenicity blood samples								X		X	X	

PK samples				X[1, 2]		X[1]		X[1, 2]			X[1, 2]	[1]: Intensive sampling of Phase II part: Blood samples will be collected in the same 30 subjects as the Table 3.<br>[2]: Sparse sampling of Phase II part: Blood samples will be collected in the remaining subjects (excluding the 30 subjects with intensive PK sampling) of each group of Phase II part.
Blood for SARS-CoV-2 test		X		X		X		X		X	X	Only performed while patient is in hospital or attends an in-person visit.
NP or OP swab for SARS-CoV-2 test		X	X	X	X	X		X		X	X	Continue testing at the scheduled time-point while hospitalized or attends an in-person visit, until the NP or OP swab samples viral RNA test is negative.<br>NP swab preferred. If NP swab can' t be obtained, OP swab may be used.
Sequencing the gene encoding spike protein		Will be tested in subjects who do not achieve a reduction in viral shedding or persistence viral presence or have a rebound in viral shedding following suppression										Using reserved NP or OP swab samples. No additional sampling is required.
8-Point Ordinal Scale	Daily until discharge					X	X	X			X	Only performed while patient is in hospital or attends an in-person visit, otherwise performed by phone call to assess clinical status (ordinal scale).<br>The date and cause of discharge or re-hospitalization after discharge should be recorded.

Concomitant medications		Daily until discharge	
SpO_2		Daily until discharge	Report the best reading if multiple readings are obtained in a day.<br>Also record oxygen supplementation: low flow oxygen, high flow oxygen, or other.
AEs		Daily until discharge and biweekly via phone call after discharge until 120 days.	
ADE		Monitor for new infections and adverse events until 120 days (if discharged, via biweekly telephone call, in-person or virtual visit).	

7.2. Safety Assessments

Planned time points for all safety assessments are provided in the Tables 2 to 4. Unscheduled clinical laboratory measurements may be obtained at any time during the study to assess any perceived safety concerns.

7.2.1. Physical examinations

A complete physical examination will include, at a minimum, assessments of the cardiovascular, respiratory, gastrointestinal, and neurological systems.

A complete physical examination will be performed at the screening visit.

A targeted physical examination will be performed only when needed to evaluate possible adverse event (s) (i.e. any new signs or symptoms). No routine physical exam is needed for study visits after Day 1.

7.2.2. Vital signs

Vital signs will be measured as specified in the Tables 2 to 4 and as clinically indicated. Vital signs will be measured with the participant in a semi-supine position after 5 minutes of rest and will include:

- Body temperature
- Pulse rate
- Systolic and diastolic blood pressure
- Respiratory rate

Additional vital signs may be measured during the study if warranted, as determined by the investigator.

7.2.3. Electrocardiograms

12-Lead ECGs should be collected at times specified in the Tables 3 and 4 using an ECG machine that automatically calculates the heart rate and measures PR interval, QT interval, and QTc intervals and QRS time limit. All scheduled ECGs should be performed after the participant has rested quietly for at least 10 minutes in a supine position. Generally, ECGs should not be collected within 3 hours after food or beverage consumption.

QTcB and QTcF will be analyzed in SAP.

If a postdose QTc interval remains 30 msec from the baseline and is >450 msec; or an absolute QTc value is 500 msec for any scheduled ECG for greater than 4 hours (or sooner, at the discretion of the investigator), or QTc intervals get progressively longer, the participant should undergo continuous ECG monitoring. A cardiologist should be consulted if QTc intervals do not return to less than the criterion listed above after 8 hours of monitoring (or sooner, at the discretion of the investigator).

In some cases, it may be appropriate to repeat abnormal ECGs to rule out improper lead placement as contributing to the ECG abnormality. It is important that leads be placed in the same positions each time in order to achieve precise ECG recordings. If a machine read QTc value is prolonged,

as defined above, repeat measurements may not be necessary if a qualified medical provider's interpretation determines that the QTc values are in the acceptable range.

ECG will be in digital format and be read locally.

7.2.4. SpO_2

Record SpO_2 while participant is at rest, at the same time record oxygen supplementation: low flow oxygen, high flow oxygen, or other. Record FiO_2 if known, and method of delivery, if applicable.

7.2.5. Clinical Safety Laboratory Assessments

Fasting is not required before collection of laboratory samples; Day 1 clinical laboratory tests are performed prior to infusion as a baseline.

Blood will be collected at the time points indicated in the Tables 2 to 4. Testing items including:

- Hematology: Red blood cell count; hemoglobin concentration; platelet count; white blood cell count; absolute value and percentage of: lymphocytes, monocytes, neutrophils, eosinophils, basophils;
- Urinalysis: Urine occult blood, urine bilirubin, urobilinogen, urine ketone body, urine protein, urine nitrite, urine glucose, urine pH, urine specific gravity, urine white blood cells, urine white blood cells (high power field), urine red blood cells, and urine red blood cells (high power field);
- Serum chemistry: AST, ALT, total bilirubin, direct bilirubin, indirect bilirubin, total protein, alkaline phosphatase, glutamyl transpeptidase, urea, blood creatinine, albumin, creatine kinase, C-reactive protein, blood glucose, potassium, and sodium, chlorine, lactate dehydrogenase (LDH);
- Coagulation test: Prothrombin time, activated partial thromboplastin time, fibrinogen;
- IL-6; Ferritin; Procalcitonin; Thrombin time; D-dimer.

The investigator must review the laboratory report, document this review, and record any clinically relevant changes occurring during the study in the AE section of the CRF. Clinically significant abnormal laboratory findings are those which are not associated with the underlying disease, unless judged by the investigator to be more severe than expected for the participant's condition.

All laboratory tests with values considered clinically significantly abnormal during participation in the study should be repeated until the values return to normal or baseline or are no longer considered clinically significant by the investigator or medical monitor.

If such values do not return to normal/baseline within a period of time judged reasonable by the investigator, the etiology should be identified and the sponsor notified.

All protocol required laboratory assessments must be conducted in accordance with the laboratory manual.

If laboratory values from non-protocol specified laboratory assessments performed at the institution's local laboratory require a change in participant management or are considered

clinically significant by the investigator (e.g., SAE or AE or dose modification), then the results must be recorded in the CRF.

7.2.6. ADE Assessments

Monitor for new infections and adverse events until 120 days (if discharged, via biweekly telephone visit, in-person or virtual visit).

7.2.7. Pregnancy Testing

Pregnancy tests are serum tests, but must have a sensitivity of at least 25 mIU/mL. Women of childbearing potential (WOCBP) must undergo pregnancy testing according to the Tables 2 to 4. Following a negative pregnancy test result at screening, appropriate contraception must be commenced. Pregnancy tests will also be done whenever 1 menstrual cycle is missed during the study (or when potential pregnancy is otherwise suspected) and at the end of the study. Pregnancy tests may also be repeated if requested by institutional review boards (IRBs)/ethics committees (ECs) or if required by local regulations.

Participants who are pregnant will be discontinued from the study.

7.3. Clinical Efficacy Assessments

For all baseline assessments and follow-up visits, refer to the Tables 3 and 4 for procedure to be done, and details below for each assessment.

7.3.1. Time to Clinical Improvement

The subject's clinical status will be evaluated using the 8-point ordinal scale every day until Day 29 while being hospitalized. However, if the subject is discharged from the hospital before Day 15, Day 22, or Day 29, he/she only needs to come back to the hospital on Day 15, Day 22, and/or Day 29 for evaluation along with completing other procedures. If the discharged subject is unable to return for visits on these days, the evaluation can be done over the phone.

7.3.1.1 8-Point Ordinal Scale

The ordinal scale is the primary measure of clinical outcome. The scale used in this study is as follows (from worst to best):

8: Death;

7: Hospitalized, on invasive mechanical ventilation or ECMO;

6: Hospitalized, on non-invasive ventilation or high flow oxygen devices;

5: Hospitalized, requiring supplemental oxygen;

4: Hospitalized, not requiring supplemental oxygen - requiring ongoing medical care (COVID-19 related or otherwise);

3: Hospitalized, not requiring supplemental oxygen - no longer requires ongoing medical care;

- This would include those kept in hospital for quarantine/infection control, awaiting bed in rehabilitation facility or homecare, etc.;

2: Not hospitalized, limitation on activities and/or requiring home oxygen;

1: Not hospitalized, no limitations on activities.

To determine a subject's clinical status using the ordinal scale, the investigator should:

On Day 1, report their clinical status at randomization; On Day 2, report the clinical status from randomization to midnight on Day 1; On Day 3 through Day14 or until discharged and On Days 15, 22 and 29, provide the worst clinical assessment for the previous day [i.e., midnight to midnight; 00:00 ~ 23:59 (24-hr clock)]. For example, on Day 3 when completing the form, the worse clinical outcome measure of Day 2 is captured with the worst being death followed by ECMO, mechanical ventilation, etc. The Day 2 measurement is assessed as occurring anytime in that 24-hour period (00:00 ~ 23:59).

7.4. Viral Load Assessments

Samples for virologic assessments should be collected using standardized methodology and analyzed in a central laboratory.

7.4.1. Viral Load Assessment in NP or OP Swab Samples

As outlined on the Tables 3 and 4, NP or OP swab samples will be collected on Day 1; and Days 3, 5, 8, and 11 (while hospitalized); and on Days 15, 29, 120 (if attends an in-person visit or still hospitalized) or at early withdraw. Up to 29 days after administration, subjects who are virus positive should continue to undergo regular viral testing by NP or OP swab samples until the viral RNA test is negative.

NP samples should be taken from the same nostril for all tests.

If NP swab samples are not obtainable, oropharyngeal (OP) swab samples may be substituted, but make sure the position of swabs are consistent for the same subject.

Quantitative PCR for NP or OP swab samples will be done at central lab.

7.4.2. Viral Load Assessment in Blood Samples

As outlined on the Tables 3 and 4, plasma blood samples to test for SARS-CoV-2 by RT-PCR will be collected at the scheduled time-point while hospitalized or attends an in-person visit.

Quantitative PCR for blood samples will be performed at central lab.

7.5. Non-Response to SCTA01 Assessments

Samples collected for viral assessment via NP or OP swabs can be probed for the non-responder having mutations.

A non-responder is defined as a patient who does not achieve a reduction in viral shedding or persistence viral presence or have a rebound in viral shedding following suppression.

Spike protein gene sequencing should be performed to evaluate the non-responders having mutations in the gene encoding the SARS-CoV-2 S protein.

7.6. Pharmacokinetics Assessments

Base on the PK data obtained in this study as well as PK data obtained from other studies (if available), a population PK analysis will be performed. Details on the collection of blood samples, processing, storage, and shipping details of serum samples are provided in the Laboratory Manual.

7.6.1. Intensive PK Sampling

Blood samples for this PK analysis will be collected in about 30 randomly selected subjects in Phase II part of the study at the following time points: within 0.5 h before the beginning of infusion; 1 h (± 10 min) post end of infusion; D8 (± 1 D), D15 (± 2 D), D29 (± 3 D) post end of infusion or at early withdraw.

7.6.2. Sparse PK Sampling

Blood samples for this PK analysis will be collected in the other subjects (excluding the 30 subjects for intensive PK sampling) of each group in Phase II part of the study at the following time points: D8 (± 1 D), D29 (± 3 D) or at early withdraw.

Sparse PK sampling will be done at the site with PK sampling capability and is at the discretion of subject, sparse PK data will be used for population PK analysis.

7.7. Immunogenicity Assessments

Samples for immunologic assessments should be collected and analyzed using validated method in a central laboratory.

Blood samples will be collected to evaluate anti-SCTA01 antibodies in serum at the points within 0.5 h before the beginning of infusion, D29 (± 3D) and D120 (± 7D) or at early withdraw.

The ADA immunoassay methods including screening, confirmation, and titer will be developed and validated. Once a test sample is confirmed positive for the presence of the anti-SCTA01 antibody, the sample will be assessed to determine if the anti-SCTA01 antibody is neutralizing via a CLB Nab assay. Samples will be collected and stored for the anti-SCTA01 antibody specificity assessments, which will occur at a later time when the assays have been developed and validated.

Details on collection of blood samples, processing, storage, and shipping details of serum samples for anti-SCTA01 antibody evaluations are provided in the Laboratory Manual.

8. ADVERSE EVENTS

8.1. Monitoring, Recording, and Reporting of Adverse Events

An adverse event (AE) is any unfavorable and unintended sign (including an abnormal laboratory finding, for example), symptom, or disease temporally associated with the use of a medicinal product, whether or not considered related to the medicinal product. It may be a new intercurrent illness, a worsening concomitant illness, an injury, or any concomitant impairment of the subject's health, including laboratory test values, regardless of etiology. Any worsening (i.e., any clinically significant adverse change in the frequency or intensity of a pre-existing condition other than disease under study) should be considered as an AE. A diagnosis or syndrome should be recorded on the AE page of the CRF rather than the individual signs or symptoms of the diagnosis or syndrome.

The signs/symptoms/clinical sequelae resulting from COVID-19 are not considered as AEs. The deaths resulting from COVID-19 that are consistent with the clinical endpoint definition should not be recorded as AEs. These data will be captured as efficacy assessment data on the relevant pages of the CRF, as they are expected endpoints.

AEs may also include pre-treatment or post-treatment complications that occur as a result of protocol specified procedures, lack of efficacy, overdose, drug abuse/misuse reports, or occupational exposure. Pre-existing events that increase in severity or change in nature during or as a consequence of participation in the clinical study will also be considered as AEs. All AEs will be recorded by the investigator from the time the subject signs the ICF through Day 120 after the last dose of the study drug or until the last study visit, whichever is longer. AEs and SAEs will be recorded on the AE page of the CRF and in the subject's source documents. All SAEs must be reported to Drug Safety of SCT within 24 hours of the investigator's knowledge of the event. Refer to Sections 8.5 and 8.6 for SAE reporting details.

8.2. Evaluation of Adverse Events

A qualified investigator will evaluate all AEs/SAEs and report their seriousness, severity/intensity, relationship to the study drug, duration, action taken, subject outcome, and for final review and confirmation of accuracy of event information and assessments.

8.2.1. Seriousness

An SAE is any AE that fulfils one or more of the following:

- Results in death;
- Is life-threatening (i.e., in the opinion of the investigator, the subject is at immediate risk of death from the AE as it occurred);
- Requires hospitalization or prolongation of existing hospitalization (hospitalization signifies that the participant has been detained at the hospital or emergency ward for observation and/or treatment usually involving at least an overnight stay);
- Results in persistent or significant disability or incapacity (a substantial disruption of the subject's ability to conduct normal life functions);

- Results in a congenital abnormality or birth defect;
- Important medical event:
  - Important medical events are defined as those occurrences that may not be immediately life-threatening or result in death, hospitalization, or disability, but may jeopardize the subject or require medical or surgical intervention to prevent one of the other outcomes listed above. Medical and scientific judgment should be exercised in deciding whether such an AE should be considered serious. In addition, any suspected SCTA01 related SARS-CoV-2 re-infection or enhanced COVID-19 severity, is considered an important adverse event and must be reported as a serious adverse event (SAE).

An AE does not include the following:

- Medical or surgical procedures such as surgery, endoscopy, tooth extraction, or transfusion. The condition that led to the procedure may be an adverse event and must be reported;
- Pre-existing diseases, conditions, or laboratory abnormalities present or detected before the screening visit that do not worsen;
- Situations where an untoward medical occurrence has not occurred (e.g. hospitalization for elective surgery, social and/or convenience admissions);
- Overdose without clinical sequelae;
- Any medical condition or clinically significant laboratory abnormality with an onset date before the ICF is signed and not related to a protocol-associated procedure is not an AE. It is considered to be pre-existing and should be documented on the medical history CRF.

If an AE is considered serious, both the AE page of the CRF and the Individual Case Safety Report **(ICSR) Form** must be completed.

For each SAE, investigator will provide information on severity, start and stop dates, relationship to the study drug, action taken regarding the study drug, and outcome.

8.2.2. Severity/Intensity

The severity/intensity of AEs and SAEs will be graded based upon the subject's symptoms according to the DAIDS Table for Grading the Severity of Adult and Pediatric Adverse Events (Version 2.1, July 2017). The functional table below should be used to grade the severity of an AE that is not specifically identified in DAIDS grading table. In addition, all deaths related to an AE are to be classified as grade 5.

Table 5. Estimating Severity Grade for Parameters Not Identified in the Grading Table

**Parameter**	**Grade 1 Mild**	**Grade 2 Moderate**	**Grade 3 Severe**	**Grade 4 Potentially**
**Clinical** adverse event **NOT** identified	Mild symptoms causing no or minimal	Moderate symptoms causing greater than minimal interference with usual social &	Severe symptoms causing inability to	Potentially life-threatening symptoms causing inability to perform

elsewhere in the grading table	interference with usual social & functional activities with intervention not indicated	functional activities with intervention indicated	perform usual social & functional activities with intervention or hospitalization indicated	basic self-care functions with intervention indicated to prevent permanent impairment, persistent disability, or death

It is important to distinguish between serious and severe AEs. Severity is a measure of intensity whereas seriousness is defined by the criteria under Section 8.2.1. An AE of severe intensity may not be considered serious. Seriousness, not severity, serves as a guide for defining regulatory obligations.

8.2.3. Event Relationship to Study Drug

The investigator is obligated to assess the relationship between study drug therapy and the occurrence of each AE/SAE:

- A “reasonable possibility” of a relationship conveys that there are facts, evidence, and/or arguments to suggest a causal relationship, rather than a relationship cannot be ruled out.
- The investigator will use clinical judgment to determine the relationship.
- Alternative causes, such as underlying disease(s), concomitant therapy, and other risk factors, as well as the temporal relationship of the event to study drug administration, will be considered and investigated.
- For each AE/SAE, the investigator must document in the medical notes that he/she has reviewed the AE/SAE and has provided an assessment of causality.
- There may be situations in which an SAE has occurred and the investigator has minimal information to include in the initial report to the sponsor. However, it is very important that the investigator always make an assessment of causality for every event before the initial transmission of the SAE data to the sponsor.
- The investigator may change his/her opinion of causality in light of follow-up information and send an SAE follow-up report with the updated causality assessment.
- The causality assessment is one of the criteria used when determining regulatory reporting requirements.
- If the investigator does not know whether or not the study drug caused the event, then the event will be handled as “related to study drug” for reporting purposes, as defined by the sponsor.

8.2.4. Duration

For all AEs and SAEs, the investigator will provide a record of the start and stop dates of the event.

8.2.5. Action Taken

The Investigator will report the action taken with the study drug as a result of any AE or SAE, as applicable (e.g., discontinuation of the study drug) and report if concomitant and/or additional treatments were given for the event.

8.2.6. Outcome

The Investigator will report the outcome of the event for both AEs and SAEs. Non-serious AEs will be followed for 120 days after the subject's last dose of the study drug. All SAEs that have not resolved upon discontinuation of the subject's participation in the study must be followed until recovered, recovered with sequelae, not recovered (death due to other cause), and death (due to the SAE), lost to follow up, or otherwise explained.

8.2.7. Adverse Events of Special Interest

Pre-identified AESIs to date include ⩾ Grade 3 acute allergic reactions and ≥ Grade 3 cytokine release reaction (i.e. infusion related reactions). The grading for "acute allergic reaction" and "cytokine release reaction" are specified in the Table 6 below.

Table 6. Grading for Acute Allergic Reaction and Cytokine Release Reaction

Parameter	Grade 1<br>Mild	Grade 2<br>Moderate	Grade 3<br>Severe	Grade 4<br>Potentially
Acute Allergic Reaction	Localized urticaria (wheals) with no medical intervention indicated	Localized urticaria with intervention indicated OR Mild angioedema with no intervention indicated	Generalized urticaria OR Angioedema with intervention indicated OR Symptoms of mild bronchospasm	Acute anaphylaxis OR Life-threatening bronchospasm OR Laryngeal edema
Cytokine Release Reaction	Mild signs and symptoms AND Therapy (i.e., antibody infusion) interruption not indicated	Therapy (i.e., antibody infusion) interruption indicated AND Responds promptly to symptomatic treatment OR Prophylactic medications indicated for ⩽ 24 hours	Prolonged severe signs and symptoms OR Recurrence of symptoms following initial improvement	Life-threatening consequences (e.g., requiring pressor or ventilator support)

All AESI within 24 hours of investigator becoming aware of the events, must be reported on the ICSR Form and reported to Drug Safety Department of SCT and CRO by emailing the completed form to the safety mailbox of CRO (SAEintake@covance.com) and copying to Drug Safety of SCT (SCT-safety@sinocelltech.com).

8.3. Abnormal Laboratory Values

Laboratory abnormalities without clinical significance should not recorded as AEs or SAEs. However, an abnormal laboratory value is considered to be an AE if the abnormality is judged to be of significant clinical significance by investigator.

Regardless of the severity grade, only laboratory abnormalities that fulfill a seriousness criteria need to be documented as an SAE.

If a laboratory abnormality is one component of a diagnosis or syndrome, then only the diagnosis or syndrome ought to be recorded on the AE page of the CRF. If the abnormality was not a part of a diagnosis or syndrome, then the laboratory abnormality ought to be recorded as an AE.

Severity should be recorded and graded according to the DAIDS Table (Version 2.1, July 2017). For adverse events associated with laboratory abnormalities, the event should be graded based on the clinical severity in the context of the underlying conditions; this may or may not be in agreement with the grading of the laboratory abnormality.

8.4. Pregnancy

In the event that a partner of a male study subject becomes pregnant during the study, if the subject agrees, the subject's pregnant partner should be notified of the subject's study participation and be requested to sign a Release of Information form, permitting transfer of information regarding the pregnancy and outcome to the sponsor.

The Investigator should report all pregnancies identified after initiation of study medication and throughout the study in the female subjects or a woman whose male partner is the trial participant, including the post study drug follow-up period, to Drug Safety Department of SCT and CRO using the Pregnancy Report Form within 24 hours of becoming aware of the pregnancy by emailing the completed form to the safety mailbox of CRO (SAEintake@covance.com) and copying Drug Safety of SCT (SCT-safety@sinocelltech.com).

The pregnancy itself is not considered an AE nor is an induced elective abortion to terminate a pregnancy without medical reasons.

Any premature termination of pregnancy (e.g., a spontaneous abortion, an induced therapeutic abortion due to complications or other medical reasons) must be reported within 24 hours as an SAE. The underlying medical reason for this procedure should be recorded as the AE term.

A spontaneous abortion is always considered to be an SAE and will be reported as described in Section 8.5. Furthermore, any SAE occurring as an adverse pregnancy outcome post study must be reported to Drug Safety Department of SCT and CRO.

The subject should receive appropriate monitoring and care until the outcome of the pregnancy. The outcome should be reported to Drug Safety Department of SCT and CRO using the pregnancy report form. If the end of the pregnancy occurs after the study has been completed, the outcome should be reported directly to Drug Safety of SCT.

8.5. Reporting Serious Adverse Events

Any AE that meets the criteria for an SAE requires the completion of an ICSR Form in addition to being recorded on the AE page of the CRF. All SAEs must be reported to Drug Safety Department of SCT and CRO within 24 hours of the Investigator's knowledge of the event using the Individual Case Safety Report (ICSR) Formby sending email to the safety mailbox of CRO (SAEintake@covance.com) and copying to Drug Safety of SCT (SCT-safety@sinocelltech.com). This instruction pertains to initial SAE reports as well as any follow-up reports.

The investigator is required to ensure that the data on these forms are accurate and consistent. This requirement applies to all SAEs (regardless of relationship to the study drug) that occur during the

study (from the time the subject signs informed consent to Day 120 after the last dose of the study drug), and those made known to the investigator at any time thereafter that are suspected of being related to the study drug. AEs/SAEs occurring prior to treatment will be captured.

The SAE report should provide a detailed description of the SAE and include summaries of hospital records and other relevant documents. If a subject died and an autopsy has been performed, copies of the autopsy report and death certificate are to be sent to Drug Safety of SCT as soon as these become available. Any follow-up data will have to be detailed in a subsequent ICSR Form and sent to Drug Safety of SCT.

Where required by local legislation, the investigator is responsible for informing the IRB or independent ethics committee (IEC) of the SAE and providing them with all relevant initial and follow-up information about the event. The Investigator must keep copies of all SAE information on file including correspondence with CRO and the IRB/IEC.

8.5.1. Safety Queries

Queries pertaining to SAE/AESI/Pregnancy will be communicated from Drug Safety Department of CRO to the site via electronic mail. The expected response time is no more than five calendar days. Urgent queries (e.g., missing causality assessment) may be handled by phone, electronic mail or facsimile

8.6. Expedited Reporting of Adverse Events

For events considered to be related to the study drug by investigator, Drug Safety of SCT will determine the expectedness of events suspected of being related to the SCTA01 based on the Reference Safety Information (RSI) of the Investigator Brochure.

All investigators will receive a safety letter notifying them of relevant suspected unexpected serious adverse reaction (SUSAR) reports. The Investigator should notify the IRB or IEC of SUSAR reports as soon as is practical, where this is required by local regulatory agencies, and in accordance with the local institutional policy.

The investigator must keep copies of all pertinent safety information on file including correspondence with CRO and the IRB/IEC.

Investigators may also refer to the Serious Adverse Event Report Form/Completion Guidelines or to the Pregnancy Report Form/Completion Guidelines.

9. STATISTICAL METHODS

9.1. General Principle

All statistical analyses will be done with the SAS software Version 9.4 or later. This is a double-blind, placebo controlled randomized trial with a one-sided Type I error rate of 0.025. Continuous variables will be summarized using descriptive statistics. Categorical variables will be summarized using frequency counts and percentages of patient incidence.

9.2. Statistical Hypotheses

The null hypothesis is that there is no treatment group difference for the primary efficacy endpoint and the alternative hypothesis is that a treatment group difference exists.

9.3. Statistical Decision Rules

A combined *p*-value using inverse normal method of less than 1-side of 0.01068 from 3rd interim analysis or less than 1-side of 0.0166 from final analysis will need to be observed to declare statistical significance. The total number of patients in the ITT analysis sets for primary analysis will include patients in the recommended dose group in Phase II and in the same dose group that continued in Phase III, or the control group. i.e., 95 + 255 = 350 patients for recommended dose group or for control group. The patients from the inferior dose group (95 patients) in Phase II will only contribute to the multiplicity penality of dose selection.

9.4. Sample Size

The treatment estimates are based on the study ACTT-1 of Remdesivir® for COVID-19. Under the TTCI definition used in this study, rate ratio (RR) for recovery for the severe patients, the patients with 5-point of baseline ordinal score and the patients with 6-point of baseline ordinal score is 1.31, 1.45 and 1.09 respectively[31, 32]. The event rate for the patients with 5-points is 88.8% and 76.8% for Remdesivir® group and placebo group, respectively. The assumption to calculate the sample size is set on the results from 5-points patients, which is RR as 1.4 and event rate as 80%. The sample size determination assumptions aim at a more meaningful clinical difference under a stricter TTCI definition.

Sample size is calculated based on the following assumptions:

- Overall Type-I error rate should not exceed 2.5% (1-sided);
- Rate ratio (RR) sets to be 1.4 (if the median TTCI is 9 days on the placebo group, the SCTA01 group will be 6.5 days under the assumption of Exponential distribution);
- Randomization ratio of study drug group and placebo group in Phase III is 1:1;
- Randomization ratio of medium dose group, high dose group and placebo group in Phase II is 1:1:1;
- 80% event rate;
- Sample size ratio of Phase II and Phase III is 1:1.79;
- Simes method will be applied to attain adjusted *p*-value from Phase II;

- Inverse normal weighting method will be used to combine *p*-value from Phase II and Phase III part;
- The 3rd interim analysis, which is the penultimate analysis for superior efficacy stopping and futility analysis, will be conducted when 31% of the TTCI events have been observed from the Phase III part;
- Pocock boundary (Lan DeMets 1983) will be used to control overall Type I error within 1-sided 2.5%;
- The critical values of nominal *p*-value will be re-calculated based on the actual number of TTCI events documented at the time of interim analysis and final analysis.

The study will randomize 795 patients totally, with Phase II of 285 patients and Phase III of 510 patients (255 in each group), providing over 90% power.

9.5. Baseline Descriptive Statistics

Baseline characteristics will be summarized by treatment arm. For continuous measures, the mean and standard deviation will be used. Categorical variables will be described by the proportion in each category (with the corresponding sample size numbers).

9.6. Safety Analysis

The general safety summaries will include treatment group and placebo group, comprising all patients in the safety set.

Adverse events will be tabulated by treatment group. The number and percentage of patients with at least one AE will be presented along with a breakdown by system organ class (SOC) and Preferred Term (PT), with each patient being counted once at each summarization level. This summary will be repeated for treatment-related AEs, AEs that lead to study drug discontinuation, by relationship to study drug, by intensity and for SAEs.

Laboratory test results, vital signs data, and ECG data will be presented in tables summarizing absolute values and change from baseline by treatment group, with shift tables included as appropriate.

9.7. Efficacy Analysis

9.7.1. Primary Efficacy Analysis

The primary endpoint is TTCI up to Day 29. TTCI is defined as the time (in days) from randomization to the first day on which a patient satisfies point 1, 2, or 3 on the 8-point ordinal scale and maintains a score ≤ 3 at least 48 hours (initial improvement) and maintains this up to Day 29 (sustained improvement).

All deaths within 29 days or terminated early due to AE will be designated censored at Day 29 with respect to primary endpoint. Any subjects lost to follow-up or terminated early due to other reasons prior to an observed primary endpoint will be censored at the day of their last observed assessment. Subjects who completed follow-up but did not experience a primary endpoint event will be censored at the day of their Day 29 visit. The subjects who take prohibited medications will be regarded as treatment failures and will be censored at the time of medication use.

The primary analysis will use the stratified log rank test to compare treatment group to control group through Day 29 with respect to primary endpoint. Covariate factors may include country, duration of symptoms prior to enrollment, comorbidity and best supportive treatment. Note that if some strata are too small, they might be combined together to allow statistical method work well.

For each group, the Kaplan-Meier estimate of TTCI survival function will be constructed. Hazard ratio of SCTA01 *vs.* placebo with two-sided 95% CI will be derived from the stratified Cox proportional hazard model. The number of patients with a TTCI event and number of patients censored for the TTCI analysis will be summarized by treatment group.

The sensitivity analyses (such as using different population, different censoring rules and/or different covariates) for the primary endpoint will be fully described in the statistical analysis plan (SAP).

9.7.2. Multiple Testing in Adaptive Seamless Design

9.7.2.1 Combination Test

Confirmatory testing of the hypothesis comparing optimal dose with placebo using data collected from Phase II and Phase III part will use a closed testing procedure. This hypothesis will be rejected at level α (one-side 0.025) if it and all intersection hypotheses involving it are all rejected at local level α (one-side 0.025). The testing strategy used to combine results from Phase II and Phase III part will be a combination test based on the inverse normal weighting method, with the test statistic of the combination test calculated as (*Bauer and Kohne 1994*):

$$\mathrm{Sqrt}(n_1/n)*\Phi^{-1}(1-p_1) + \mathrm{Sqrt}(n_2/n)*\Phi^{-1}(1-p_2)$$

Where n_1 and n_2 are the sample size in Phase II and III, respectively, $n = n_1+n_2$, Φ refers to the standard normal distribution, and p_1 and p_2 are the *p*-value from Phase II and III, respectively. p_1 is the Simes-adjusted *p*-value in Phase II, and p_2 is the unadjusted *p*-value to compare optimal dose with placebo in Phase III.

9.7.2.2 Adaptive Combination Test

We will control family-wise error rate at a nominal one-sided α of 0.025, which is the probability of rejecting at least one true null hypothesis. The general principle of "adaptive combination test" will be adhered to. Independence of the test statistics from Phase II and Phase III is achieved by fixing the follow-up time to obtain events required for the efficacy analysis for each Phase, thereby ensuring control of the Type I error via the closure principle.

To determine the efficacy boundary for the interim analysis, the critical values of the Pocock boundary is calculated using the sample sizes in the Phase III part alone. This then provides a conservative boundary to control the family-wise Type I error rate better than 2.5% when these boundaries are compared to the *p*-values derived from combining the *p*-values from the Phase II and Phase III parts. The boundaries are conservative when the same weights are used for both the interim and final *p*-values because when the same weights are used, the correlation between the *p*-values at interim and final is stronger when the *p*-values are first combined with the same data from phase II than when they are not combined with the Phase II data. Therefore, the adjustment of the final alpha required to control the Type 1 error overall would be smaller and the process is guaranteed to protect against Type I error inflation. This conclusion is supported by simulation results demonstrating that under the null hypothesis, the Type I error rate is less than 2.5%[33-36].

9.7.3. Analysis of Secondary Endpoints

Differences in time-to-event endpoints by treatment will be summarized with Kaplan-Meier curves and 95% confidence bounds.

Proportions will be used to summarize changes in ordinal scale at specific time points.

Duration of event will be determined according to median days with quartiles.

Binary data summarized as a percent with 95% confidence intervals. Comparisons between arms presented as differences in proportions with 95% confidence intervals.

Categorical data may be summarized according to proportions by category and/or odds ratios with confidence intervals.

9.7.4. IDMC

This study will use an independent data monitoring committee (IDMC).

The IDMC will monitor the safety of the SCTA01 during the trial on regular basis. The frequency and criteria to trigger the IDMC review is specified in the IDMC Charter.

The IDMC will be responsible for recommending the dose for Phase III part. The recommendations made by the IDMC to alter the conduct of the study will be forwarded to SCT for final decision. SCT will forward such decisions, which may include summaries of aggregate analyses of endpoint events and of safety data that are not endpoints, to regulatory authorities, as appropriate.

9.7.5. Interim Analyses

There will be three un-blinded interim analyses: the first IA is when the 143rd patient in Phase II has completed Day 8 visit; the second IA is at the end of Phase II; the third IA is when the 160th patient in Phase III has completed Day 29 or Early Withdraw visit. The timing of the third IA will be driven by the number of events accumulated in the Phase III part of the study, which is equivalent to about 31% of total number of events required in the Phase III part of the study.

Phase II: Two unblinded interim analyses are planned when the 143rd patient in Phase II has completed Day 8 visit. The preliminary review of efficacy data is supported if early data and external emerging data warrants. Given the event rate of 80%, 143 subjects would provide 80% power to detect a RR of 1.4 for a favorable outcome on TTCI for SCTA01 over placebo using a one-sided p-value of 0.2. Recommendations from the IDMC of no chance of success may be made but is not binding due to limited sample size. The 2nd interim analysis will be performed at the end of Phase II when the 285th patient has completed Day 29 or Early Withdraw visit. The purposes of this interim analysis are to select the dose for Phase III part of the study and check the futility of the study. The dose selected to continue to Phase III part will be the dose with a greater effect in reduction of viral load compared to placebo at Day 8 with no harmful effect on clinical improvement. Some other threshold, e.g. RR <1.2, may be used as a futility boundary at the end of Phase II. This more stringent than calculated futility boundary is set to control unwanted false positive rate. By applying calculated futility boundary from the design, the probability to claiming superiority of one dose in the end of trial is greater than 90% from the simulation. Considering the entirety of the data, if the IDMC observes an effect on viral load, study continuation may be recommended by the IDMC even if the futility bound is met on TTCI.

The stopping boundaries for efficacy and futility is displayed in the following table along with the stopping probabilities from simulation studies under assumptions for the effect of SCTA01 being harmful (RR = 0.9) or having no effect (RR = 1).

Table 7. Stopping Boundaries for Efficacy and Futility

	**Events**	**Efficacy boundary (*p*-value)**	**Futility boundary**	**Cumulative probability of stopping under RR = 0.9**	**Cumulative probability of stopping under RR = 1**
Interim 1(Safety review only)	NA	NA	NA	NA	NA
End of Phase II	149	NA	RR < 1.15	0.9385	0.8106
Interim 3	280	0.01068	RR < 1.22	0.9987	0.985
End of Phase III	556	0.0166	NA	0.9999	0.9897

Our simulation results suggest with the planned sample size and timing of interim analysis, under the hypothesis that SCTA01 is harmful (RR = 0.9) or has no effect (RR = 1), the probability of stopping the trial for applying a futility boundary of RR=1.15 is very probable with at least 81% probability, even at the early enrollment of the trial, e.g. the end of Phase II. Similarly, the probability of stopping the trial for applying a futility boundary of RR=1.2 is very probable with at least 88% probability.

Phase III: An unblinded interim analysis is planned.

The penultimate analysis, that allows for stopping the study for superior efficacy is planned when approximately 128 (31%) of the TTCI events have been documented in the Phase III ITT population. An α-spending function due to Lan DeMets (1983) with Pocock type stopping boundary will be used for the interim efficacy analysis. Therefore, if the interim analysis is done right after 280 events from Phase II and Phase III part have been documented, the combined *p*-value based on the inverse normal method of less than **1-side of 0.01068** will need to be observed to declare statistical significance. A futility boundary of an observed RR of 1.22 may be used to declare failure of the study when TTCI events from Phase II and Phase III part combined are exactly 280. Similarly, this more stringent than calculated futility boundary is set to control unwanted false positive rate. Note that the critical values of nominal *p*-value and hazard ratio needed to declare statistical significance at the interim analysis will be calculated based on the actual number of TTCI events documented at the time of the interim analysis.

An independent statistician external to the study team will perform the interim analyses and present the results to the IDMC. The IDMC will provide the Sponsor with a recommendation to continue the trial as planned, to have an interruption, to make a protocol amendment, or to terminate the trial. If the interim analysis demonstrates significant shorten TTCI in SCTA01 group compared to control group, the IDMC could recommend to terminate and un-blinded the study, and thus announce the study as completed.

9.7.6. Final Analysis

Final analysis will be performed when 556 TTCI events have been observed. A combined *p*-value based on the inverse normal weighting method of less than **1-side of 0.0166** will need to be

observed to declare statistical significance. The above derived boundaries are based on the assumption that the final analysis is performed when exactly 556 events are observed from both Phase II and Phase III part. If this assumption does not hold, the boundaries for p-value will be re-calculated according to the aforementioned α-spending function.

9.7.7. Sub-group Analyses

Subgroup analyses for the primary outcomes will evaluate the treatment effect across the following subgroups: country, race, ethnic group, duration of symptoms prior to enrollment, age, sex and comorbidity, best supportive treatment. A forest plot will display confidence intervals across subgroups. Interaction tests will be conducted to determine whether the effect of treatment varies by subgroups.

9.8. Group Sequential Multiplicity Adjustment

The interim analysis for superior efficacy stopping will be conducted when 31% of the planned TTCI events in the Phase III study have been observed. Pocock boundary (Lan DeMets 1983) will be used to control overall Type I error within 2-side 5%.

9.9. Handling of Missing Data

For analysis of categorical secondary efficacy variables, missing data will be imputed using an NRI method (the participants with missing data will be considered non-responders). For time to event endpoints, different censoring rules will be detailed for each different endpoint respectively.

Details of the handling of dropouts or missing data will be fully described in the statistical analysis plan (SAP).

9.10.Pharmacokinetics and Immunogenicity Analysis

Individual SCTA01 concentrations will be tabulated using descriptive statistics. Pharmacokinetic characteristics including (but not limited to) area under the curve (AUC), maximum concentration (C_{max}), peak time (T_{max}), will be derived using PopPK analysis.

Base on the PK data obtained in the Phase II as well as PK data obtained from other studies (if available), a population PK data analysis may be conducted to determine the mean concentration versus time profiles and covariates (to be detailed in a separate analysis plan).

The immunogenic potential of SCTA01 will be assessed by summarizing the number and percentage of subjects who develop detectable ADA. The impact of ADA on PK will be assessed if data allow.

10. REGULATORY CONSIDERATIONS

10.1.Good Clinical Practice

The procedure set out in this study protocol pertaining to the conduct, evaluation, and documentation of this study are designed to ensure that the Sponsor, its authorized representative(s), and Investigator(s) abide by Good Clinical Practice (GCP), as described in the International Council for Harmonisation or Technical Requirements for Pharmaceuticals for Human Use (ICH) Guideline E6 and in accordance with the general ethical principles outlined in the Declaration of Helsinki. The study will receive approval from an IRB/IEC prior to commencement. The Investigator will conduct all aspects of this study in accordance with applicable national, state, and local laws of the pertinent regulatory authorities.

10.2.Investigator Responsibilities

Investigator responsibilities are set out in the ICH Guideline for Good Clinical Practice and in the local regulations. Sponsor staff or an authorized representative will evaluate and approve all Investigators who in turn will select their staff.

The Investigator should ensure that all persons assisting with the study are well informed about the protocol, amendments, study treatments, as well as study-related duties and functions. The Investigator should maintain a list of Sub-Investigators and other appropriately qualified persons to whom he or she has delegated significant study-related duties.

The Investigator is responsible for keeping a record of all subjects who sign an informed consent document and are screened for entry into the study. Subjects who fail screening must have the reason(s) recorded in the subject's source documents.

The Investigator, or a designated member of the Investigator's staff, must be available during monitoring visits to review data, resolve queries and allow direct access to subject records (e.g., medical records, office charts, hospital charts, and study-related charts) for source data verification. The Investigator must ensure timely and accurate completion of CRFs and queries.

10.3.Subject Information and Informed Consent

The Investigator must obtain informed consent from the subject or a legal representative prior to performing any study-related procedures. Each Investigator has both ethical and legal responsibility to ensure that the subjects being considered for inclusion in this study are provided with full explanation of the protocol and the roles and responsibilities of the subject for participation in the study.

Documentation that Informed Consent was obtained prior to the study subject's entry into the study and of the informed consent process should be recorded in the study subject's source documents, including the date. In addition, if a protocol is amended and the amendment(s) impact the content of the informed consent, the informed consent document must be revised. Study subjects participating in the study when the amended protocol is implemented must be re-consented with the revised version of the informed consent document. The revised informed consent document signed and dated by the study subject and as well as the person consenting the study subject must be maintained in the Investigator's study files and a copy given to the study subject.

10.4. Confidentiality

The sponsor affirms the subject’s right to protection against invasion of privacy and to be in compliance with the ICH and other local regulations (whichever is most stringent). The sponsor requires the Investigator to permit sponsor and/or their designated representatives and, when necessary, representatives from regulatory authorities, to review and/or copy any medical records relevant to the study in accordance with local laws.

Should direct access to medical records require a waiver or authorization separate from the subject’s signed informed consent document, it is the responsibility of the Investigator to obtain such permission in writing from the appropriate individual.

Only initials and unique subject numbers will be used to identify subjects in CRFs.

10.5. Protocol Amendment

Any amendment to protocol must be approved by the sponsor or its designated Clinical Research Physician/Medical Monitor. Amendments will subsequently be submitted to the IRB/IEC for written approval. Written approval must be obtained before implementation of the amended version occurs. The written signed approval from the IRB/IEC should specifically reference the Investigator name, protocol number, study title and amendment number(s) that is applicable. Amendments that are administrative in nature do not require IRB/IEC approval but will be submitted to the IRB/IEC for information purposes.

10.6. Institutional Review Board/Independent Ethics Committee Review and Approval

The final study protocol, including the final version of the ICF, must be approved or given a favorable opinion in writing by an IRB or IEC as appropriate. The investigator must submit written approval to the sponsor before he or she can enroll any subject into the study.

The Investigator is responsible for informing the IRB or IEC of any amendment to the protocol in accordance with local requirements. In addition, the IRB or IEC must approve all advertising used to recruit subjects for the study. The protocol must be re-approved by the IRB or IEC upon receipt of amendments and annually, as local regulations require.

The Investigator is also responsible for providing the IRB with reports of any reportable serious adverse drug reactions from any other study conducted with the investigational product. SCT will provide this information to the Investigator.

Progress reports and notifications of serious adverse drug reactions will be provided to the IRB or IEC according to local regulations and guidelines.

All materials approved by the IRB/IEC for this study, including the subject consent form and recruitment materials, must be maintained by the Investigator and made available for inspection.

10.7. Closure of Study

The sponsor reserves the right to terminate this study at any time for reasonable medical or administrative reasons. Any premature discontinuation will be appropriately documented according to local requirements (e.g., IRB/IEC, regulatory authorities).

In addition, the investigator or the sponsor has the right to discontinue a single site at any time during the study for medical or administrative reasons such as:

- Unsatisfactory enrollment；
- GCP noncompliance；
- Inaccurate or incomplete data collection；
- Falsification of records；
- Failure to adhere to the study protocol.

11. DATA HANDLING AND RECORD KEEPING

11.1.Data/Documents

The Investigator must ensure that the records and documents pertaining to the conduct of the study and the distribution of the investigational product are complete, accurate, filed, and retained. Examples of the source documents include hospital records, clinic and office charts, laboratory notes, memoranda, subject's diaries or evaluation checklists, dispensing records, recorded data from automated instruments, copies or transcriptions certified after verification as being accurate copies, microfiche, X-ray film and reports, and records kept at the pharmacy and the laboratories, as well as copies of CRFs or CD-ROM.

11.2.Data Management

Study data will be collected via an EDC system and will be managed through the use of programmed electronic edit checks. Data discrepancies will be brought to the attention of the clinical team and investigational site personnel, if necessary. An audit trail of the resolution of data discrepancies will be maintained in the EDC system.

11.3.Inspection of Records

Sponsor will be allowed to conduct periodic site visits to the investigation facilities for the purpose of monitoring any aspect of the study. The Investigator agrees to allow the monitor to inspect the drug storage area, study drug stocks, drug accountability records, subject charts and study source documents, and other records relative to study conduct.

11.4.Retention of Records

Essential documents must be retained by the Investigator for a minimum of 2 years after the last approval of a marketing application in an ICH region and until there are no pending or contemplated marketing applications in an ICH region, or at least 2 years have elapsed since the formal notification to regulatory authorities of discontinuation of clinical development of the study drug; and for a period of at least 3 years after the sponsor notifies the Investigator that the final report has been filed with regulatory authorities. The Investigator must retain these documents for the time period described above or according to local laws or requirements, whichever is longer. Essential documents include, but are not limited to, the following:

- Signed informed consent documents for all subjects;
- Subject identification code list, screening log (if applicable), and enrollment log;
- Record of all communications between the Investigator and the IRB/IEC;
- Composition of the IRB/IEC;
- Record of all communications among the investigator, sponsor, and their authorized representative(s);
- List of Sub-Investigators and other appropriately qualified persons to whom the Investigator has delegated significant study-related duties, together with their roles in the study, curriculum vitae, and their signatures;

- Copies of CRFs (if paper) and of documentation of corrections for all subjects;
- Study drug accountability records.

If it becomes necessary for the sponsor or the Regulatory Authority to review any documentation relating to the study, the Investigator must permit access to such records.

12. QUALITY CONTROL AND QUALITY ASSURANCE

The sponsor or its authorized representative(s) will carefully monitor all aspects of the study to ensure it is compliant with applicable government regulations in accordance with current GCP and standard operating procedures (SOP).

12.1.Study Monitoring

Before an investigational site can enter a subject into the study, a representative of the sponsor will visit the investigational study site to:

- Determine the adequacy of the facilities;
- Discuss with the Investigator(s) and other personnel their responsibilities with regard to protocol adherence and the responsibilities of sponsor or its representatives. This will be documented in a Clinical Study Agreement between sponsor and the Investigator.

During the study, a monitor from sponsor or representative will have regular contacts with the investigational site, for the following:

- Provide information and support to the Investigator(s);
- Confirm that facilities remain acceptable;
- Confirm that the investigational team is adhering to the protocol, data are being accurately recorded in the case report forms, and investigational product accountability checks are being performed;
- Perform source data verification. This includes a comparison of the data in the case report forms with the subject's medical records at the hospital or practice and other records relevant to the study. This will require direct access to all original records for each subject (e.g., clinic charts);
- Record and report any protocol deviations not previously sent to sponsor;
- Confirm AEs and SAEs have been properly documented on CRFs and confirm any SAEs have been forwarded to sponsor and those SAEs that met criteria for reporting have been forwarded to the IRB.

The monitor will be available between visits if the Investigator(s) or other staff needs information or advice.

12.2.Audits and Inspections

Authorized representatives of sponsor, a regulatory authority, or an IRB/IEC may visit the site to perform audits or inspections, including source data verification. The purpose of a sponsor audit or inspection is to conduct a systematic and independent examination of all study-related activities and documents to determine whether these activities were conducted, and data were recorded, analyzed, and accurately reported according to the protocol, GCP guidelines of the ICH, and any applicable regulatory requirements. The Investigator is required to permit direct access to the facilities where the study took place, source documents, CRFs, and applicable supporting records of study subject participation for audits and inspections by company authorized representatives,

regulatory authorities, and IRB/IECs. The Investigator should make every effort to be available for the audits and/or inspections. The Investigator should contact sponsor immediately if contacted by a regulatory agency about an inspection.

13. PUBLICATION POLICY

The results of this study may be published in a medical journal, presented at a medical congress, or used for teaching purposes. Additionally, this study and its results may be submitted for inclusion in all appropriate health authority study registries, as well as publication on health authority study registry websites, as required by local health authority regulations.

Authorship will be based on the International Committee of Medical Journal Editors criteria for authorship:

1. Substantial contributions to the conception or design of the work; or the acquisition, analysis, or interpretation of data for the work; AND
2. Drafting the work or revising it critically for important intellectual content; AND
3. Final approval of the version to be published/presented; AND
4. Agreement to be accountable for all aspects of the work in ensuring that questions related to the accuracy or integrity of any part of the work are appropriately investigated and resolved.

All four conditions above must be met to qualify for authorship.

Sponsor must be informed of any plans to publish the results of this study and has the right to review any publication (e.g., manuscript, abstract, oral/slide presentation, book chapter) resulting from this study before it is submitted/presented.

14. REFERENCES

1. Beigel JH, Tomashek KM, Dodd LE, et al. Remdesivir for the Treatment of Covid-19 Preliminary Report. This article was published on May 22, 2020, at NEJM.org.
2. Johns Hopkins University. Available from https://coronavirus.jhu.edu/map.html.
3. https://www.accessdata.fda.gov/scripts/cder/daf/index.cfm?event=BasicSearch.process
4. Thangaraju P, Venkatesan N, Thangaraju E, et al. Are We Jumping the Gun with Itolizumab in India? A Situational Analysis from the Pre-COVID Era. SN Compr Clin Med. 2020 Sep 26: 1- 6.
5. https://www.fda.gov/media/143602/download.
6. https://www.fda.gov/media/143891/download.
7. Wrapp D, Wang N, Corbett KS, Goldsmith JA, Hsieh CL, Abiona O, et al. Cryo-EM structure of the 2019-nCoV spike in the prefusion conformation. Science 2020; 367:1260-3.
8. Siddiqi HK, Mehra MR. COVID-19 illness in native and immunosuppressed states: A clinical-therapeutic staging proposal. J Heart Lung Transplant 2020; 39:405-7.
9. Chinese clinical guidance for COVID-19 pneumonia diagnosis and treatment. Seventh Edition. 2020.] Available from http://kjfy.meetingchina.org/msite/news/show/cn/3337.html.
10. Shang L, Zhao J, Hu Y, Du R, Cao B. On the use of corticosteroids for 2019-nCoV pneumonia. Lancet 2020; 395:683-4.
11. Shen C, Wang Z, Zhao F, Yang Y, Li J, Yuan J, et al. Treatment of 5 Critically Ill Patients With COVID-19 With Convalescent Plasma. JAMA 2020; 323:1582-9.
12. Duan K, Liu B, Li C, Zhang H, Yu T, Qu J, et al. Effectiveness of convalescent plasma therapy in severe COVID-19 patients. Proc Natl Acad Sci U S A 2020; 117:9490-6.
13. Ye M, Fu D, Ren Y, Wang F, Wang D, Zhang F, et al. Treatment with convalescent plasma for COVID-19 patients in Wuhan, China. J Med Virol 2020.
14. Zhang B, Liu S, Tan T, Huang W, Dong Y, Chen L, et al. Treatment With Convalescent Plasma for Critically Ill Patients With Severe Acute Respiratory Syndrome Coronavirus 2 Infection. Chest 2020; 158:e9-e13.
15. Olivares-Gazca JC, Priesca-Marin JM, Ojeda-Laguna M, Garces-Eisele J, Soto-Olvera S, Palacios-Alonso A, et al. Infusion of Convalescent Plasma Is Associated with Clinical Improvement in Critically Ill Patients with Covid-19: A Pilot Study. Rev Invest Clin 2020; 72:159-64.
16. Salazar E, Perez KK, Ashraf M, Chen J, Castillo B, Christensen PA, et al. Treatment of COVID-19 Patients with Convalescent Plasma in Houston, Texas. medRxiv 2020.
17. Salazar E, Christensen PA, Graviss EA, et al. Treatment of COVID-19 Patients with Convalescent Plasma Reveals a Signal of Significantly Decreased Mortality. Am J Pathol. 2020 Aug 11; S0002-9440(20)30370-9. doi: 10.1016/j.ajpath.2020.08.001. Online ahead of print.
18. Emergency Use Authorization: Emergency Use Authorization (EUA) information, and list of

all current EUAs. https://www.fda.gov/emergency-preparedness-and-response/mcm-legal-regulatory-and-policy-framework/emergency-use-authorization#coviddrugs, last accessed 9/4/2020.

19. Guidance for Industry: Estimating the Maximum Safe Starting Dose in Initial Clinical Trials for Therapeutics in Adult Healthy Volunteers. Center for Drug Evaluation and Research (CDER),FDA, July 2005. https://www.fda.gov/media/72309/download.
20. Bao L, Deng W, Huang B, Gao H, Liu J, Ren L, et al. The pathogenicity of SARS-CoV-2 in hACE2 transgenic mice. Nature 2020; 583:830-3.
21. Puelles VG, Lutgehetmann M, Lindenmeyer MT, Sperhake JP, Wong MN, Allweiss L, et al. Multiorgan and Renal Tropism of SARS-CoV-2. N Engl J Med 2020; 383:590-2.
22. Gan R, Rosoman NP, Henshaw DJE, Noble EP, Georgius P, Sommerfeld N. COVID-19 as a viral functional ACE2 deficiency disorder with ACE2 related multi-organ disease. Med Hypotheses 2020; 144:110024.
23. Zheng S, Fan J, Yu F, Feng B, Lou B, Zou Q, et al. Viral load dynamics and disease severity in patients infected with SARS-CoV-2 in Zhejiang province, China, January-March 2020: retrospective cohort study. Bmj 2020; 369:m1443.
24. Liu Y, Yan LM, Wan L, Xiang TX, Le A, Liu JM, et al. Viral dynamics in mild and severe cases of COVID-19. Lancet Infect Dis 2020; 20:656-7.
25. Magleby R, Westblade LF, Trzebucki A, Simon MS, Rajan M, Park J, et al. Impact of SARS-CoV-2 Viral Load on Risk of Intubation and Mortality Among Hospitalized Patients with Coronavirus Disease 2019. Clin Infect Dis 2020.
26. To KK, Tsang OT, Leung WS, Tam AR, Wu TC, Lung DC, et al. Temporal profiles of viral load in posterior oropharyngeal saliva samples and serum antibody responses during infection by SARS-CoV-2: an observational cohort study. Lancet Infect Dis 2020; 20:565-74.
27. Peter Chen, Ajay Nirula, Barry Heller, et al. SARS-CoV-2 Neutralizing Antibody. LY-CoV555 in Outpatients with Covid-19. NEJM, 2020. DOI: 10.1056/NEJMoa2029849.
28. D.M. Weinreich, S. Sivapalasingam, T. Norton, et al. REGN-COV2, a Neutralizing Antibody Cocktail, in Outpatients with Covid-19. NEJM, 2020. DOI: 10.1056/NEJMoa2035002.
29. Sampson HA., et al., Second symposium on the definition and management of anaphylaxis: summary report—second National Institute of Allergy and Infectious Disease/Food Allergy and Anaphylaxis Network symposium. Ann Emerg Med, 2006. 47(4): p. 373-380.
30. Riviello ED, Kiviri W, Twagirumugabe T, Mueller A, Banner-Goodspeed VM, Officer L, et al. Hospital Incidence and Outcomes of the Acute Respiratory Distress Syndrome Using the Kigali Modification of the Berlin Definition. Am J Respir Crit Care Med. 2016; 193(1):52-9. Epub 2015/09/10.
31. J.H. Beigel, K.M. Tomashek, L.E. Dodd, et al. Remdesivir for the Treatment of Covid-19 — Final Report. The New England Journal of Medicine. 2020.
32. https://www.accessdata.fda.gov/drugsatfda_docs/nda/2020/214787Orig1s000Sumr.pdf.
33. Bretz F, Schmidli H, Konig F, Racine A, and Maurer W. Confirmatory seamless phase II/III

clinical trials with hypothesis selection at interim: general concepts. Biometrical Journal 48 (2006) 4:623-634.

34. Stallard, N. (2010). A confirmatory seamless phase II/III clinical trial design incorporating short-term endpoint information. Statistics in Medicine, 29, 959-971.

35. Stallard, Nigel. (2011). Group-Sequential Methods for Adaptive Seamless Phase II/III Clinical Trials. Journal of biopharmaceutical statistics. 21. 787-801.

36. Lan KKG, DeMets DL. Discrete sequential boundaries for clinical trials. Biometrika. 1983; 70:659-663.

APPENDIX 1. INVESTIGATOR'S PROTOCOL AGREEMENT

**Study Title:**	A multicenter, adaptive, randomized, double-blinded, placebo-controlled Phase II/III trial to evaluate the efficacy and safety of monoclonal antibody SCTA01 against SARS-CoV-2 in hospitalized patients with severe COVID-19
**Study Number:**	SCTA01-B301
**Final Date:**	24 August 2021

I confirm that my staff and I have carefully read and understand this protocol. I/we agree to comply with the procedures and terms of the study specified herein. In particular, I/we have agreed to:

- Abide by all obligations stated on Form FDA 1572 and on other document(s) required by local regulatory authority；
- Retain records and documents related to this trial for at least 2 years after the last approval of a marketing application in an ICH region and until there are no pending or contemplated marketing applications in an ICH region or at least 2 years have elapsed since the formal discontinuation of clinical development of the investigational product；
- Comply with GCP and all applicable regulatory requirements；
- Maintain confidentiality and assure security of sponsor confidential documents；
- Obtain Institutional Review Board (IRB) approval of the protocol, any amendments to the protocol, and periodic re-approval as required, and to keep the IRB informed of adverse events and periodically report the status of the study to them；
- Not implement any deviations from or changes to the protocol without agreement from the sponsor and prior review and written approval from the IRB, except where necessary to eliminate an immediate hazard to the subjects or for administrative aspects of the study (where permitted by all applicable regulatory requirements)；
- Assure that each subject enrolled into the trial has read, understands, and has signed the Informed Consent；
- Ensure that I and all persons assisting me with the study are adequately informed and trained about the investigational drug and of their study-related duties and functions as described in the protocol；
- Make prompt reports of SAEs and deaths (within 24 hours of learning of the SAE or death) to the sponsor；
- Assure access by sponsor monitors and/or FDA to original source documents；
- Prepare and maintain adequate and accurate case histories designed to record all observations and other data pertinent to the investigation on each individual treated in the investigation；

- Arrange for the transfer of appropriate data from case histories to case report forms for the collection and transmission of data to the sponsor;
- Cooperate fully with any study-related GCP audit as performed by the sponsor quality assurance group specified by the sponsor;
- Abide by the stipulations in the Disclosure of Data section and the manuscript.

_____

Printed Name of Investigator

_____

Signature of Investigator

___

Date

APPENDIX 2. SPONSOR SIGNATURE

**Study Title:**	A multicenter, adaptive, randomized, double-blinded, placebo-controlled Phase II/III trial to evaluate the efficacy and safety of monoclonal antibody SCTA01 against SARS-CoV-2 in hospitalized patients with severe COVID-19
**Study Number:**	SCTA01-B301
**Final Date:**	24 August 2021

This clinical study protocol was subjected to critical review and has been approved by the Sponsor. The following personnel contributed to writing and/or approving this protocol:

Signed: ____________________________ Date: ______________
