# Supplementary material for: Fc-Modified Antibody in Hospitalized Severe COVID-19 Patients
Source: Vaccines (Basel). 2025 Mar 31;13(4):372. doi: 10.3390/vaccines13040372 (PMC12031629; doi:10.3390/vaccines13040372)
Supplement: Supplementary file 1 [file vaccines-13-00372-s001.zip › Supplement 2.pdf]

# Statistical Analysis Plan

# A Multicenter, Adaptive, Randomized, Double-blinded, Placebo-controlled Phase II/III Trial to Evaluate the Efficacy and Safety of Monoclonal Antibody SCTA01 against SARS-CoV-2 in Hospitalized Patients with Severe COVID-19

## Sinocelltech, Ltd.

## SCTA01-B301

Document Version: V 1.0

Document Date: 25 April 2021

# Table of Contents

Table of Contents ........ 2
Glossary of Abbreviations ........ 4
1. Source Documents ........ 5
2. Protocol Details ........ 5
2.1 Study Objectives ........ 5
2.1.1. Primary Objectives ........ 5
2.1.2. Secondary Objectives ........ 5
2.1.3. Exploratory Objectives ........ 5
2.2 Overall Study Design ........ 5
2.3 Sample Size and Power ........ 7
3. Efficacy and Safety Variables ........ 8
3.1 Primary Efficacy Endpoint ........ 8
3.2 Secondary Efficacy Endpoints ........ 8
3.3 Safety Variables ........ 10
4. Pharmacokinetic/Pharmacodynamic variables ........ 11
5. Analysis populations ........ 11
5.1 All Enrolled Subjects Set ........ 11
5.2 Safety Set (SS) ........ 11
5.3 Intent-to-treat (ITT) Set ........ 11
5.4 Full Analysis Set (FAS) ........ 11
5.5 Per Protocol Set (PPS) ........ 11
5.5.1 Major Protocol Deviations Leading to Exclusion from the PPS Analysis ........ 12
5.6 Pharmacokinetics set (PKS) ........ 12
5.7 Seronegative ITT ........ 12
5.8 Seropositive ITT ........ 12
6. DATA Handling ........ 12
6.1 Time points and Visit Windows ........ 12
6.2 Baseline definition ........ 13
6.3 Handling of Dropouts, Missing Data, and Outliers ........ 13
7. Statistical Methods ........ 14

7.1 General Principles ........ 14
7.2 Subject Disposition and Data Sets Analyzed ........ 15
7.3 Protocol Deviations ........ 15
7.4 Baseline Disease Characteristics ........ 16
7.5 Demographics and Other Baseline Characteristics ........ 16
7.5.1 Medical History and Prior and Concomitant Medication ........ 17
7.6 Measurements of Treatment Compliance ........ 18
7.7 Efficacy ........ 18
7.7.1 Statistical Hypothesis: ........ 18
7.7.2 Primary Efficacy Analysis ........ 18
7.7.3 Secondary Efficacy Analysis ........ 21
7.7.4 Sensitivity Analysis ........ 27
7.7.5 Subgroup Analysis ........ 28
7.7.6 Exploratory Analysis ........ 28
7.7.7 Multiplicity Adjustment ........ 29
7.8 Safety ........ 30
7.8.1 Extent of Exposure ........ 30
7.8.2 Adverse Events ........ 30
7.8.3 Laboratory Evaluations ........ 33
7.8.4 Vital Signs and $SpO_2$ ........ 35
7.8.5 Electrocardiograms ........ 36
7.8.6 Physical Examination ........ 37
7.8.8 Other Safety Variables ........ 37
7.9 Pharmacokinetics Analysis ........ 37
7.10 Immunogenicity Analysis ........ 38
7.11 Interim Analysis ........ 38
8. Changes from Protocol ........ 38
9. Data Issues ........ 39
10. References ........ 40
11. Appendices ........ 42

# Glossary of Abbreviations

| Abbreviation | Term |
|---|---|
| AE | Adverse Event |
| ANCOVA | Analysis of covariance |
| ANOVA | Analysis of variance |
| AUC | area under the plasma concentration versus time curve |
| $AUC_{0-t}$ | area under the plasma concentration versus time curve from time zero to the time of last quantifiable concentration |
| $AUC_{0-\infty}$ | area under the plasma concentration versus time curve from time zero to infinity |
| BMI | Body Mass Index |
| BLQ | below the level of quantification |
| BSC | Best supportive care |
| CI | Confidence Interval |
| CL | Clearance |
| $C_{max}$ | maximum plasma concentration |
| COVID-19 | Coronavirus Disease 2019 |
| DMC | Data Monitoring Committee |
| ECG | Electrocardiogram |
| ECMO | extracorporeal membrane oxygenation |
| ET | Early Termination |
| eCRF | Electronic Case report form |
| ICF | Informed Consent Form |
| ICU | intensive care unit |
| IDMC | Independent data monitoring committee |
| ITT | intention-to-treat |
| LLOQ | the lower limit of quantification |
| KM | Kaplan-Meier |
| MedDRA | Medical Dictionary for Regulatory Activities |
| NC | not calculated |
| NP | Nasopharyngeal |
| PK | Phamacokinetics |
| PKS | Pharmacokinetic Set |
| PPS | Per Protocol Set |
| PT | Preferred Term |
| QTcB | Bazett corrected QT interval |
| QTcF | Fridericia corrected QT interval |
| SAE | Serious Adverse Event |
| SAP | Statistical Analysis Plan |
| SD | Standard Deviation |
| SOC | System Organ Class |
| SoC | Standard of Care |
| SS | Safety Set |
| TEAE | Treatment Emergent Adverse Event |
| TFLs | Tables, Figures and Listings |
| $T_{max}$ | Time to $C_{max}$ |
| TTCI | Time to clinical improvement |
| $T_{1/2}$ | Half-life time |
| Vd | Apparent Volume of Distribution |

# 1. Source Documents

The Statistical Analysis Plan was written based on the following documentation:

| Document | Date | Version |
|---|---|---|
| Protocol | 24 Aug 2021 | V5.0 |
| CRF | 2 March 2021 | V1.0 |

# 2. Protocol Details

## 2.1 Study Objectives

### 2.1.1. Primary Objectives

- To evaluate the clinical efficacy of SCTA01 as assessed by time to clinical improvement (Phase II, III).

### 2.1.2. Secondary Objectives

- To evaluate the clinical efficacy of SCTA01 as assessed by clinical severity, hospitalization, mortality (Phase II, III);
- To evaluate the safety of SCTA01 (Phase II, III);
- To evaluate the virologic efficacy of SCTA01 (Phase II, III);
- To evaluate the pharmacokinetics of SCTA01 (Phase II);
- To evaluate the immunogenicity of SCTA01 (Phase II, III).

### 2.1.3. Exploratory Objectives

- To explore the correlation between the baseline serological antibody level and therapeutic effect (Phase II, III);
- To evaluate the virologic efficacy of SCTA01 as assessed by quantitative SARS-CoV-2 virus in patient's blood (Phase II, III).

## 2.2 Overall Study Design

The study is a multicenter, adaptive, randomized, double-blinded and placebo-controlled Phase II/III trial, and will be conducted at selected investigational sites globally.

Phase II part will evaluate the efficacy, safety, and PK of SCTA01 15 mg/kg, 50 mg/kg in patients with severe COVID-19. The primary endpoint will evaluate TTCI in study group 1 [SCTA01 15 mg/kg + best supportive care (BSC)], study group 2 (SCTA01 50 mg/kg + BSC) and control group (placebo + BSC) up to Day 29. The secondary endpoint will evaluate the virologic efficacy in study group 1 (SCTA01 15 mg/kg

+BSC), study group 2 (SCTA01 50 mg/kg + BSC) and the control group (placebo + BSC) from baseline to Day 8, and then determine the recommended dose for Phase III part.

In the Phase II part of the study, 285 subjects will be randomized by 1:1:1 ratio to 15 mg/kg group, 50 mg/kg group and placebo group, with 95 subjects in each group.

At the end of Phase II part, enrollment will be paused and an interim analysis for dose selection will be performed after the 285th subject has been observed for TTCI. An IDMC will assess the benefit and risk to decide the dose for Phase III part of the part.

Enrollment will be resumed and the study continue into Phase III part after the IDMC has completed the evaluation and recommended the dose for Phase III part.

The Phase III part will evaluate the efficacy, safety, and immunogenicity of SCTA01 at the dosage recommended by the IDMC (X mg/kg or X mg) in severe patients with COVID-19. The primary endpoint will evaluate TTCI (8-point ordinal scale) in study group (SCTA01 X mg/kg or X mg + BSC) and control group (placebo + BSC) up to Day 29.

Approximately 510 subjects in Phase III part will be randomized by 1:1 ratio to SCTA01 (X mg/kg or X mg) and placebo, with 255 subjects in each group. The IDMC will be used to monitor safety and efficacy information.

Total number of subjects in the study is about 795. The final analysis will include subjects enrolled in the same treatment or placebo group in phase 2 and phase 3

Interim analysis (IA): There will be three un-blinded interim analyses when 143rd patient in Phase II has completed Day 8 visit, at the end of Phase II and when the 160th patient in Phase III has completed Day 29 or Early Withdraw visit.

- The 1st interim analysis is for safety evaluation. The safety data will be reviewed at the 1st interim analysis, but the preliminary review of efficacy data is supported if early data and external emerging data warrants.
- The 2nd interim analysis is for dose selection: the analysis will be performed at the end of Phase II when the 285th patient completed Day 29 or Early Withdraw visit. The dose selected to continue to the Phase III part will be the dose with the greater effect in reduction of viral load compared to placebo at Day 8. Time to clinical improvement of each dose will be evaluated and the RR threshold of 1.15 will be used as a futility boundary at the end of Phase II. The futility boundary is non-binding, and the IDMC may recommend study continuation even if the futility bound is met on TTCI. If any safety signal is observed for any dose, the IDMC

will weigh this information against the efficacy results in selecting the dose for Phase III.

- The 3rd interim analysis is the penultimate analysis that allows for stopping for superior efficacy. It is planned when 160 patients in Phase III (approximately 31% information time of Phase III part, which will have approximately 128 TTCI events) have been documented in the ITT population. If the interim analysis is performed at 31% of information time of Phase III, based on Pocock type boundary of α-spending function, a 1-sided *p*-value less than 0.01068 using inverse normal method to combine *p*-value from Phase II part and Phase III part will need to be observed to declare statistical significance. A futility boundary of an observed RR of 1.22 will be used to declare failure of the study when TTCI events from Phase II and Phase III part combined are exactly 280, which is equivalent to an observed one-sided p-value of 0.05.

Note: The critical values of nominal *p*-value and rate ratio that will be used to declare statistical significance and/or futility at the interim analyses will be calculated based on the actual number of the TTCI events documented at the time the interim analysis.

The interim analyses will be performed by an independent statistician external to the study team. The results of the interim analyses will be presented to the IDMC by the independent statistician. The IDMC will provide the Sponsor with a recommendation to continue the trial as planned, to have an interruption, to make a protocol amendment, or to terminate the trial. If the interim analysis demonstrates significant shortening of TTCI in the SCTA01 group compared to the control group, IDMC could recommend to terminate and un-blind the study, and the study could be announced as completed.

At the time of this SAP version, no patients globally are enrolled in the study.

## 2.3 Sample Size and Power

The treatment estimates are based on the study of Remdesivir for COVID-19. The rate ratio (RR) for recovery for the severe patients, the patients with 5-point of baseline ordinal score and the patients with 6-point of baseline ordinal score is 1.31, 1.45 and 1.09 respectively (Beigel et al., 2020; website link: reference 18). The event rate for the patients with 5-points is 88.8% and 76.8% for Remdesivir group and placebo group, respectively. The assumption to calculate the sample size is set on the data from 5-point patients and we set RR as 1.4 and event rate as 80%. The sample size aims at getting a meaningful clinical difference.

Sample size is to be calculated based on the following assumptions:

- Overall Type-I error rate should not exceed 2.5% (1-sided);

- Rate ratio (RR) sets to be 1.4 (if the median TTCI is 9 days on the placebo group, the SCTA01 group will be 6.5 days under the assumption of exponential distribution of event times);
- Randomization ratio of study drug group and placebo group in Phase III is 1:1;
- Randomization ratio of medium dose group, high dose group and placebo group in Phase II is 1:1:1;
- 80% event rate;
- Sample size ratio of Phase II and Phase III is 1:1.79(sample size ratio of the same dose group advanced from phase II to phase III or placebo group in Phase II and Phase III is 1:2.68);
- Simes method will be applied to attain adjusted *p*-value from Phase II;
- Inverse normal weighting method will be used to combine *p*-value from Phase II and Phase III part;
- The 3rd interim analysis, which is the penultimate analysis for superior efficacy stopping and futility analysis, will be conducted when 31% of the TTCI events have been observed from the Phase III part;
- Pocock boundary (Lan and DeMets, 1983) will be used to control overall Type I error within 1-sided 2.5%;
- The critical values of nominal *p*-value will be re-calculated based on the actual number of TTCI events documented at the time of interim analysis and final analysis.

The study will randomize 795 patients totally, with Phase II of 285 patients and Phase III of 510 patients (255 in each group), providing over 90% power.

# 3. Efficacy and Safety Variables

## 3.1 Primary Efficacy Endpoint

- Time to clinical improvement (TTCI) up to Day 29. TTCI is defined as the time (in days) from randomization to the first day on which a patient satisfies point 1, 2, or 3 on the 8-point ordinal scale[1] and maintains a score ≤ 3 at least 48 hours (initial improvement) and maintains this up to Day 29 (sustained improvement) (Phase II, III).

## 3.2 Secondary Efficacy Endpoints

- Clinical efficacy of SCTA01:

- Clinical improvement:
  - Proportion of patients with initial clinical improvement at Day 29 (Phase II, III);
  - Proportion of patients with sustained clinical improvement at Day 29 (Phase II, III);
  - Time to initial clinical improvement up to Day 29 (Phase II, III);
- Hospitalization:
  - Time to discharge from hospital, followed by being alive and home for 14 consecutive days prior to Day 90;
  - Proportion of patients discharged from hospital, followed by being alive and home for 14 consecutive days prior to Day 90;
  - Time to hospital discharge up to Day 29 (Phase II, III);
  - Proportion of patients who are discharged from acute care up to Day 29 (Phase II, III);
- Mortality:
  - All-cause mortality up to Day 29, record date and cause of death (if applicable) (Phase II, III);
- Clinical severity：
  - Percentage of subjects in each category on the 8-point ordinal scale (time frame: Days 3, 5, 8, 11, 15, 22, and 29);
  - Proportion of patients alive and free of respiratory failure up to Day 29 (Phase II, III);
  - Days of supplemental oxygen (if applicable) up to Day 29 (Phase II, III);
  - Days of non-invasive ventilation/high flow oxygen (if applicable) up to Day 29 (Phase II, III);
  - Days of invasive mechanical ventilation/extracorporeal membrane oxygenation (ECMO) (if applicable) up to Day 29 (Phase II, III);
- Others：
  - Number and proportion of non-responders having mutations in the gene encoding the SARS-CoV-2 S protein through Day 29. A non-responder is defined as a patient who does not achieve a reduction in viral shedding

or persistence viral presence or have a rebound in viral shedding following suppression (Phase II, III).

- Virologic efficacy of SCTA01:
  - Change from baseline in viral shedding as measured by quantitative reverse transcription polymerase chain reaction (RT-qPCR) in NP swab samples (Phase II, III);
  - Time-weighted average change from baseline in viral shedding as measured by RT-qPCR in NP swab samples (Phase II, III);
  - Time to SARS-CoV-2 RNA negative in NP swab samples as determined by RT-qPCR (Phase II, III);
  - Proportion of patients with high viral titers (>10^4 copies/mL)
  - Proportion of patients with low titers below limit of detection
  - Proportion of patients with low titers below lower limit of quantitation
  - Viral load over time and virologic outcomes according to baseline viral load (>10^4,>10^5, >10^6, or >10^7 copies per milliliter).
- Immunogenicity Endpoints:
  - Immunogenicity assessment include the number and percentage of subjects who develop detectable ADA.
- Exploratory Endpoints
  - The correlation between the baseline serological antibody level and therapeutic effect (Phase II, III);
  - Quantitative SARS-CoV-2 virus in patient’s blood (Phase II, III).

## 3.3 Safety Variables

- Cumulative incidence of serious adverse events (SAEs) (Phase II, III);
- Cumulative incidence of Grade 1, 2, 3 and 4 clinical and/or laboratory adverse events (AEs) (Phase II, III);
- Discontinuation or temporary suspension of infusions (for any reason) (Phase II, III);
- Chemistries, hematology, urinalysis, coagulation function, IL-6, ferritin, procalcitonin, thrombin time, D-dimer (Phase II, III);
- Number and proportion of patients with antibody-dependent enhancement (ADE) (Phase II, III).

# 4. Pharmacokinetic/Pharmacodynamic variables

Pharmacokinetic characteristics in severe COVID-19 patients, including individual SCTA01 concentrations (Phase II).

# 5. Analysis populations

The following analysis sets will be defined for phase II and phase III part of the study separately and combined.

## 5.1 All Enrolled Subjects Set

All patients who sign the informed consent form (ICF) will be included in the All Enrolled Subjects Set.

## 5.2 Safety Set (SS)

All patients who are randomly assigned and receive a dose of study drug will be included in the SS. All safety analyses will be based on the SS, with patients analyzed according to the treatment they received.

## 5.3 Intent-to-treat (ITT) Set

The ITT set will consist of all patients who are randomly assigned and received a dose of study drug. This is the primary analysis set for the efficacy analyses, and patients will be analyzed according to the treatment to which they are randomly assigned.

## 5.4 Full Analysis Set (FAS)

All patients who are randomly assigned, receive a dose of study drug and have at least one primary endpoint measurement. This analysis set will support the robustness of the primary efficacy results and patients will be analyzed according to the treatment to which they are randomly assigned.

## 5.5 Per Protocol Set (PPS)

The PPS will consist of all patients in the ITT set who do not have any major protocol deviations which may affect efficacy evaluation.

Protocol deviations are defined as any change, divergence, or departure from the study design or procedures defined in the study protocol. Major protocol deviations are a subset of protocol deviations and may significantly impact the correctness, accuracy, and/or reliability of the study data or that may significantly affect a subject’s rights, safety, or well-being. Section 5.5.1 details the deviations.

### 5.5.1 Major Protocol Deviations Leading to Exclusion from the PPS Analysis

Only those major protocol deviations considered to have a major effect on efficacy will lead to complete exclusion of the patients from the PPS.

All major protocol deviations leading to exclusion from the PPS occurring during the study will be reviewed and approved by Sinocelltech, Ltd. prior to database lock and unblinding.

## 5.6 Pharmacokinetics set (PKS)

Patients receiving single-dose investigational drug and having at least one blood-drug concentration data point will be included in the PKS.

## 5.7 Seronegative ITT

The seronegative ITT are defined as all randomized patients with documented seronegative status at baseline in ITT. A patient's serostatus is considered to be negative if the anti-spike protein antibodies test is negative.

## 5.8 Seropositive ITT

The seropositive ITT are defined as all randomized patients with documented seropositive status at baseline in ITT. A patient's serostatus is considered to be positive if the anti-spike protein antibodies test is positive.

# 6. DATA Handling

## 6.1 Time points and Visit Windows

Day 1 is defined as the day of first dosing of study treatment. Relative days on or after Day 1 are calculated as (target date – Day 1 date) + 1. Relative days prior to Day 1 are calculated as (target date – Day 1 date). The day prior to Day 1 is Day-1.

For by-visit summaries, the non-missing assessment recorded at each nominal visit as defined in Appendix II will be summarized. When there are repeated assessments within the window of a visit, the value is determined based on the proximity to the nominal visit day. When there are repeated assessments with equal proximity to the nominal visit day, the one occurring earlier will be used. For example, if an assessment intended for Day 15 (±2) is carried out both on day 13 and day 17, the former will be used as result of Day 15. For across visit summaries (e.g. maximum post-baseline value), scheduled, unscheduled and repeated assessments will all be considered.

Unless otherwise specified, assessments carried out on day of first study treatment administration are considered to have taken place before the study treatment administration, if the corresponding times have not been recorded.

## 6.2 Baseline definition

Baseline value is defined as the last scheduled or unscheduled value collected prior to the first dose of study treatment.

## 6.3 Handling of Dropouts, Missing Data, and Outliers

8-point ordinal assessment will be planned on daily basic, below are the rules to handle missing value of 8-point ordinal assessment within Day 29.

- Missing on Day 29 will be treated as non-TTCI.
- Missing before the first score of =<3 will be treated as >3 when determining TTCI event.
- Missing within 48 hours after the first score of =<3 but with a score of =<3 after 48 hours will be treated as =<3.
- Missing after 48 hours from the first score of =<3 but with a score of =<3 on Day 29 will be treated as =<3.

Whenever applicable, subjects with missing yes/no response will be all treated as non-responders (NRI method) for secondary efficacy endpoints. For endpoints concerning sustained change of outcome/condition which may require an extended period of follow-up, when accumulating data suggests an imbalance is observed of number of drop-out and/or missing data between treatment group and placebo, due to the reason that Covid-19 is a new disease without known pattern of subject's behavior after treatment and/or disease cure, some other missing data treatment approach may be employed to summarize the data, which may be introduced when applicable.

Missing viral shedding values with less than the lower limit of quantification of the PCR assay are imputed with 0 log10 copies/ml (or 1 copy/ml). A result of "< 500 copies/ml" will be imputed with 499 copies/ml when applicable.

See section 7.8.2 for the handling of missing data for adverse events.

For time-to-event efficacy variables, the censoring rules will be included in efficacy analysis section.

For the derived age, when applicable, the following imputation method will be used for the birth date: June-30 will be imputed for a missing month and a missing day, and 15 will be imputed for a missing day.

The same rules to impute missing parts of age will be applied to other dates including medical history start date and previous medication date. In case that the imputed date is after randomization, the date will be set as one day before randomization.

Dates in original form will be used in all listings.

No rules for outlier detection are planned.

# 7. Statistical Methods

## 7.1 General Principles

All data processing, summarization and analyses will be performed with the SAS software Version 9.4 or later.

Analyses will be performed for Phase II data, Phase III data and combined data separately.

The following principles will be applied to all TFLs unless otherwise stated:

Table 2: General Principles for TFLs

| Principle | Value |
|---|---|
| Treatment group labels and order | SCTA01 15 mg/kg<br>SCTA01 50 mg/kg<br>Placebo |
| Tables | Data in summary tables presented by treatment group, assessment and visit (where applicable). |
| | |
| Listings | All data collected presented by treatment group, country, site, Patient, assessment and visit (where applicable), unless otherwise specified. |
| Descriptive summary statistics for continuous variables | Number of patients/observations (n), mean, standard deviation (SD), median, minimum (Min) and maximum (Max).<br>If applicable, Standard error, Least Square Mean and Confidence Interval will be included |
| Descriptive summary statistics for categorical variables | Frequency counts and percentages [n (%)] |
| Denominator for percentages | Number of patients in the analysis population, unless stated otherwise in table shell(s) |

| Principle | Value |
|---|---|
| Include "Missing" as category | No. |
| Display to one more decimal place than collected value | For efficacy and safety:<br>Mean<br>Mean Difference<br>Median |
| Display to two more decimal places than collected value | For efficacy and safety:<br>Standard Error<br>Standard Deviation<br>Confidence Interval |
| Limit of precision for displays | 4 decimal places |
| Date Format | YYYY-MM-DD |
| Date Time Format | YYYY-MM-DD HH:MM |

## 7.2 Subject Disposition and Data Sets Analyzed

Patient disposition will be listed and summarized by treatment group and overall and will include the number and percentage of patients:

- screened (overall);
- screen failed;
- eligible but not randomized;
- randomized;
- treated;
- included in each study population (all enrolled subjects set, ITT set, FAS, PPS, SS, PKS, seronegative ITT, seropositive ITT);
- completed/discontinued from the study, including a breakdown of the primary reasons for discontinuation.

## 7.3 Protocol Deviations

All protocol deviations will be listed for enrolled set and summarized by treatment group for the ITT set.

All major protocol deviations leading to exclusion from the PPS will be listed and summarized by treatment group for the ITT set.

The deviations will be identified before treatment assignments are unblinded.

## 7.4 Baseline Disease Characteristics

Baseline disease characteristics will be listed and summarized by treatment group and overall for the ITT set. If applicable, standard descriptive statistics will be presented for the variables of:

- Disease severity (clinical status via 8-point ordinal scale)
- Duration of symptoms prior to enrollment (Days)
- Categories of Duration of symptoms prior to enrollment (1-7days,8-10days)
- SpO2(%)
- Screening SARS-CoV-2 infection
- Baseline SARS-CoV-2 infection

## 7.5 Demographics and Other Baseline Characteristics

Demographic and other baseline characteristics will be listed and summarized by treatment group and overall for the ITT set. Standard descriptive statistics will be presented for the continuous variables of:

- age (years);
- weight (kg);
- height (cm);
- body mass index (BMI, $kg/m^2$) [calculated as ($weight/height^2$) where weight is in kg and height is in m].

The total counts and percentages of patients will be presented for the categorical variables of:

- country of enrollment
- age (18-44, 45-64, 65-84, >=85years)
- sex;
- race;
- ethnicity;
- BMI (<30, 30-34.9, 35+)

Some biomarkers will be measured centrally from stored samples, for example, anti-SARS-CoV-2 spike protein. If these measures are available, they will be included in interim reports.

Other baseline measurements, such as vital signs and ECG, will be summarized with the post-baseline measurements.

### 7.5.1 Medical History and Prior and Concomitant Medication

Medical history including but not limited to chronic oxygen requirement prior to onset of COVID-19 will be coded using Medical Dictionary for Regulatory Activities (MedDRA) [Version 23.1 dated 1 Sep 2020 or a later version if updated during the study (exclusively meant for COVID-19)]. All medical history will be listed, and the number and percentage of patients with any medical history will be summarized for the ITT set by system organ class (SOC) and preferred term (PT) for each treatment group and overall.

Medications received prior to or concomitantly with study treatment will be coded using WHODrug Dictionary [Version Global B3 March 2020 (or a later version if updated during the study)].

Prior medications and concomitant medications are defined as follows:

Prior medications are those with a stop date prior to the first dose date of study treatment.

Concomitant medications are those with a start date on or after the first dose date of study treatment, or those with a start date before the first dose date of study treatment and a stop date on or after the first dose date of study treatment or ongoing end of study.

If a medication cannot be classified as “prior” or “concomitant” due to missing/incomplete dates, it will be classified as concomitant.

The number and percentage of patients using each medication will be displayed together with the number and percentage of patients using at least one medication within each therapeutic class (ATC-Level 2), chemical subgroup (ATC-Level 4), and generic term.

#### 7.5.5.1 Prior and Concomitant Therapy and Procedures for COVID-19 (Best Supportive Care)

Prior and concomitant therapy and procedures for COVID-19 will be summarized by treatment group separately in a similar manner as described for the prior and concomitant medication.

## 7.6 Measurements of Treatment Compliance

Treatment compliance is measured by the proportion of patients who fully received planned dosage. Treatment compliance and frequency and percentage of patients who do not fully receive planned dosage will be summarized by treatment for the SS.

## 7.7 Efficacy

Generally, the primary analysis set is the ITT for all efficacy analyses. A combined *p*-value using weighted inverse normal method of less than 1-side of 0.01068 from 3rd interim analysis or less than 1-side of 0.0166 from final analysis will need to be observed to declare statistical significance. All patients from Phase II and Phase III will be utilized together to get the combined p-value. The total number of patients in the ITT analysis sets for primary analysis will include patients in the recommended dose group in phase II and in the same dose group that continued in phase III, or the control group. i.e., 95 + 255 = 350 patients for recommended dose group or for control group. The patients from the inferior dose group (95 patients) in phase II will be only contributed for adjusting p-value of Phase II. Wald tests will be used and its associated P value will be obtained when applicable. Treatment policy strategy will be applied for primary endpoint and secondary endpoints.

### 7.7.1 Statistical Hypothesis:

As generally applicable to efficacy analyses, the statistical hypothesis is the defined as the following: the null hypothesis is that there is no treatment group difference and the alternative hypothesis is that a treatment group difference exists favoring the treatment arm than the placebo arm.

When applicable, analyses will be performed based on the Phase II and Phase III proportion of the data, respectively, as well as for the Phase II and Phase III combined.

### 7.7.2 Primary Efficacy Analysis

The primary analyses will be based on the ITT population.

The primary endpoint is time to clinical improvement (TTCI) up to Day29. TTCI is defined as the time (in days) from randomization to the first day on which a patient satisfies point 1, 2, or 3 on the 8-point ordinal scale and maintains a score of ≤ 3 at least 48 hours (initial improvement) and maintains this up to Day 29(sustained improvement).

The estimands for the primary efficacy variable are described in relation the following intercurrent events.

Intercurrent Events (may occur between randomization and Day 29), not mutually exclusive
IE1: Participant withdrew consent for follow-up and assessment of endpoints prior to or on Day 29

IE2: Participant was lost to follow-up prior to Day 29

IE3: Participant received concomitant anti-viral or antibody-based treatments not permitted by protocol at study initiation, but which become standard of care during the conduct of the trial.

**Primary Estimand: Treatment Policy Strategy**

- Objective: To assess the efficacy of SCTA01+SOC compared to SOC in each country among participants not receiving drugs with the same mechanism of action as SCTA01.
- Participant population: as defined by ITT analysis set
- Treatment conditions:
    - Active: Randomized to selected dose of SCTA01 and treated with any medication
    - Control: Randomized to placebo and treated with any medication
- Endpoint: time to clinical improvement (TTCI) up to Day29.
- Population-level summary: p-value/RR for TTCI between the treatment and placebo group.

Main Estimator for Estimand1: Logrank test for TTCI up to Day 29, stratified by country, duration of symptoms (1-7 days, 8-10 days), comorbidities number (0,1,2,>2), baseline anti-S protein antibody (positive, negative) as covariates.

All Intercurrent events will be ignored as Treatment Policy Strategy will be applied.

The following examples meet the criteria for TTCI：

Case 1： the first score of =< 3-point happens on Day X(any day between D2-D27) and maintains a score of =<3 up to Day29

Case 2：the first score of =< 3-point happens on Day 27 maintains a score of =<3 up to Day 29

Case 3：the first score of =< 3-point happens on Day 7 maintains it up to D15. The score rises to 4 on Day 16, but falling to a score of =<3 on Day X(before D27) and maintains it up to Day 29.

The following examples do not meet the criteria for TTCI:

Case 1：the minimum score is >3 up to Day 29.

Case 2：the first score of =< 3-point happens on Day 28 or after Day 28

Case 3：the first score of =< 3-point happens on Day 7 maintains it up to D15. The score rises to 4 on Day 16 and maintains it up to Day 29. It only meets the criterion for initial improvement.

Table 3: Censoring Scheme for TTCI (Primary Analysis)

| | Situation | Date of Event or Censoring | Outcome |
|---|---|---|---|
| 1 | No post-baseline assessment(Excluding death or termination) | Date of randomization | Censored |
| 2 | Death within 29 days | Day 29 | Censored |
| 3 | Terminated within Day 29 due to AE | Day 29 | Censored |
| 4 | Terminated within Day 29 due to other reason | the day of their last observed assessment | Censored |
| 5 | Take prohibited medications within Day 29 | the day of their last observed assessment | Censored |

8-point ordinal assessment will be planned on daily basic, below are the rules to handle missing value of 8-point ordinal assessment within Day 29.

- Missing on Day 29 will be treated as non-TTCI.
- Missing before the first score of =<3 will be treated as >3 when determining TTCI event.
- Missing within 48 hours after the first score of =<3 but with a score of =<3 after 48 hours will be treated as =<3.
- Missing after 48 hours from the first score of =<3 but with a score of =<3 on Day 29 will be treated as =<3.

A stratified log-rank test with country as strata, and duration of symptoms (1-7 days, 8-10 days), comorbidities number(0，1，2，>2) ，baseline sero-status (positive，negative) as covariates will be performed.

Besides those mentioned above, other stratification factors for the analysis may be declared at the time of randomization.

Kaplan-Meier estimates of the treatment and placebo group will be calculated and plotted by treatment group. A Cox model will be used to calculate the rate ratio and its two-sided 95% confidence interval between the treatment and placebo group. The strata will be the same as described for the log-rank test. This stratified Cox model includes treatment effect, duration of symptoms (1-7 days, 8-10 days), comorbidities number(0, 1, 2, >2), baseline sero-status(positive, negative) as covariates. Efron method (Efron, 1977) will be used to handle the ties of event time. It is assumed that the rate of the treatment group is proportional to that of the placebo group within a stratum. Independence between the censoring time and the event time is also assumed.

Further descriptive results include the total number of subjects, the number and percentage of subjects with a sustained TTCI event, number and percentage of subjects censored. Median sustained TTCI event time in days and its two-sided 95% will be calculated. All descriptive results will be shown by treatment group.

The overall test statistic will be produced using the weighted inverse normal method (Lehmacher and Wassmer, 1999; Bauer and Koehne 1994) as follows:

$$\mathrm{Sqrt}(n_1/n) * \Phi^{-1}(1-p_1) + \mathrm{Sqrt}(n_2/n) * \Phi^{-1}(1-p_2)$$

Where Sqrt stands for the square root, $n_1$= 285 and $n_2$= 510 are the pre-planned sample size in Phase II and III, respectively, $n = n_1+n_2$= 795, Φ refers to the standard normal distribution, and $p_1$ and $p_2$ are the p-value from Phase II and III, respectively. $P_1$ will be calculated using the closure principle and Simes method (see section 7.13 for details).

Final analysis will be performed when 556 TTCI events have been observed. A combined p-value based on the inverse normal weighting method of less than 1-side of 0.0166 will need to be observed to declare statistical significance. The above derived boundaries are based on the assumption that the final analysis is performed when exactly 556 events are observed from both Phase II and Phase III part. If this assumption does not hold, the boundaries for p-value will be re-calculated according to the Pocock-type α-spending function.

### 7.7.3 Secondary Efficacy Analysis

#### 7.7.3.1 Virologic efficacy Analysis

All virologic efficacy analyses will be performed on the following analysis sets: ITT, PPS, seropositive ITT and seronegative ITT (see section 5), respectively. The primary analysis set is the ITT.

In addition to the comparison of high- and low-dose group to the placebo group described below, comparison of treatment group comprising any patient in high- or

low-dose group and placebo group will be made as well. This will be done only for the Phase II virologic efficacy analysis.

#### 7.7.3.1.1 Change from baseline in viral shedding as measured by RT-qPCR in NP swab samples (Phase II, III)

For this endpoint, scheduled post-baseline visits are the following: Day 3,5,8,11,15,29,120, or early withdraw.

All analyses will be based on logarithm transformation (base 10) of viral load. A linear mixed model will be used to model the viral load change from baseline with the following fixed effects: country, baseline viral load, treatment group, visit, and treatment group by visit interaction. As mentioned earlier, the following treatment group assignments will be considered in two separate models at the end of phase II analysis: 15mg/kg vs 50 mg/kg vs placebo, and combined active treatment group (defined in section 7.7.3.1) vs placebo. Unstructured with-in subject covariance structure will be assumed. If a convergence issue arises, a more parsimonious with-in subject covariance structure will be considered. The treatment differences (high dose versus placebo, low dose versus placebo) in least squares means will be presented as well as its two-sided 95% confidence interval. P-values will be provided for pair-wise comparison. Multiplicity adjustment is described further in section 7.7.7.

As specified in the protocol, if the NP swab is not applicable, the OP swab result will be treated interchangeably with the NP swab. Maximum likelihood approach will be used regarding the missing data. Analyses will be based on the observed data.

The viral load and its change from baseline will also be summarized descriptively by treatment for each visit.

A corresponding plot will be made, plotting the mean and the standard error of the viral load on the Y-axis and the day on the X-axis by treatment group.

The same plot will be repeated for patients with a baseline viral load of $>10^4$, $>10^5$, $>10^6$, and $>10^7$copies per milliliter, respectively, if applicable.

#### 7.7.3.1.2Time-weighted average change from baseline between SCTA01 group and placebo group in viral shedding as measured by RT-qPCR in NP swab samples (Phase II, III)

The following analyses will be repeated for each of the following scheduled visits: 3,5,8,11,15,29,120, and for the early withdraw.

Time-weighted average of change from baseline viral load in the nasopharyngeal (NP) swab samples from day 1 through day *n* will be calculated for each patient using the linear trapezoidal rule as the area under the curve for change from baseline at each time point divided by the time interval for the observation period.

See Appendix 3 for a detailed description of the calculation of the time-weighted average change from baseline of viral load.

For each treatment dosage and specific for a visit, the null hypothesis is that the time-weighted viral shedding (or viral load) change from baseline are the same for the treatment and the placebo group. An analysis of covariance model (ANCOVA) will be used. The response variable is the time-weighted viral load change from baseline, and the fixed effects include the treatment, country and baseline viral load. The treatment differences (high dose versus placebo, low dose versus placebo) in least squares means will be presented as well as its two-sided 95% confidence interval. It is assumed that for the ANCOVA analysis, the errors are independent and normally distributed with zero mean and constant variance. Also it is assumed that the regression slope does not change with group. Missing values will not be imputed.

Similar to the descriptive analysis defined in section 7.7.2.1.1, the time-weighted viral load change from baseline will be summarized descriptively by treatment for each visit on which a NP swab is performed. A corresponding plot will be made, plotting the mean and the standard error of the time-weighted viral load change from baseline on the Y-axis and the day on the X-axis by treatment group.

The same plot will be repeated for patients with a baseline viral load of $>10^4$, $>10^5$, $>10^6$, and $>10^7$copies per milliliter, respectively, if applicable.

#### 7.7.3.1.3 Time to SARS-CoV-2 RNA negative in NP swab samples as determined by RT-qPCR (Phase II, III)

This is defined as time in days from randomization to the day on which SARS-CoV-2 RNA in NP swab samples turns negative up to the last visit. When there are more than once that the SARS-CoV-2 RNA in NP swab samples turns negative up to the last visit, the time is defined as the earliest one.

This will be summarized and analysed as a time-to-event endpoint. Kaplan-Meier estimates will be calculated and plotted by treatment group. A log-rank test stratified by country will be performed for the treatment effect on the rate. The rate ratio will be calculated with a Cox model stratified by country, including treatment as the covariate. 95% confidence intervals of the rate ratio will also be given.

Further descriptive results include the total number of subjects, the number and percentage of subjects with the event, number and percentage of subjects censored. Median event time in days and its two-sided 95% will also be calculated. All descriptive results will be shown by treatment group.

The censoring will be determined based on the following table:

Table 4: Censoring scheme for Time to SARS-CoV-2 RNA negative in NP swab samples as determined by RT-qPCR (Phase II, III)

| | Situation | Date of Event or Censoring | Outcome |
|---|---|---|---|
| 1 | No post-baseline assessment(excluding death or termination) | Date of randomization | Censored |
| 2 | death within 120 days | the day of their last observed assessment before Day 120 | Censored |
| 3 | terminated within Day 120 due to AE | the day of their last observed assessment before Day 120 | Censored |
| 4 | terminated within Day 120 due to other reason | the day of their last observed assessment before Day 120 | Censored |
| 5 | take prohibited medications within Day 120 | the day of their last observed assessment before Day 120 | Censored |

#### 7.7.3.1.4 Proportion of patients with high viral titers (>$10^4$ copies/mL)

The analysis will be repeated for each of the scheduled post-baseline visits are the following: Day 3,5,8,11,15,29,120, and for the earlier withdraw if applicable.

A logistic regression model will be used, where the response is the logit of the binary response (viral titers>$10^4$or ≤$10^4$) and the factors are: treatment, country, duration of symptoms (1-7 days, 8-10 days), comorbidities number (0,1,2,>2) and baseline sero-status. The odds ratio (OR) and its two-sided 95% confidence interval for the treatment effect will be given. Descriptively, the frequency and percentage of patients in each category will be shown by treatment. The descriptive results will be shown overall and for each country.

#### 7.7.3.1.5 Proportion of patients with low viral titers below limit of detection (copies per ml)

This will be analysed in the same way as specified in the last section (7.7.2.1.4).

#### 7.7.3.1.6 Proportion of patients with low viral titers below lower limit of quantitation (500 copies per ml)

This will be analysed in the same way as specified in the last section (7.7.2.1.4)

#### 7.7.3.2 Clinical Efficacy Analysis

##### 7.7.3.2.1 Analyses of the days of supplemental oxygen (if applicable) up to Day 29 (Phase II, III), and other continuous secondary efficacy endpoints

Days of supplemental oxygen (if applicable) up to Day 29 (Phase II, III) will be summarized descriptively. The number and percentage of patients with zero and one or more days will be presented by treatment. The number of days will also be summarized using mean, median, standard deviation, lower and upper quartile, minimum and maximum. Wilcoxon rank sum test will be used for the comparison of days between treatments. Pairwise comparisons of high-dose with placebo, and low-dose with placebo will be conducted.

The following secondary clinical efficacy variables will be analysed and summarized similarly as described in the preceding paragraph: Days of non-invasive ventilation/high flow oxygen (if applicable) up to Day 29 (Phase II, III), Days of invasive mechanical ventilation/extracorporeal membrane oxygenation (ECMO) (if applicable) up to Day 29 (Phase II, III).

##### 7.7.3.2.2 Analyses of proportion of patients with initial clinical improvement up to Day 29 (Phase II, III), and other binary secondary efficacy endpoints

These secondary efficacy variables are binary variables:

- Proportion of patients with initial clinical improvement up to Day 29 (Phase II, III),
- Proportion of patients with sustained clinical improvement at Day 29 (Phase II, III),
- Proportion of patients discharged from hospital, followed by being alive and home for 14 consecutive days prior to Day 90 (Phase II, III);
- All-cause mortality up to Day 29, record date and cause of death (if applicable) (Phase II, III),
- Proportion of patients alive and free of respiratory failure up to Day 29 (Phase II, III), Free of respiratory failure up to day 29 is defined that there is no respiratory failure during the first 29 days after randomization.
- Proportion of patients who are discharged from acute care up to Day 29 (Phase II, III).

A separated SAP for mutation endpoints may be developed as needed.

For binary variables, a logistic regression model will be used, where the response is the logit of the binary response and the factors are the same as primary efficacy analysis. The odds ratio (OR) and its two-sided 95% confidence interval for the treatment effect will be given. Descriptively, the frequency and percentage of patients in each category will be shown by treatment. The descriptive results will be shown overall and for each country.

#### 7.7.3.2.3 Analyses of percentage of subjects in each category on the 8-point ordinal scale

The percentage of subjects in each category on the 8-point ordinal scale will be analysed for the following days: Day 3,5,8,11,15,22,29. A proportional odds model (ordinal logistic regression) will be used to analyse the ordinal responses. Proportional odd associated with a treatment will correspond to the improvement of clinical outcome. For example, for a particular post-baseline visit, if patients are categorized as either 2, 5 or 8 on the 8-point scale. This proportional odd will model the following cumulative logits: $\log(\frac{\pi_2}{\pi_5+\pi_8})$ and $\log(\frac{\pi_2+\pi_5}{\pi_8})$, where $\pi_i$ stands for the proportion of response for the scale *i* associated with a treatment. The following categorical covariates will be included in the model: treatment, country, duration of symptoms (1-7 days, 8-10 days), comorbidities number (0, 1, 2, >2) , baseline sero-status(positive, negative)).

If the proportional odds assumption is not satisfied, a partial proportional logit model will be considered. Unless otherwise specified, a p value of proportionality of 0.2 or less will be considered for selecting non-proportional covariates.

For each visit, the following descriptive statistics will be calculated for the ordinal scores treated as a continuous variable by group: mean, median, minimum, maximum, standard deviation. Means and standard error will also be plotted for each visit by treatment.

#### 7.7.3.2.4 Analyses of time to initial clinical improvement up to Day 29 (Phase II, III)

The time to initial clinical improvement is defined as the following: time (in days) from randomization to the first day on which a patient satisfies point 1, 2, or 3 on the 8-point ordinal scale and maintains a score of ≤ 3 at least 48 hours up to Day 29. This secondary endpoint will be analysed in the same way as described for the primary efficacy endpoint. Also, the censoring rules described for the primary efficacy endpoint applies, although that the events are different.

#### 7.7.3.2.5 Time to discharge from hospital, followed by being alive and home for 14 consecutive days prior to Day 90 (Phase II, III);

The event time is defined as the days from randomization to the day of hospital discharge, followed by being alive and home for 14 consecutive days prior to Day 90.

Table 5: Censoring Scheme

| | Situation | Date of Event or Censoring | Outcome |
|---|---|---|---|
| 1 | No post-baseline assessment for | Date of randomization | Censored |
| 2 | death within 90 days | Date of death | Censored |
| 3 | terminated within Day 90 due to AE | Date of termination | Censored |
| 4 | terminated within Day 90 due to other reason | Date of termination | Censored |

#### 7.7.3.2.6 Analyses of Time to hospital discharge up to Day 29 (Phase II, III).

Time to first hospital discharge is defined as the days from randomization to the day of hospital discharge up to Day 29. This secondary endpoint will be analysed in the same way as described for the primary efficacy endpoint. Also, the censoring rules described for the primary efficacy endpoint applies, although that the events are different.

### 7.7.4 Sensitivity Analysis

For the primary efficacy analysis(TTCI), the following analyses will be performed to probe the sensitivity of results by changing different analysis set or censor rules:

- Using FAS
- Using PPS
- All patients who died or are lost to follow-up due to AE are censored to the day of death or the day of lost to follow up;
- All patients on SCTA01 who are lost to follow-up due to other reason are censored on the day of their last observed assessment; and all patients on placebo are assumed to have completely recovered (i.e. an event is observed on last day known alive for such patients).

- An unstratified log-rank test/ Cox regression will be performed.
- A stratified Gehan's Wilcoxon test will be conducted in a similar manner to the stratified log-rank test for primary efficacy for the ITT set.
- Using while-on-treatment policy, the patients who take prohibited medication will be censored at the day of taking prohibited medication.

Some other sensitivity analyses may be performed to investigate the robustness of the primary analysis.

### 7.7.5 Subgroup Analysis

Following subgroup factors will be studied for the primary efficacy endpoint (TTCI):

- Country
- Geographic region (USA; Mexico and South American Countries)
- Race (White; Black; Asian; Other)
- Ethnic group (HISPANIC OR LATINO; NOT HISPANIC OR LATINO; unknown)
- Duration of symptoms prior to enrolment (1-7 days; 8-10 days)
- Age (18 to <40 yr;40 to <65 yr; ≥65 yr.)
- Sex
- Comorbidities [Diabetes; Obesity (BMI≥35); Hypertension]
- Comorbidities number (1; >=1)
- Baseline anti-S protein antibody (Positive & negative)

Forest plots of the rate ratio and its two-sided 95% confidence intervals will be made by subgroup factor levels. The rate ratio is defined as the rate of the treatment group over that of the placebo group for the primary efficacy endpoint. The rate ratio will be calculated using Cox model. For each subgroup analysis listed above, a Cox model will be fitted including treatment group and that subgroup factor, and the interaction between the treatment and subgroup factor as covariates. P value of the interaction effect will be shown.

### 7.7.6 Exploratory Analysis

#### 7.7.6.1 The correlation between the baseline serological antibody level and therapeutic effect (Phase II, III)

For the primary efficacy endpoint of TTCI, a stratified Cox model will be used including the treatment group, baseline serological antibody level and the interaction between the treatment group and baseline serological antibody level as covariates. The

stratification is based on country. Rate ratio between treatment and placebo group will be shown with its two-sided 95% confidence interval. P value of the interaction effect will be shown.

An analysis similar to described above for the TTCI will also be performed with initial TTCI.

The following analyses will be done for those secondary efficacy variables: Proportion of patients with initial clinical improvement at Day 29 (Phase II, III); Proportion of patients with sustained clinical improvement at Day 29 (Phase II, III). A logistic regression model will be used, where the response is the logit of the binary response (1: clinical improvement, 0: no clinical improvement) and the covariates are: treatment, country, baseline serological antibody level and the interaction between the treatment and baseline serological antibody level. The OR and its two-sided 95% confidence interval for the treatment effect will be given. P value of the interaction effect will be shown.

#### 7.7.6.2 Quantitative SARS-CoV-2 Virus in Patient's Blood (Phase II, III)

This endpoint will be assessed for the following scheduled visits: Day 3,8,15,29,120. The analysis of the viral load in blood will be similar to that of the viral load change from baseline in NP swaps described in section 7.7.2.1.1.

Quantitative SARS-CoV-2 virus results will be summarized descriptively by mean, median, maximum, minimum, standard deviation and number of evaluable subjects by treatment and by visit. Change from baseline result will be summarized similarly.

A line plot will be made to show the mean of the viral load in blood for all post-baseline visits by treatment. Standard error will be used as error-bars.

### 7.7.7 Multiplicity Adjustment

Multiplicity to account for selection bias at the end of phase II can be handled by adjusting p-values using Simes procedure (Simes, 1986) and closure principle. Simes-adjusted p value for a global null (intersection) hypothesis $H_1 \cap H_2 \cap \ldots \cap H_m$ is $p_{sim}$= m x min( $p_{(1)}$, $p_{(2)}/2$, …, $p_{(m)}/m$), where $H_1$, $H_2 ... H_m$ represents first, second and m-th individual hypothesis, $p_1$, $p_2$,…,$p_m$ represents the p values of corresponding null hypothesis, and $p_{(1)}$,$p_{(2)}$,…,$p_{(m)}$ represents the ordered p values, form smallest and largest, min is the operation of taking the minimum. m will equal 2 in the current application. The adjusted p-values at the end of phase II will be calculated using Hommel procedure as the following: $p_{(1)\_adj}$=max($p_{(1)}$, 2 x min($p_{(1)}$, $p_{(2)}/2$)) and $p_{(2)\_adj}$=max($p_{(2)}$, 2 x min($p_{(1)}$, $p_{(2)}/2$)), where max means taking the maximum. Adjusted p-values will be 1 if the calculated value exceeds 1.

The general principle of "adaptive combination test" will be adhered to. Independence of the test statistics from Phase II and Phase III is achieved by fixing the follow-up time to obtain events required for the efficacy analysis for each Phase, thereby ensuring control of the type I error via the closure principle.

The critical values of the Pocock boundary is calculated using the sample sizes in the Phase III part alone. This then provides a conservative boundary to control the family-wise type I error rate better than 2.5% when these boundaries are compared to the p-values derived from combining the p-values from the Phase II and Phase III parts. The boundaries are conservative when the same weights are used for both the interim and final p-values because when the same weights are used, the correlation between the p-values at interim and final is stronger when the p-values are first combined with the same data from phase II than when they are not combined with the phase II data. Therefore, the adjustment of the final alpha required to control the type I error overall would be smaller and the process is guaranteed to protect against type I error inflation. This conclusion is supported by simulation results demonstrating that under the null hypothesis, the Type I error rate is less than 2.5%.

## 7.8 Safety

### 7.8.1 Extent of Exposure

The following variables will be summarized descriptively by treatment for the SS:

- Frequency and percentage of patients receiving complete infusion , and those of patients not receiving complete infusion.
- For the patients receiving complete infusion, the duration of the infusion (mean, median, minimum, maximum, standard deviation)
- Total dosage (mg) and dosage (mg/kg) for the patients receiving complete infusion (mean, median, minimum, maximum, standard deviation)
- Frequency and percentage of patients with treatment – emergent AEs (TEAEs) on Day 0 during or after infusion. Types of TEAEs will be summarized by system organ class and by severity grade.
- Number and reasons (AE, other) of infusion interruptions

### 7.8.2 Adverse Events

All adverse events (AEs) recorded on the eCRF will be coded using the MedDRA dictionary [Version 23.1 dated 1 Sep 2020 or a later version if updated during the study (exclusively meant for COVID-19)] and classified as either pre-treatment AEs or treatment – emergent AEs (TEAEs) as follows:

- Pre-treatment AEs are events that start after the signing the informed consent form and prior to the date of first dose of study treatment.
- TEAEs are events with start date on or after the date of first dose of study treatment and up to 150 days after date of last dose of study treatment or events with start date prior to the date of first dose of study treatment whose severity worsens on or after the date of first dose of study treatment.

If an AE can't be classified as pre-treatment AE or TEAE due to missing/partial dates, it will be classified as TEAE.

The number and percentage of patients reporting each pre-treatment AE will be summarized for each treatment group and overall, by System Organ Class (SOC) and Preferred Term (PT) for SS.

All AE data will be listed by treatment group and overall. Treatment-emergence status will be flagged in the listing. In addition, corresponding listings of serious AEs (SAEs), treatment-related AEs, AEs leading to discontinuation of study treatment, AEs leading to discontinuation of study and AEs resulting in death will be produced for the SS.

The severity of all AEs is recorded as Grade 1 to Grade 5. If severity is missing for a TEAE, it will be considered Grade 3 only in the overall category in the summary tables.

The relationship between an AE and treatment is assessed as unrelated, related. A treatment-related AE is an AE considered by the investigator as related or with missing relationship to treatment.

An overview table will summarize the number and percentage of patients with at least one of the following TEAEs, along with the number of corresponding TEAEs, where patients with more than one TEAE in a particular category are counted only once in that category:

- any TEAE;
- any TEAE by maximum severity;
- Grade 3 or above TEAE;
- TEAE leading to study treatment discontinuation;
- TEAE leading to study discontinuation AESI (acute allergic reactions)
- TE-SAE;
- Grade 3 or above TE-SAE;
- SAE leading to death;
- SAE leading to treatment discontinuation;

- SAE leading to study discontinuation.
- >=5%

The number and percentage of patients reporting each TEAE will be summarized by System Organ Class (SOC) and Preferred Term (PT) for the SS. Tables will be sorted alphabetically by SOC. PTs will be sorted by descending overall total. The following summaries will be produced:

- TEAEs, by SOC and PT;
- TEAEs related to study treatment, by SOC and PT;
- TEAEs by maximum severity, by SOC and PT;
- TEAEs related to study treatment by maximum severity, by SOC and PT;
- TEAEs causing discontinuation from study treatment, by SOC and PT;
- TEAEs related to study treatment causing discontinuation from study treatment, by SOC and PT;
- TEAEs causing discontinuation from study, by SOC and PT;
- TE-SAEs, by SOC and PT;
- TE-SAEs related to study treatment, by SOC and PT;
- TE-AEs leading to death, by SOC and PT.
- >=5% TEAEs, by SOC and PT
- >=5% TEAEs related to study treatment, by SOC and PT

In the above summaries, patients with more than one AE within a particular PT are counted only once for that PT. For summaries by maximum severity, patients with multiple AEs within a particular SOC or PT will be counted under the category of their most severe AE within that SOC or PT. AEs with missing severity will be included as severe in the overall count of patients with AEs, but will not be included in the counts of patients with AEs within a SOC or PT.

No statistical comparisons of AEs between treatment groups will be performed.

Cumulative incidence of AE is defined as the incidence of TEAE by time, and is calculated as total number of patients with TEAE divided by number of patients at risk for a particular time in days since dosing day (Day 0). The following cumulative incidence of AE will be plotted by treatment:

- Cumulative incidence of SAE (Phase II, III)
- Cumulative incidence of Grade 1 AE (Phase II, III)

- Cumulative incidence of Grade 2 AE (Phase II, III)
- Cumulative incidence of Grade 3 AE (Phase II, III)
- Cumulative incidence of Grade 4 AE (Phase II, III)

### 7.8.3 Laboratory Evaluations

Data for the following hematology, clinical chemistry, coagulation test, and urinalysis analytes will be listed and summarized by treatment group and overall by visit for the SS. If data for any additional analytes are also received/ recorded then these will be listed only.

Table 6: Listing of Laboratory Tests

| **Hematology** | **Clinical Chemistry** | **Coagulation test** | **Urinalysis** | **Other test** |
|---|---|---|---|---|
| Hemoglobin<br>Platelet count<br>Red blood cell (RBC) count<br>White blood cell (WBC) count<br>WBC differential<br>(% and absolute values):<br>• Basophils<br>• Eosinophils<br>• Lymphocytes<br>• Monocytes<br>• Neutrophils | Albumin<br>ALP<br>ALT<br>AST<br>Urea<br>Chlorine<br>Creatinine<br>GGT<br>Glucose<br>LDH<br>Potassium<br>Sodium<br>Total Bilirubin<br>Direct bilirubin Indirect bilirubin<br>Total Protein<br>Creatine kinase<br>C-reactive protein<br>Blood creatine | PT<br>APTT<br>Fibrinogen | pH<br>Specific gravity<br>Bilirubin<br>Glucose<br>Ketones<br>Nitrite<br>Occult blood<br>Protein<br>Urobilinogen<br>White blood cells<br>White blood cells (high power field)<br>Red blood cells<br>Red blood cells (high power field) | IL-6<br>Ferritin<br>Procalcitonin<br>TT<br>D-dimer |

Abbreviations: ALP = alkaline phosphatase; ALT = alanine aminotransferase; AST = aspartate aminotransferase; APTT =Activated partial thromboplastin time; CBC = complete blood count; GGT = gamma glutamyl transferase; LDH = lactate dehydrogenase; RBC = red blood cell; WBC = white blood cell; PT = Prothrombin time; TT = Thrombin time.

All laboratory data will be reported in International System of Units (SI), if applicable. Out-of-reference-range values will be flagged as high (H) or low (L) in the listings. Clinical significance will also be shown in the listings.

For analysis purposes, values preceded by a “<” or a “>”sign (i.e. those below or above the limits of quantification) will be multiplied by 0.5 and 1.5 respectively.

Results and changes from baseline (if applicable) of all laboratory parameters will be listed and summarized by treatment group and overall, for each scheduled visit using standard descriptive statistics for the SS.

Shift tables will be made showing the baseline outcome, and the worst outcome based on clinical significance ("Normal", "Abnormal, not clinically significant", "Abnormal, clinically significant") among all post-baseline visits.

For hematology, clinical chemistry and numerical urinalysis analyses, shift tables presenting movement in and out of reference range from baseline to each scheduled post-baseline visit will be provided for each treatment group and overall for the SS.

A summary (frequency and percentage) of any treatment emergent hepatobiliary laboratory abnormalities according to different pre-specified thresholds will be made by treatment group:

- ALT or/and AST >3 x ULN and ≤5 x ULN
- ALT or/and AST>5 x ULN
- Total bilirubin>2x ULN
- (ALT and/or AST >3 x ULN) and Bilirubin > 2 x ULN
- Hy's law lab. criteria:(ALT and/or AST >3 x ULN) and Bilirubin > 2 x ULN and ALP<2 x ULN

ULN = Upper limit of normal range.

### 7.8.4 Vital Signs and $SpO_2$

The following parameters will be listed and summarized by treatment group and overall and visit.

- systolic and diastolic blood pressure (mmHg);
- pulse rate (bpm);
- respiration rate (breaths/min);
- body temperature (°C);
- $SpO_2$ (%).

Vital signs data and changes from baseline in vital signs will be listed and summarized by treatment group and overall, for each scheduled visit using standard descriptive statistics for the SS.

$SpO_2$will be measured daily until discharge. For the $SpO_2$, a linear mixed model will be used including $SpO_2$as the response, treatment, country will be included as

categorical fixed effects, baseline $SpO_2$, days, squared days will be included as continuous fixed covariates. Interaction between the days and treatment, and interaction between squared days and treatment will also be included. Random effects will include intercept and slope. If a convergence issue arises, the random slope will be dropped leaving only a random intercept. P values will be shown for days and treatment interaction, as well as squared days and treatment interaction.

Transformation of $SpO_2$, for example a logarithm transformation, may be considered if deemed necessary for the analyses described above.

Descriptively, the mean and standard error of the $SpO_2$ will be plotted by days for each treatment group.

### 7.8.5 Electrocardiograms

The following quantitative ECG measurements will be taken during the study:

- heart rate (bpm);
- PR interval (msec);
- QRS interval (msec);
- QT interval (msec);
- Bazett corrected QT (QTcB) interval (msec);
- Fridericia corrected QT (QTcF) interval (msec).

An overall Investigator assessment of ECG will be provided (categories "normal", "abnormal, not clinically significant" and "abnormal, clinically significant").

The ECG measurements and changes from baseline in ECG will be listed and summarized by treatment group and overall for each scheduled visit using standard descriptive statistics for the SS.

The Investigator assessment will be listed and the number and percentage of patients within each assessment category will be tabulated by treatment group and overall for each scheduled visit for the SS.

Shifts from baseline in overall investigator assessment to the worst outcome across all post-baseline visits will be presented for each parameter available.

QTcB and QTcF intervals and change from baseline will be analyzed. The analysis will include all scheduled and unscheduled values.

**Clinical notable abnormalities thresholds of QTcB &QTcF**

The baseline and maximum post-baseline values will be summarized separately, by treatment group and overall according to the following categories for the QTcB:

- ≤450 ms
- >450 and ≤480 ms
- >480 and ≤500 ms
- >500 ms

The maximum increases from baseline will be summarized by treatment group and overall according to the following categories for the QTcF:

- ≤30 ms
- >30 and ≤60 ms
- >60 ms

### 7.8.6 Physical Examination

Physical examination results (categories “normal”, “abnormal, not clinically significant” and “abnormal, clinically significant”) and details of abnormalities will be listed for each patient.

For each physical examination body system, shifts from baseline in examination results to the worst outcome (based on the clinical significance) among all post-baseline visits will be presented by treatment group and overall for the SS.

For each physical examination body system, the number and percentage of patients with abnormalities at baseline and post-baseline will be summarized by treatment group for the SS.

### 7.8.8 Other Safety Variables

Other safety variables and corresponding data, if collected, will be listed only.

## 7.9 Pharmacokinetics Analysis

Blood samples will be analyzed for concentrations of SCTA01 for phase II part of the study. For the intensive sampling, PK blood sample collection will be performed within 0.5 h before the beginning of infusion, 1 h post end of infusion, D8, D15, D29 post end of infusion or at early withdraw. For the sparse sampling, PK blood sample collection will be performed on D8, D29 post end of infusion or at early withdraw. The Descriptive statistics (mean, median, standard deviation, min, max, inter-quartile range, and N) of serum concentrations will be created by treatment group. A by-participant, by-visit listing of PK concentrations will be created. A mean concentration-time profile by treatment group (15 mg/kg, 50 mg/kg) figure will be generated.

## 7.10 Immunogenicity Analysis

The immunogenic potential of SCTA01 will be assessed by summarizing the number and percentage of subjects who develop detectable ADA. The impact of ADA on PK will be assessed if data allow.

## 7.11 Interim Analysis

There will be three un-blinded interim analyses when 143rd patient in Phase II has completed Day 8 visit, at the end of Phase II and when the 160th patient in Phase III has completed Day 29 or Early Withdraw visit.

- The 1st interim analysis is for safety evaluation. The safety data will be reviewed at the 1st interim analysis, but the preliminary review of efficacy data is supported if early data and external emerging data warrants.
- The 2nd interim analysis is for dose selection: the analysis will be performed at the end of Phase II when the 285th patient completed Day 29 or Early Withdraw visit. The dose selected to continue to the Phase III part will be the dose with the greater effect in reduction of viral load compared to placebo at Day 8. Time to clinical improvement of each dose will be evaluated and the RR threshold of 1.15 will be used as a futility boundary at the end of Phase II. The futility boundary is non-binding, and the IDMC may recommend study continuation even if the futility bound is met on TTCI. If any safety signal is observed for any dose, the IDMC will weigh this information against the efficacy results in selecting the dose for Phase III.
- The 3rd interim analysis is the penultimate analysis that allows for stopping for superior efficacy. It is planned when 160 patients in Phase III (approximately 31% information time of Phase III part, which will have approximately 128 TTCI events) have been documented in the ITT population. If the interim analysis is performed at 31% of information time of Phase III, based on Pocock type boundary of α-spending function, a 1-sided *p*-value less than 0.01068 using inverse normal method to combine *p*-value from Phase II part and Phase III part will need to be observed to declare statistical significance. A futility boundary of an observed RR of 1.22 will be used to declare failure of the study when TTCI events from Phase II and Phase III part combined are exactly 280, which is equivalent to an observed one-sided p-value of 0.05.

# 8. Changes from Protocol

The following changes are made:

- Seronegative ITT and Seropositive ITT.

- More virologic efficacy variables are added including: proportion of patients with high viral titers (>10^4 copies/mL), proportion of patients with low titers below limit of detection, proportion of patients with low titers below lower limit of quantitation, viral load over time and virologic outcomes according to baseline viral load (>$10^4$, >$10^5$, >$10^6$, or >$10^7$ copies per milliliter).

# 9. Data Issues

There is no observed data issue while formulating the statistical analysis plan. Should any data issue identified after the SAP is finalized will be documented in regard to the handling of the data issue.

# 10. References

1 ICH. Statistical Principles for Clinical Trials, Guideline E9, 1998. Available at http://www.emea.eu.int/pdfs/human/ich/036396en.pdf

2 CPMP. Points to Consider on Missing Data. EMEA: London, 2001. Available at http://www.emea.eu.int/pdfs/human/ewp/177699EN.pdf

3 Phillips A and Haudiquet V. ICH E9 guideline "Statistical principles for clinical trials": a case study. Statistics in Medicine 2003; 22:1-11

4 Brown D J. ICH E9 guideline "Statistical principles for clinical trials": a case study. Response to A. Phillips and V. Haudiquet. Statistics in Medicine 2003; 22:13-17

5 Phillips A, Ebbutt A, France L, Morgan D, Ireson M, Struthers L and Heimann G. Issues in applying recent CPMP "Points to Consider" and FDA guidance documents with biostatistical implications. Pharmaceutical Statistics 2003; 2:241-251

6 Senn S. Statistical Issues in Drug Development. John Wiley & sons (Chichester), 1997.

7 Chow S-C and Liu J-P. Design and Analysis of Clinical Trials: Concepts and Methodologies. John Wiley & sons (New York), 1998.

8 Brown H and Prescott R. Applied Mixed Models in Medicine. John Wiley & sons (Chichester), 1999.

9 Fairclough D L. Design and Analysis of Quality of Life Studies in Clinical Trials. Chapman & Hall/CRC, 2002.

10 Green S, Benedetti J and Crowley J. Clinical Trials in Oncology (2nd edition). Chapman & Hall/CRC, 2002.

11 McEntegart D. Forced randomization when using interactive voice response systems. Applied Clinical Trials October 2003; 50-58.

12 CPMP. Points to Consider on Adjustment for Baseline Covaraites. EMEA: London, 2003

13 ICH. ICH E3 Guideline: Structure and Content of Clinical Study Reports Questions & Answers, 2012. Available at http://www.ich.org/fileadmin/Public_Web_Site/ICH_Products/Guidelines/Efficacy/E3/E3_QAs_R1_Step4.pdf

14 Lehmacher, W. and Wassmer, G. (1999). Adaptive sample size calculation in group sequentialtrials. Biometrics 55, 1286-1290.

15 Bauer, P. and Koehne, K. (1994). Evaluation of experiments with adaptive interim analyses. Biometrics 50, 1029-1041. Correction Biometrics 52, (1996), 380.

16 Simes, R.J. (1986). An improved Bonferroni procedure for multiple tests of signicance.Biometrika 73, 751-754.

17 Efron, B. The Efficiency of Cox's Likelihood Function for Censored Data, Journal of the American Statistical Association, 1977; 72, 557–565.

18 J.H. Beigel, K.M. Tomashek, L.E. Dodd, et al. Remdesivir for the Treatment

of Covid-19
— Final Report. The New England Journal of Medicine. 2020.

19 https://www.accessdata.fda.gov/drugsatfda_docs/nda/2020/214787Orig1s000Sumr.pdf.

20 Lan KKG, DeMets DL. Discrete sequential boundaries for clinical trials. Biometrika. 1983; 70:659-663.

# 11. Appendices

## Appendix 1: Document History

| Document Version, Status, Date | Summary/Reason for Changes |
|---|---|
| Version 1, Final, 25 April 2021 | Not applicable; the first version |

# Appendix 2: Schedule of Assessments

## . Schedule for Screening Visit

| Study Process | Screening Period | Comments |
|---|---|---|
| Visit | V1 | |
| Visit day | D-3~ D-1 | |
| Informed consent | X | |
| Demographics | X | Including birth date, sex, race, ethnicity and geographic region. |
| Medical history | X | Medical history within 30 days prior to signing the ICF including:<br>– The day of onset of COVID-19 signs and symptoms.<br>– History of medication allergies.<br>– History of medical conditions including but not limited to chronic oxygen requirement prior to onset of COVID-19.<br>– Ask if they are participating in another clinical trial or plan to enroll in another clinical trial in the next 30 days. |
| Physical examination | X | Testing items see protocol Section 7.2;<br>Physical examination is focused on lung auscultation. |
| β-HCG | X | For non-postmenopausal female subjects or who have not undergone surgical sterilization |
| Serum chemistry | X | Testing items see protocol Section 7.2. |
| SARS-CoV-2 infection confirmation | X | Biological samples (not limited to any specific type) collected within 72 hours before randomization is laboratory-confirmed as SARS-CoV-2 infection (PCR, etc.). |
| Vital signs | X | Include body temperature, pulse rate, blood pressure, respiratory rate in semi-supine position |
| Diagnosis of severe COVID-19 infection | X | Use one of following criteria: 1) respiratory rate; 2) $SpO_2$; 3) $PaO_2/FiO_2$ or $SpO_2/FiO_2$; 4) chest X-ray or CT. Refer to protocol Section 4.1 for details. |
| 8-Point Ordinal Scale | X | |
| Inclusion/Exclusion criteria review | X | |
| AEs | X | Record the AEs from after obtaining the Informed Consent |
| Prior treatments within the last 30days | X | |

## . Schedule for Treatment Visit

| Study Process | Treatment Period | Comments |
|---|---|---|
| Visit | V2 | |
| Visit day | D1 | |
| Randomization | X | |
| Height | X | |
| Weight | X | |
| Vital signs | X | Refer to Protocol Section 7.2.2.<br>At 15±5, 30±5, 45±5, 60±5, 90±10, 120±10, 150±10, 180±10 minutes after the beginning of infusion (i.e. every 15±5 minutes for the first hour of infusion). If infusion time is prolonged, the participant will continue to be monitored every hour for at least 2 hours post infusion. |
| Targeted physical examination | Are performed only when needed to evaluate possible adverse events. | |
| 12-lead ECG | X | Refer to Protocol Section 7.2.3<br>Test within 0.5 h (± 5 min) before infusion and at the end of infusion.<br>Additional ECG examination is at the discretion of the Investigator per standard clinical practice or as clinically indicated. |
| Hematology | X | Testing items see Protocol Section 7.2.5.<br>Sample will be collected before infusion. |
| Serum chemistry | X | |
| Urinalysis | X | |
| Coagulation test | X | |
| IL-6 | X | |
| Ferritin | X | |
| Procalcitonin | X | |
| Thrombin time | X | |
| D-dimer | X | |
| Serological samples for anti-spike protein antibodies | X | Blood samples will be collected within 0.5 h (± 5 min) before infusion. |
| Immunogenicity (ADA) blood samples | X | |
| PK samples | X | Intensive sampling of Phase II part: |

| Study Process | Treatment Period | Comments |
|---|---|---|
| | | – Within 0.5 h (± 5 min) before the beginning of infusion; 1 h (±10 min) post end of infusion. |
| Blood for PCR SARS-CoV-2 test | X | Sample will be collected before infusion. |
| NP or OP swab for SARS-CoV-2 test | X | Sample will be collected before infusion.<br>NP swab preferred. If NP swab cannot be obtained, OP swab may be used. |
| $SpO_2$ | X | Record $SpO_2$ before infusion, and 1 h (±10 min), 2 h (±10 min), 3 h (±10 min) after the end of infusion.<br>Also record oxygen supplementation: low flow oxygen, high flow oxygen, or other. |
| 8-Point Ordinal Scale | X | To evaluate within 30 ~ 60 min before infusion. |
| Administration of SCTA01/Placebo | X | The dose should be given within 24 hours after randomization. |
| AEs | X | |
| Concomitant medications | X | Items see previous table. |

## . Schedule for Follow-up Visit

| Study Process | Follow-up Period | | | | | | | | | | | | Comments |
|---|---|---|---|---|---|---|---|---|---|---|---|---|---|
| Visit | V3 | V4 | V5 | V6 | V7 | V8 | V9 | V10 | V11 | V12 | V13 | Early Withdraw | |
| Visit day | D2 | D3 | D5 | D8 | D11 | D15 | D22 | D29 | D60 | D120 | D150 | | |
| Window period (±day) | ±0 d | ±0 d | ±1 d | ±1 d | ±1 d | ±2 d | ±3 d | ±3 d | ±3 d | ±7 d | ±7 d | | |
| Vital signs | Daily until discharge | | | | | | | | | | | | Testing items see Section 7.2.2. |
| Targeted physical examination | Performed only when needed to evaluate possible adverse events | | | | | | | | | | | | Only performed while patient is in hospital or attends an in-person visit. |
| β-HCG | Only when potential pregnancy is suspected and at the end of the study | | | | | | | | | | | X | |
| 12-lead ECG | | X | | X | | X | | X | | X | | X | Testing items see Section 7.2.3 and 7.2.5.<br>Only performed while patient is in hospital or attends an in-person visit.<br>Additional examination is at the discretion of the Investigator. |
| Hematology | | X | | X | | X | | X | | X | | X | |
| Urinalysis | | X | | X | | X | | X | | X | | X | |
| Serum chemistry | | X | | X | | X | | X | | X | | X | |
| Coagulation test | | X | | X | | X | | X | | X | | X | |
| IL-6 | | X | | X | | X | | X | | X | | X | |
| Ferritin | | X | | X | | X | | X | | X | | X | |

| **Study Process** | **Follow-up Period** | | | | | | | | | | | | **Comments** |
|---|---|---|---|---|---|---|---|---|---|---|---|---|---|
| Visit | V3 | V4 | V5 | V6 | V7 | V8 | V9 | V10 | V11 | V12 | V13 | Early Withdraw | |
| Visit day | D2 | D3 | D5 | D8 | D11 | D15 | D22 | D29 | D60 | D120 | D150 | | |
| Window period (±day) | ±0 d | ±0 d | ±1 d | ±1 d | ±1 d | ±2 d | ±3 d | ±3 d | ±3 d | ±7 d | ±7 d | | |
| Procalcitonin | | X | | X | | X | | X | | X | | X | |
| Thrombin time | | X | | X | | X | | X | | X | | X | |
| D-dimer | | X | | X | | X | | X | | X | | X | |
| Immunogenicity blood samples | | | | | | | | X | | X | | X | |
| PK samples | | | | X[1, 2] | | X[1] | | X[1, 2] | | | | X[1, 2] | [1]: Intensive sampling of Phase II part: Blood samples will be collected in the same 30 subjects as the Table 3.<br>[2]: Sparse sampling of Phase II part: Blood samples will be collected in the remaining subjects (excluding the 30 subjects with intensive PK sampling) of each group of Phase II part. |

| **Study Process** | **Follow-up Period** | | | | | | | | | | | | **Comments** |
|---|---|---|---|---|---|---|---|---|---|---|---|---|---|
| Visit | V3 | V4 | V5 | V6 | V7 | V8 | V9 | V10 | V11 | V12 | V13 | Early Withdraw | |
| Visit day | D2 | D3 | D5 | D8 | D11 | D15 | D22 | D29 | D60 | D120 | D150 | | |
| Window period (±day) | ±0 d | ±0 d | ±1 d | ±1 d | ±1 d | ±2 d | ±3 d | ±3 d | ±3 d | ±7 d | ±7 d | | |
| Blood for SARS-CoV-2 test | | X | | X | | X | | X | | X | | X | Only performed while patient is in hospital or attends an in-person visit. |
| NP or OP swab for SARS-CoV-2 test | | X | X | X | X | X | | X | | X | | X | Continue testing at the scheduled time-point while hospitalized or attends an in-person visit, until the NP or OP swab samples viral RNA test is negative.<br>NP swab preferred. If NP swab can't be obtained, OP swab may be used. |
| Sequencing the gene encoding spike protein | Will be tested in subjects who do not achieve a reduction in viral shedding or persistence viral presence or have a rebound in viral shedding following suppression | | | | | | | | | | | | Using reserved NP or OP swab samples. No additional sampling is required. |
| 8-Point Ordinal Scale | Daily until discharge | | | | | X | X | X | | | | X | Only performed while patient is in hospital or attends an in-person visit, otherwise performed by |

| Study Process | Follow-up Period | | | | | | | | | | | | Comments |
|---|---|---|---|---|---|---|---|---|---|---|---|---|---|
| Visit | V3 | V4 | V5 | V6 | V7 | V8 | V9 | V10 | V11 | V12 | V13 | Early Withdraw | |
| Visit day | D2 | D3 | D5 | D8 | D11 | D15 | D22 | D29 | D60 | D120 | D150 | | |
| Window period (±day) | ±0 d | ±0 d | ±1 d | ±1 d | ±1 d | ±2 d | ±3 d | ±3 d | ±3 d | ±7 d | ±7 d | | |
| | | | | | | | | | | | | | phone call to assess clinical status (ordinal scale).<br>The date and cause of discharge or re-hospitalization after discharge should be recorded. |
| Concomitant medications | Daily until discharge | | | | | | | | | | | | |
| $SpO_2$ | Daily until discharge | | | | | | | | | | | | Report the best reading if multiple readings are obtained in a day.<br>Also record oxygen supplementation: low flow oxygen, high flow oxygen, or other. |
| AEs | Daily until discharge and biweekly via phone call after discharge until 150 days. | | | | | | | | | | | | |
| ADE | Monitor for new infections and adverse events until 150 days (if discharged, via biweekly telephone call, in-person or virtual visit). | | | | | | | | | | | | |

## Appendix 3: Calculation of time-weighted average change from baseline between SCTA01 group and placebo group in viral shedding.

Time-weighted average change from baseline of viral load will be calculated using trapezoidal rule, i.e., area under the curve for change from baseline at each time point from day 1 to last observation divided by the number of days from day 1 to day of last observation.

The following calculation will be used:

$$TWA_{[0-k]} = \frac{1}{2(t_k - t_0)} \sum_{i=1}^{k} (t_i - t_{i-1})(D_i + D_{i-1})$$

Where $TWA_{[0-k]}$ is the time-weighted average change from baseline in viral load for day $t_k$ $(k \geq 1)$. $t_0 = 1$ and is the baseline day. $t_i$ is the i-th post-baseline day. $D_i$ is the change from baseline viral load on i-th post-baseline day.

If $D_i$ is not available per protocol or missing due to failed test or other reasons, only the time points with non-missing values will be included into the calculation. For example, if the $TWA_{[0-8]}$ is calculated when the day 1,2,4 and 6 values are not available per the protocol schedule of events , and day 5, day 7 values are missing due to failed tests, the result should be calculated as:

$$TWA_{[0-8]} = \frac{1}{2(t_8 - t_0)} [(t_3 - t_0)(D_3 + D_0) + (t_8 - t_3)(D_8 + D_3)]$$
