# Supplementary material for: Fc-Modified Antibody in Hospitalized Severe COVID-19 Patients
Source: Vaccines (Basel). 2025 Mar 31;13(4):372. doi: 10.3390/vaccines13040372 (PMC12031629; doi:10.3390/vaccines13040372)

Supplemental Material

Table S1. Summary of AEs in ≥ 5% of Patients by SOC and PT

	**SCTA01 15 mg/kg N=33, n (%)**	**SCTA01 50 mg/kg N=34, n (%)**	**Placebo N=35, n (%)**
Overall	9 (27.3)	16 (47.1)	11 (31.4)
Gastrointestinal disorders			
Constipation	6 (18.2)	11 (32.4)	4 (11.4)
General disorders and administrative site conditions			
Pyrexia	3 (9.1)	6 (17.6)	2 (5.7)
Hypothermia	1 (3.0)	4 (11.8)	1 (2.9)
Metabolism and nutrition disorders			
Hyperkalaemia	1 (3.0)	6 (17.6)	1 (2.9)
Hyperglycemia	2 (6.1)	4 (11.8)	3 (8.6)
Psychiatric disorders			
Insomnia	2 (6.1)	5 (14.7)	4 (11.4)
Anxiety	0	4 (11.8)	6 (17.1)
Vascular disorders			
Hypertension	2 (6.1)	3 (8.8)	4 (11.4)
Hypotension	1 (3.0)	3 (8.8)	3 (8.6)
Blood and lymphatic system disorders			
Neutrophilia	1 (3.0)	5 (14.7)	3 (8.6)
Leukocytosis	1 (3.0)	4 (11.8)	3 (8.6)

Abbreviations: N = total number of patients; n = number of patients; PT = preferred term; SOC = system organ class.

Table S2. Summary of SAEs by SOC and PT

	SCTA01 15 mg/kg N=33, n (%)	SCTA01 50 mg/kg N=34, n (%)	Placebo N=35, n (%)
Overall	7 (21.2)	5 (14.7)	5 (14.3)
Respiratory, thoracic, and mediastinal disorders	6 (18.2)	3 (8.8)	4 (11.4)
Respiratory failure	1 (3.0)	3 (8.8)	1 (2.9)
Pulmonary embolism	3 (9.1)	0	1 (2.9)
Acute respiratory failure	1 (3.0)	0	1 (2.9)
Pneumomediastinum	1 (3.0)	0	0
Pneumothorax	1 (3.0)	0	1 (2.9)
Dyspnea	0	0	1 (2.9)
Infections and infestations	3 (9.1)	3 (8.8)	2 (5.7)
Sepsis	1 (3.0)	2 (5.9)	0
Septic shock	0	2 (5.9)	0
Bacterial sepsis	1 (3.0)	0	0
Pulmonary sepsis	1 (3.0)	0	0
Gastroenteritis	0	0	1 (2.9)
Pneumonia	0	0	1 (2.9)
Tracheobronchitis	0	0	1 (2.9)
Urinary tract infection	0	0	1 (2.9)
Cardiac disorders	2 (6.1)	1 (2.9)	2 (5.7)
Cardio-respiratory arrest	2 (6.1)	1 (2.9)	1 (2.9)
Cardiac failure	1 (3.0)	0	0
Acute left ventricular failure	0	0	1 (2.9)
Blood and lymphatic system disorders	0	1 (2.9)	0
Anaemia	0	1 (2.9)	0
General disorders and administrative site conditions	1 (3.0)	0	0
Multiple organ dysfunction syndrome	1 (3.0)	0	0
Metabolism and nutrition disorders	0	1 (2.9)	0
Hyperkalaemia	0	1 (2.9)	0
Renal and urinary disorders	0	1 (2.9)	1 (2.9)
Acute kidney injury	0	1 (2.9)	1 (2.9)
Vascular disorders	0	1 (2.9)	0
Embolism venous	0	1 (2.9)	0

Abbreviations: N = total number of patients; n = number of patients; PT = preferred term; SOC = system organ class.

Figure S1. Distribution of patients on the 8-point scale

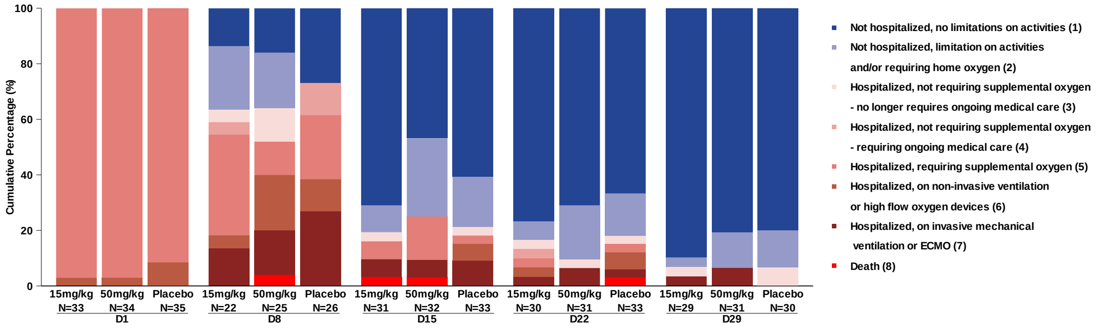

Supplement: Supplementary file 1 [file vaccines-13-00372-s001.zip › Supplementary Tables and Figure.pdf]
